# Interpersonal value regulates infection-risky social contact across 61 societies

**DOI:** 10.1073/pnas.2615739123

**Published:** 2026-10-15

**Authors:** Hongyu Sun, Giuliana Spadaro, Sofia Pelica, Roza G. Kamiloğlu, Loes Abrahams, Joshua M. Ackerman, Savannah Adams, Laith Al-Shawaf, Alexandra Alvergne, Benjamin Amponsah, Njål Andersen, Jan Antfolk, Derya Atamtürk Duyar, Songshi Bai, María Teresa Barbato, Sergio Barbosa, Pat Barclay, D. Vaughn Becker, Beatriz B. de Souza, Paulo Sérgio Boggio, Robert Böhm, Watcharaporn Boonyasiriwat, Andrea S. Camperio Ciani, Ali Rıza Can, Trevor I. Case, Kai Qin Chan, Luis Sebastian Contreras-Huerta, Paul Conway, Coralie Chevallier, Éloïse Côté, Sam P. Dale, Florencio Delgado Espinoza, Daniela Dlouhá, Izzet Duyar, Christian T. Elbæk, Qiran Fan, Ana María Fernández, Diogo C. S. Ferreira, Daniel M. T. Fessler, Pablo Ezequiel Flores-Kanter, Natalia Frankowska, Ana Luísa Freitas, Márta Fülöp, Marcello Gallucci, Zachary H. Garfield, Margarida V. Garrido, Ekmel Gecer, Macarena González-Portilla, Dmitry Grigoryev, Minh-Tri Ha, Brahim Hamdaoui, Sahar Nadeem Hamid, Lina S. Hansson, Jan Havlíček, Benjamin E. Hilbig, Ian Hohm, Szilárd Holka, Minsung Hong, Yutaka Horita, Hsin-Ping Hsu, Yoel Inbar, Keiko Ishii, Olivia M. James, Tomasz Jarmakowski, Tingting Ji, William Jiménez-Leal, Shanmukh Kamble, Šárka Kaňková, Sonya Karabeliova, Honnête-Nyandu Kasali, Jinseok P. Kim, Toko Kiyonari, John Manuel R. Kliatchko, Nils Köbis, Ferenc Kocsor, Tobias L. Kordsmeyer, Tom R. Kupfer, Lorena Lázaro Cuesta, Christine Xiang Ru Leong, David M. G. Lewis, Debra Lieberman, Hongtao Liu, Hernán López-Morales, Claudio López-Calle, Ingunn Olea Lund, Fiorella Macchiavello, Olga Marchenko, Urszula M. Marcinkowska, Gabriela Mariana Marcu, Norbert Meskó, Suyash Mishra, Kaloyan Mitev, Asako Miura, Fredy S. Monge Rodríguez, Leticia Y. Morello, Bruna E. F. Mota, Damian R. Murray, Felix-kingsley Obialo, Oluyinka Ojedokun, Mats J. Olsson, Gonzalo Palomo-Vélez, Folco Panizza, Fernanda Naomi Pantaleão, Lars Penke, Daniel Penn, Andrea Perinetti, Gerasimos Prodromitis, Ádám Putz, Angelica Quiroga-Garza, Nagireddy Neelakanteswar Reddy, Marc Eric S. Reyes, Felipe Rojas-Thomas, Rachel Rothschild, Kaelyn Sabree, Fatima Zahra Sahli, Cécile Sarabian, André Sarmento, Ya Hui Michelle See, Theresia Julius Shavega, Zubaida Shebani, Agnes Sianipar, Divita Singh, Sajjad Sojoudi, Francisco Leonardo Soler-Anguiano, Anna Soveri, Richard J. Stevenson, Steve Stewart-Williams, Ferran Suay, Eunkook M. Suh, Jean-Roger Syayipuma Kambere, Ohad Szepsenwol, Carmit T. Tadmor, Tair Tager-Shafrir, Elahe Tavakoli-Berg, Shruti Tewari, Arnaud Tognetti, Antonia Tsitseli, Ignacio Leandro Uriarte, Martijn Van Heel, Florian van Leeuwen, Alfio Ventura, Jin Wang, X. T. Wang, Joseph Watts, Junhui Wu, Su-Ling Yeh, Eric Kenson Yau, Victoria Wai Lan Yeung, Janis H. Zickfeld, Joshua M. Tybur

**Affiliations:** ^a^ Department of Experimental and Applied Psychology, Vrije Universiteit Amsterdam, Amsterdam 1081 BT, The Netherlands; ^b^Institute for Brain and Behavior, Amsterdam 1081 BT, The Netherlands; ^c^ Tilburg School of Social and Behavioral Sciences, Department of Social Psychology, Tilburg University, Tilburg 5037 AB, The Netherlands; ^d^ Department of Psychology, University of Michigan, Ann Arbor, MI 48109; ^e^Department of Psychology, University of Colorado, Colorado Springs, CO 80918; ^f^ Institute for Advanced Study in Toulouse, Toulouse School of Economics, University of Toulouse Capitole, Toulouse 31080 Cedex 6, France; ^g^ Institute for Evolutionary Sciences, Université de Montpellier, CNRS, Institut de Recherche pour le Développement, École Pratique des Hautes Études, Montpellier 34095 Cedex 5, France; ^h^ School of Anthropology and Museum Ethnography, University of Oxford, Oxford OX2 6PE, United Kingdom; ^i^ Psychology Department, University of Ghana, Legon LG 25, Ghana; ^j^ Department of International Business, Norges Teknisk-Naturvitenskapelige Universitet, Ålesund 7491, Norway; ^k^ Psychology, Department of Humanities, Åbo Akademi University, Turku 20500, Finland; ^l^ Department of Anthropology, İstanbul University, Istanbul 34452, Türkiye; ^m^ State Key Laboratory of Cognitive Science and Mental Health, Institute of Psychology, Chinese Academy of Sciences, Beijing 100101, China; ^n^ Department of Psychology, University of Chinese Academy of Sciences, Beijing 100049, China; ^o^ Laboratorio de Evolucion y Relaciones Interpersonales, Universidad de Santiago de Chile, Santiago 9170022, Chile; ^p^ Escuela de Medicina y Ciencias de La Salud, Universidad del Rosario, Bogotá 111711, Colombia; ^q^ Department of Psychology, University of Guelph, Guelph, ON N1G 2W1, Canada; ^r^ Department of Psychology, Arizona State University, Tempe, AZ 85287; ^s^ Social and Cognitive Neuroscience Laboratory, Mackenzie Presbyterian University, São Paulo 01302-907, Brazil; ^t^ National Institute of Science and Technology on Social and Affective Neuroscience, Conselho Nacional de Desenvolvimento Científico e Tecnológico, São Paulo 01302-907, Brazil; ^u^ Department of Occupational, Economic and Social Psychology, Faculty of Psychology, University of Vienna, Vienna 1010, Austria; ^v^ Department of Banking and Finance, University of Innsbruck, Innsbruck 6020, Austria; ^w^ The Faculty of Psychology, Chulalongkorn University, Bangkok 10330, Thailand; ^x^ Department of Philosophy, Sociology, Education and Applied Psychology, University of Padua, Padua 35131, Italy; ^y^ Department of Anthropology, Hatay Mustafa Kemal University, Hatay 31060, Turkey; ^z^ School of Psychological Sciences, Macquarie University, Sydney, NSW 2109, Australia; ^aa^Department of Psychology, James Cook University, Singapore 387380, Singapore; ^bb^ Center for Social and Cognitive Neuroscience, School of Psychology, Universidad Adolfo Ibáñez, Viña del Mar 2520000, Chile; ^cc^ Center of Social Conflict and Cohesion Studies, Santiago 7500011, Chile; ^dd^ School of Psychology, University of Southampton, Southampton SO17 1BJ, United Kingdom; ^ee^ Institut Jean Nicod, Département d’études Cognitives, Ecole Normale Supérieure, Université Paris Sciences et Lettres, École des Hautes Études en Sciences Sociales, CNRS, Paris 75005, France; ^ff^ Department of Psychology, University of Toronto, Toronto, ON M5S 1A1, Canada; ^gg^ School of Psychology, Murdoch University, Murdoch, WA 6150, Australia; ^hh^ Anthropology Department, Universidad San Francisco de Quito, Quito 170901, Ecuador; ^ii^ Department of Philosophy and History of Science, Faculty of Science, Charles University, Prague 128 00, Czech Republic; ^jj^ Department of Management, Aarhus University, Aarhus 8210, Denmark; ^kk^ Department of Psychology, Tulane University, New Orleans, LA 70118; ^ll^ Psychology Department, Universidade Federal de Sergipe, São Cristóvão 49100-000, Brazil; ^mm^ Department of Anthropology, University of California, Los Angeles, CA 90095; ^nn^ University of California, Los Angeles Center for Behavior, Evolution and Culture, Los Angeles, CA 90095; ^oo^ University of California, Los Angeles Bedari Kindness Institute, Los Angeles, CA 90095; ^pp^ Escuela de Posgrado, Universidad Continental, Lima 15046, Peru; ^qq^ Department of Social Psychology, Szkoła Wyższa Psychologii Społecznej University, Warsaw 03-815, Poland; ^rr^ Center for Research on Biological Basis of Social Behavior, Szkoła Wyższa Psychologii Społecznej University, Warsaw 03-815, Poland; ^ss^ Hungarian Research Network Institute of Cognitive Neuroscience and Psychology, Research Centre of Natural Sciences, Budapest 1117, Hungary; ^tt^ Károli Gáspár University of the Reformed Church in Hungary, Budapest 1091, Hungary; ^uu^ Department of Psychology, Università Degli Studi Di Milano-Bicocca, Milan 20126, Italy; ^vv^Africa Institute for Research in Economics and Social Sciences, University Mohammed VI Polytechnic, Rabat 11103, Morocco; ^ww^ Department of Psychology, Instituto Universitário de Lisboa, Centro de Investigação e Intervenção Social, Lisbon 1649-026, Portugal; ^xx^ Psychology Department, Marmara University, Istanbul 34722, Türkiye; ^yy^ Department of Psychobiology, Faculty of Psychology, Universitat de València, Valencia 46010, Spain; ^zz^ Center for Socio-Cultural Research, National Research University Higher School of Economics (HSE) University, Moscow 101000, Russia; ^aaa^School of Business, International University, Ho Chi Minh City 700000, Vietnam; ^bbb^ Vietnam National University, Ho Chi Minh City 700000, Vietnam; ^ccc^ Faculty of Humanities and Social Sciences, Ibn Tofail University, Kénitra 14000, Morocco; ^ddd^ School of Social Sciences & Humanities, National University of Sciences and Technology, Islamabad 44000, Pakistan; ^eee^ Division of Psychology, Department of Clinical Neuroscience, Karolinska Institutet, Stockholm 171 77, Sweden; ^fff^ Osher Center for Integrative Health, Department of Clinical Neuroscience, Karolinska Institutet, Stockholm 171 77, Sweden; ^ggg^ Department of Psychology, Stockholm University, Stockholm 106 91, Sweden; ^hhh^ Department of Zoology, Faculty of Science, Charles University, Prague 128 00, Czech Republic; ^iii^Department of Psychology, University Kaiserslautern-Landau, Landau 76829, Germany; ^jjj^ Department of Psychology, University of British Columbia, Vancouver V6T 1Z4, Canada; ^kkk^ Department of Psychiatry and Psychotherapy, Semmelweis University, Budapest 1083, Hungary; ^lll^ Department of Psychology, Yonsei University, Seoul 03722, Republic of Korea; ^mmm^ Department of Psychology, Teikyo University, Hachioji City 192-0395, Japan; ^nnn^ Department of Psychology, National Cheng Kung University, Tainan 701, Taiwan; ^ooo^ Department of Cognitive and Psychological Sciences, Nagoya University, Nagoya 464-8601, Japan; ^ppp^ Department of Social and Environmental Psychology, Institute of Psychology, Nicolaus Copernicus University, Toruń 87-100, Poland; ^qqq^ School of Psychology, Nanjing Normal University, Nanjing 210023, China; ^rrr^Psychology Department, Universidad de los Andes, Bogotá 111711, Colombia; ^sss^Cognition Lab, Universidad de los Andes, Bogotá 111711, Colombia; ^ttt^ Department of Psychology, Karnatak University, Dharwad 580003, India; ^uuu^Department of Psychology, Sofia University “St. Kliment Ohridski”, Sofia 1504, Bulgaria; ^vvv^ École de psychologie, Université Laval, Québec, QC G1V 0A, Canada; ^www^Faculté de Psychologie et des Sciences de l’Éducation, Université Libre des Pays Des Grands Lacs, Goma B.P. 368, République Démocratique du Congo; ^xxx^ School of Psychology, University of Sussex, Brighton BN1 9RH, United Kingdom; ^yyy^ Department of Social Informatics, Aoyama Gakuin University, Kanagawa 150-8366, Japan; ^zzz^ Department of Behavioral Science, Faculty of Arts and Letters, University of Santo Tomas, Manila 1008, Philippines; ^aaaa^Research Center Trustworthy Data Science and Security, University Duisburg-Essen, Duisburg 47057, Germany; ^bbbb^ Center for Humans and Machines, Max Planck Institute for Human Development, Berlin 14195, Germany; ^cccc^ Institute of Psychology, Faculty of Humanities and Social Sciences, University of Pécs, Pecs 7624, Hungary; ^dddd^ Institute of Psychology, University of Goettingen, Göttingen 37073, Germany; ^eeee^ Leibniz ScienceCampus Primate Cognition, Göttingen 37073, Germany; ^ffff^ Department of Psychology, Nottingham Trent University, Nottingham NG1 4FQ, United Kingdom; ^gggg^ Faculty of Medicine, National University of Mar del Plata, Mar del Plata B7600, Argentina; ^hhhh^School of Psychology, University of Nottingham Malaysia, Semenyih 43500, Malaysia; ^iiii^ Centre for Healthy Ageing, Health Futures Institute, Murdoch University, Murdoch, WA 6150, Australia; ^jjjj^ Department of Psychology, University of Miami, Coral Gables, FL 33146; ^kkkk^ Institute of Basic and Applied Psychology and Technology, National University of Mar del Plata, Mar del Plata B7600, Argentina; ^llll^ Department of Education, University of Cuenca, Cuenca 010107, Ecuador; ^mmmm^ PsychGen Center for Genetic Epidemiology and Mental Health, and Department of Child Health and Development, Norwegian Institute of Public Health, Oslo 0213, Norway; ^nnnn^ Department of Psychology, University of Oslo, Oslo 0373, Norway; ^oooo^ Laboratory for Cognitive Studies of Communication, Moscow State Linguistic University, Moscow 119034, Russia; ^pppp^ Department of Environmental Health, Jagiellonian University Medical College, Kraków 31-531, Poland; ^qqqq^ Department of Psychology, Lucian Blaga University of Sibiu, Sibiu 550024, Romania; ^rrrr^Scientific Research Group in Neuroscience “Dr. Gheorghe Preda” Clinical Psychiatry Hospital, Sibiu 550082, Romania; ^ssss^ Humanities and Social Sciences, Indian Institute of Management Indore, Indore 453556, India; ^tttt^ Graduate School of Human Sciences, The University of Osaka, Suita, Osaka 565-0871, Japan; ^uuuu^ Department of Psychology, Universidade Da Coruña, A Coruña 15071, Spain; ^vvvv^People-Environment Research Group, Centro de Innovación Tecnolóxica en Edificación E Enxeñería Civil, A Coruña 15071, Spain; ^wwww^Social and Affective Neuroscience National Institute, São Paulo 01302-907, Brazil; ^xxxx^ Centre for Creativity and Entrepreneurial Studies, Dominican University Ibadan, Ibadan 200213, Nigeria; ^yyyy^ Department of Pure and Applied Psychology, Adekunle Ajasin University, Akungba-Akoko 342111, Nigeria; ^zzzz^ Department of Psychology, University of Pretoria, Pretoria 0002, South Africa; ^aaaaa^ Department of Psychology, University of Groningen, Groningen 9712 TS, The Netherlands; ^bbbbb^Molecular Mind Laboratory, Institutions, Markets, Technologies School for Advanced Studies Lucca, Lucca 55100, Italy; ^ccccc^ Department of Psychology, Panteion University of Social and Political Sciences, Athens 176 71, Greece; ^ddddd^ Department of Cognitive and Evolutionary Psychology, Faculty of Humanities and Social Sciences, University of Pécs, Pécs 7624, Hungary; ^eeeee^ Department of Psychology, Universidad de Monterrey, San Pedro Garza García 66238, Mexico; ^fffff^ Division of Social Sciences, Ahmedabad University, Ahmedabad 380009, India; ^ggggg^ Department of Psychology, College of Science, University of Santo Tomas, Manila 1008, Philippines; ^hhhhh^ Institute for Society and Genetics, University of California, Los Angeles, CA 90095; ^iiiii^Laboratory of Interdisciplinary Sport Sciences, Institut des Métiers du Sport, University Ibn Tofaïl, Kénitra 14000, Morocco; ^jjjjj^ Biosciences Department, Swansea University, Swansea SA2 8PP, United Kingdom; ^kkkkk^Department of Communication Studies, NOVA University Lisbon, Lisbon 1069-061, Portugal; ^lllll^ Department of Psychology, National University of Singapore, Singapore 119260, Singapore; ^mmmmm^ Department of Psychology and Special Education, The Open University of Tanzania, Dar Es Salaam 14110, Tanzania; ^nnnnn^ Psychology Department, Sultan Qaboos University, Muscat 123, Oman; ^ooooo^ Faculty of Psychology, Universitas Indonesia, Depok, Jawa Barat 16424, Indonesia; ^ppppp^ Cognition, Affect and Well-Being Lab, Universitas Indonesia, Depok, Jawa Barat 16424, Indonesia; ^qqqqq^ School of Arts and Sciences, Ahmedabad University, Ahmedabad 380009, India; ^rrrrr^ Faculty of Psychology, National Autonomous University of Mexico, Mexico City 04510, Mexico; ^sssss^Domaine des Sciences Psychologiques et de l’Éducation, Université Officielle de Ruwenzori, Butembo B.P. 560, République Démocratique du Congo; ^ttttt^ Department of Education and Educational Counseling, The Max Stern Yezreel Valley College, Emek Yezreel 1930600, Israel; ^uuuuu^ Center for Psychobiological Research, The Max Stern Yezreel Valley College, Emek Yezreel 1930600, Israel; ^vvvvv^ Department of Organizational Behavior, Coller School of Management, Tel Aviv University, Tel Aviv 6997801, Israel; ^wwwww^ Center for Environmental Economics – Montpellier, University of Montpellier, CNRS, Institut National de Recherche pour l’Agriculture, l’Alimentation et l’Environnement, Institut Agro, Montpellier 34060, France; ^xxxxx^Interzonal Specialized Maternal and Child Hospital ‘Vitorio Tetamanti’, Mar del Plata B7600, Argentina; ^yyyyy^ Department of Psychology, Faculty of Psychology and Educational Sciences, Vrije Universiteit Brussel, Brussels 1050, Belgium; ^zzzzz^Research Center Trustworthy Data Science and Security, University Alliance Ruhr, Duisburg 47057, Germany; ^aaaaaa^ Faculty of Computer Science, University of Duisburg-Essen, Duisburg 47057, Germany; ^bbbbbb^ School of Humanities and Social Science, The Chinese University of Hong Kong, Shenzhen 518172, China; ^cccccc^ School of Psychology Speech and Hearing, University of Canterbury, Christchurch 8140, New Zealand; ^dddddd^ Department of Linguistic and Cultural Evolution, Max Planck Institute for Evolutionary Anthropology, Leipzig 04103, Germany; ^eeeeee^ Department of Psychology, National Taiwan University, Taipei 10617, Taiwan; ^ffffff^ Department of Psychology, Lingnan University, Hong Kong 999077, China

**Keywords:** disease transmission, interpersonal contact, interpersonal value, evolutionary psychology

## Abstract

Interpersonal contact is essential for cooperation and care, but it also provides a route for pathogen transmission between individuals. Hence, a key challenge for social organisms involves maintaining beneficial social contact while also limiting exposure to infection risks. Existing proposals suggest that this trade-off is guided by the interpersonal value of specific social targets. In addition, ecological and cultural factors, including historical pathogen prevalence, individualism-collectivism, tightness–looseness, and relational mobility, might also shape how people avoid pathogens. To test whether and how infection-risky social contact varies with individual-level interpersonal value and these societal-level factors, we collected data from 47,756 participants across 61 societies. Participants were randomly assigned to picture a social target - either a friend, acquaintance, stranger, or enemy. They then rated their comfort with infection-risky contact with that target (e.g., drinking from the same water bottle) and whether they would touch the target when greeting them. In all societies, participants were more comfortable with infection-risky contact and more likely to engage in physical contact during greetings with targets of higher interpersonal value. Such associations varied only modestly across societies. Notably, the associations between infection-risky contact and societal-level factors depended on target interpersonal value and were concentrated among high-value targets—contact was lower where historical pathogen prevalence and tightness were greater and higher where individualism and relational mobility were greater. These findings suggest that infection-risky contact is primarily guided by the value of social targets, with ecological and cultural factors playing a lesser role.

## Significance

Balancing the benefits of social contact against infection risks is a fundamental challenge faced throughout human evolution. Prior work proposes that this trade-off is embedded in mental processes that track the interpersonal value of social partners. If this account is correct, the link between interpersonal value and infection-risky contact should be universal. We find that it is: Across 61 societies, people were consistently more willing to engage in infection-risky contact with high-value social partners. Diverse societal-level factors also related to infection-risky contact, but specifically for high-value targets rather than low-value ones. Findings support the universality of a social psychology of pathogen avoidance that considers the interpersonal value of targets while also being calibrated by societal factors.

Across species, pathogens have driven the evolution of myriad adaptations that function to mitigate infection risks (1). In humans, examples include psychological mechanisms that detect illness cues in others (e.g., skin lesions, lethargy, pallor) and subsequently motivate avoidance of those possessing such cues (2). Nevertheless, infected individuals sometimes do not produce detectable symptoms (2, 3). Further, even people who are not themselves infected can transmit any pathogens that might be present on their hands. Minimizing contact even with seemingly healthy others reduces the likelihood of infection, but completely avoiding infection-risky contact is not feasible. After all, everyday interactions—including sharing food, exchanging objects, and providing or receiving care—are essential for social life. Consequently, people must weigh the social benefits of contact with others against the corresponding infection risks of proximity, indirect contact (i.e., touching things that another person has touched), and direct contact (i.e., touching another person’s body). In sum, the challenge of maintaining beneficial contact while limiting exposure to infectious microbes is one that humans have had to navigate across all epochs and all ecologies (4, 5).

The recurring nature of this challenge suggests that humans may possess dedicated mental processes that regulate infection-risky contact by balancing infection costs against social benefits. A similar cost–benefit logic underlies other adaptive decisions. For instance, foraging decisions involve weighing nutritional and energetic returns against the time, energy, and predation risks required to obtain food (5, 6). Social interactions are structured similarly: People assign weight to other people’s welfare relative to their own, and they preferentially allocate benefits to those they value more relative to those they value less (4, 5, 7). Valuation processes have been proposed to integrate multiple features of an individual, including genetic relatedness, ability and likelihood to reciprocate, and formidability, into a welfare trade-off ratio (WTR)—a mental representation of interpersonal value (5, 8, 9). Evidence from both industrialized and forager societies is consistent with the existence of such a process: WTR relates to a number of emotions and behaviors relevant to valuation, including helping, cooperation, gratitude, and forgiveness (5, 7, 8, 10, 11). In addition, preliminary findings in a few populations suggest that interpersonal value might also relate to behaviors not typically considered in the cooperation literature—those relevant to pathogen transmission. For example, mothers report less disgust toward their own infants’ diapers than toward those of unrelated infants (12), foragers provide more care to those sick individuals who are expected to reciprocate (13, 14), and U.S. and Dutch participants are more comfortable engaging in interpersonal contact that risks microbe transmission (e.g., using the same towel) with people they value more than with people they value less (9, 15). If natural selection shaped mechanisms that use valuation to regulate pathogen avoidance, then the link between interpersonal value and infection-risky social contact should be universal across populations.

This proposal of a universal relation between interpersonal value and infection-risky social contact is distinct from a body of work proposing that human pathogen avoidance varies markedly across societies as a function of a specific ecological variable: historical pathogen prevalence. Findings in this area suggest that higher historical pathogen prevalence is associated with lower extraversion, less romantic kissing, and greater racial prejudice (16–19). These findings, however, face two notable challenges. First, many outcome variables (e.g., extraversion) examined in the literature have ambiguous consequences for infection risk. Second, the reported relations between pathogen prevalence and outcome variables are often not robust to corrections for societal nonindependence (20, 21). Given these challenges, the relation between ecological pathogen prevalence and behaviors with clear consequences for infectious disease remains unclear. Parasite-stress theory nonetheless makes a straightforward prediction: Infection-risky contact should be lower overall in societies with higher pathogen prevalence.

Parasite-stress theory also implies that pathogen prevalence might shape the relation between interpersonal value and infection-risky contact. It proposes that higher pathogen prevalence is associated with the tendency to interact more with high-value others such as friends and kin and less with low-value others such as strangers and outgroup members (17, 22, 23). Whereas existing work has examined covariation between societal-level pathogen prevalence and collectivism, family ties, and racial prejudice, none has tested whether pathogen prevalence differentially relates to willingness to engage in infection-risky contact with social partners differing on interpersonal value. Following parasite stress theory, the relation between interpersonal value and infection-risky contact should be stronger in societies with high pathogen prevalence than those with low pathogen prevalence. Specifically, pathogen prevalence should constrain contact with low-value targets more than with high-value ones.

In addition to ecological history, multiple cultural factors might also relate to infection-risky social contact (24–26). Tighter cultures, which emphasize adherence to norms and punishment of deviance (27), experienced fewer cases and deaths during the early period of the COVID-19 pandemic (28). Conversely, societies with greater relational mobility—the tendency to freely choose and replace social partners like friends (29)—experienced faster spread of COVID-19 (30). Collectivist values also might shape patterns of infection-risky contact by encouraging interactions with close others over those with distant others (31, 32). As with ecological pathogen prevalence, though, evidence for relations between cultural factors and infection-risky social contact is limited and indirect, and no work speaks to whether these same factors might moderate how interpersonal value relates to such contact.

## Empirical Investigation

We collected data from 42,756 participants across 61 societies. Each participant was asked to imagine a specific social target. To guarantee variation in the interpersonal value of those targets, we randomly assigned participants to think of a target from one of four folk relationship categories: their closest friend, an acquaintance, a stranger, or a person they know personally and dislike intensely (hereafter enemy). They completed a welfare trade-off task with this person in mind; results from this task were used to estimate WTR, with a greater value indicating that the participant values the target’s financial welfare more. They then reported i) their comfort with a set of everyday actions that risk exposure to the target’s microbes through close physical proximity or contact with the same objects, such as sitting close enough that one’s leg touches the target’s or drinking from the same water bottle as the target (hereafter contact comfort); and ii) their likelihood of physically touching the target during greetings, such as with handshakes, hugs, or kisses (hereafter contact greeting).

Using these data, the present study i) assessed cross-societal variation in contact comfort and contact greeting; ii) tested whether the relation between interpersonal value and these behaviors is universal across societies; iii) examined how historical pathogen prevalence and cultural factors relate to these behaviors; and iv) evaluated whether historical pathogen prevalence or cultural factors moderate any relation between interpersonal value and these behaviors. Analyses were first conducted using frequentist multilevel models. Results of these analyses are reported with parameter estimates, 95% CI, and *P-*values. To evaluate robustness, key findings were reexamined using Bayesian multilevel models accounting for societal nonindependence (see Data Analysis); these results are reported with posterior medians and 95% credible intervals (CrI). In the main text, we mainly focus on relations between WTR and the dependent measures. Detailed results concerning relationship categories are reported in *SI Appendix*, sections S3.3–S3.7.

## Results

### Does Infection-Risky Social Contact Vary Across Societies?

**Fig. 1.**
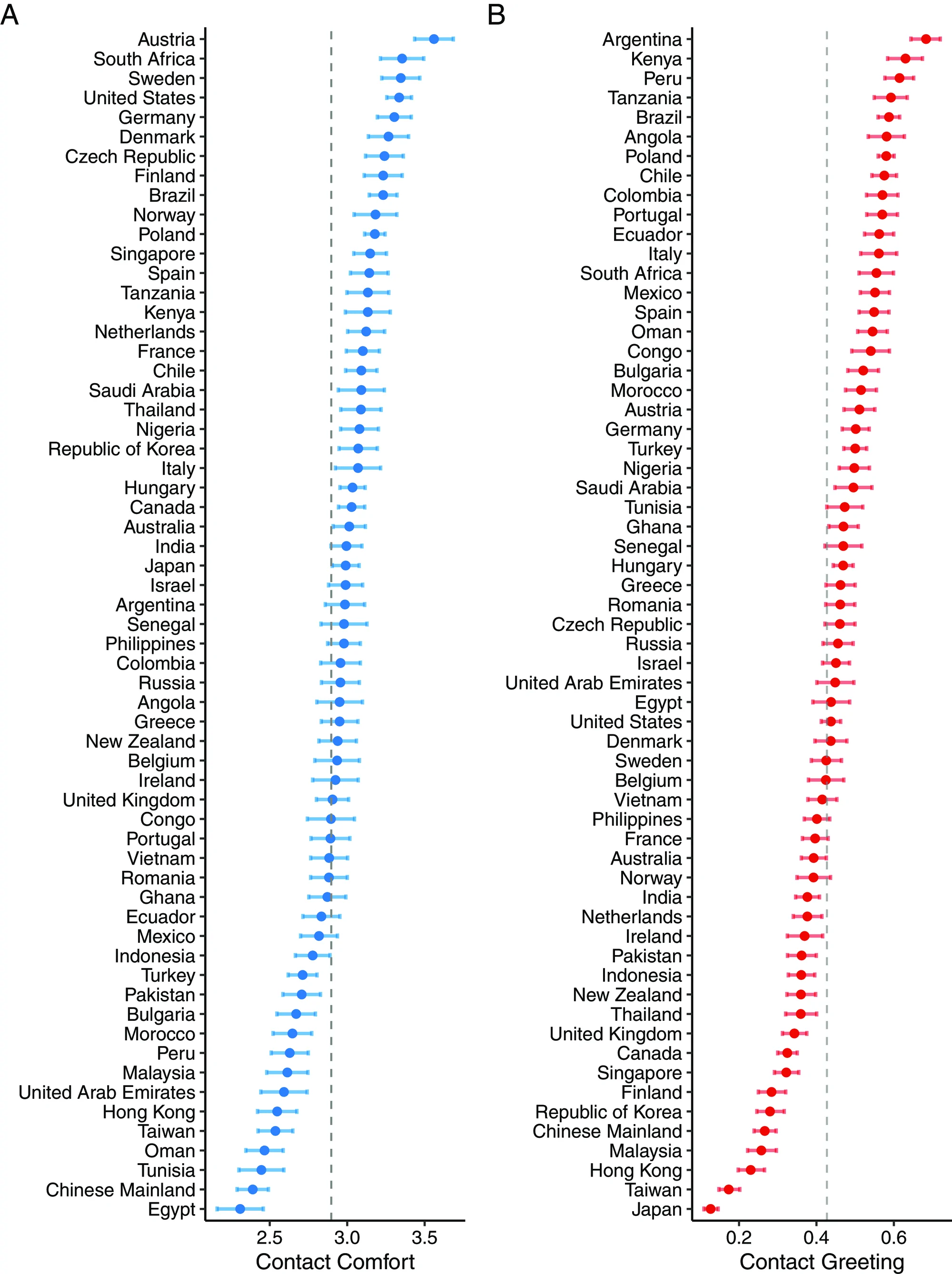
Cross-societal variation in contact comfort and contact greeting. (*A*) Estimated society-level means for contact comfort, measured as the average of five items assessing contact comfort. (*B*) Estimated society-level probabilities of individuals engaging in physical contact during greetings. Higher scores indicate greater comfort with these behaviors and a higher likelihood of physical contact during greetings. Points represent society-level predicted means (*A*) or probabilities (*B*) derived from the random intercepts of intercept-only multilevel models. Error bars indicate approximate 95% uncertainty intervals (±1.96 posterior SD) around the society-specific intercept estimates. Vertical dashed lines denote the grand mean (*A*) and the grand predicted probability (*B*) across societies.

Contact comfort varied little across societies: About 3% of the total variance was explained by between-society differences (ICC = 0.03). Contact greeting diverged more, with 7% of variance explained by societal differences (latent ICC = 0.07). For context, a different study of 21 psychological variables measured across 69 societies revealed ICCs ranging from 0.03 to 0.28, with a median of 0.10 (33). Hence, cross-societal variation in contact comfort is among the lowest within this wide array of variables, whereas variation in contact greeting falls near the middle of the distribution. Fig. 1 shows the society-level random intercepts for contact comfort and contact greeting, representing deviations from the grand mean.

### Is Infection-Risky Social Contact Positively Associated with Interpersonal Value Across Societies?

**Fig. 2.**
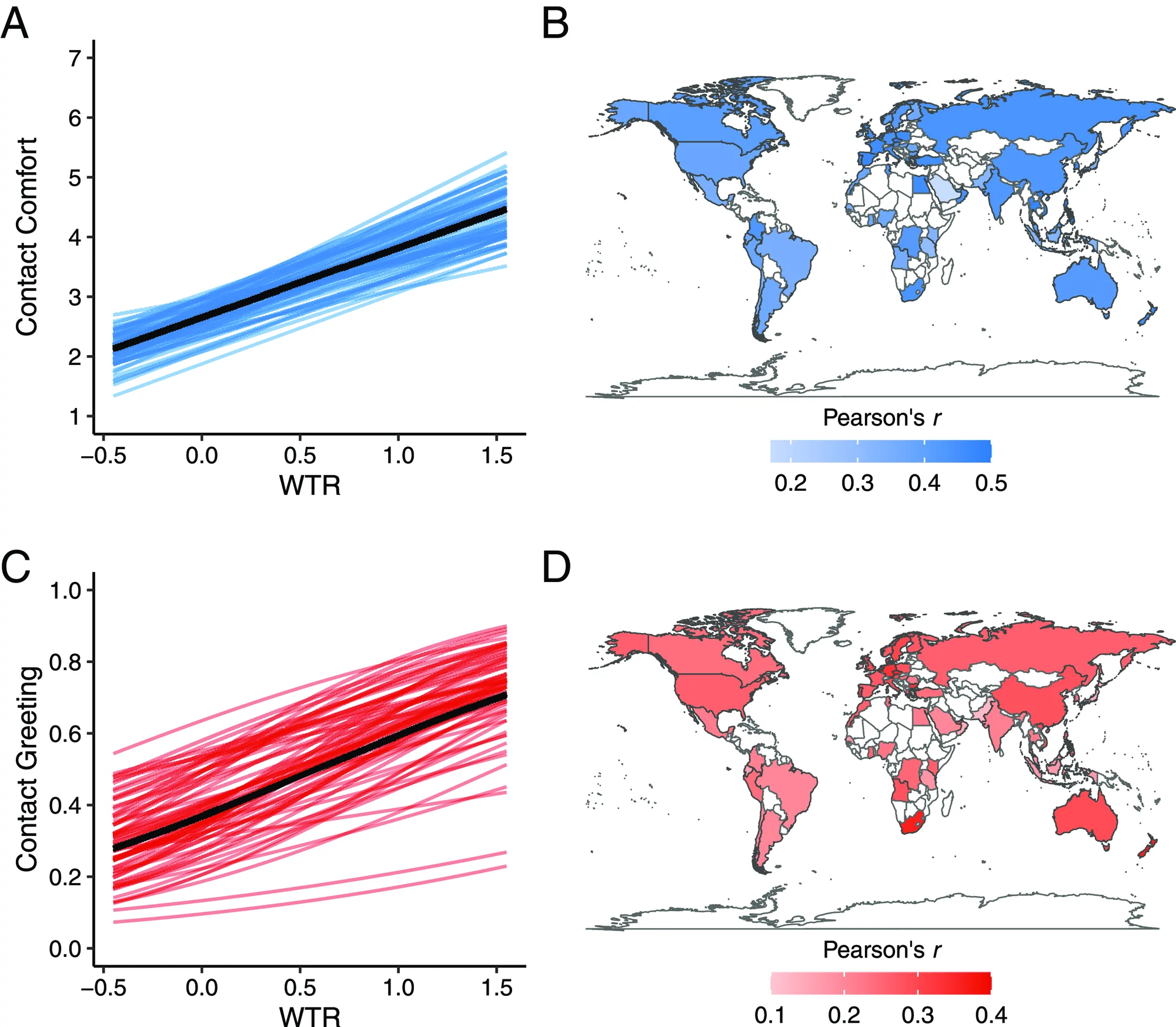
Interpersonal value positively associates with social contact across societies. (*A* and *B*) Associations between WTR and contact comfort (*A*) and a map of Pearson correlation coefficients for WTR and contact comfort (*B*). (*C* and *D*) Associations between WTR and contact greeting (*C*) and a map of Pearson correlation coefficients between WTR and contact greeting (*D*). Higher scores on the y-axis indicate greater comfort with these behaviors and a higher likelihood of physical contact during greetings. Greater WTR reflects placing greater value on others’ welfare relative to one’s own. Black lines show the overall effect across societies, and colored lines show effects within each society. Darker colors in the maps indicate stronger positive correlations.

As shown in the *Left* panel of Fig. 2, correlations between WTR and both contact comfort and contact greeting were positive within every society (all *P*s < 0.05; *SI Appendix*, Table S4). Meta-analyses of within-society correlations yielded average correlations of *r* = 0.43 (95% CI [0.42, 0.45], *P* < 0.001) for contact comfort and *r* = 0.25 (95% CI [0.24, 0.27], *P* < 0.001) for contact greeting, with limited between-society heterogeneity in both associations (contact comfort: *τ*^2^ = 0.003; contact greeting: *τ*^2^ = 0.002). Across societies, the associations between WTR and both outcomes remained statistically significant when accounting for relationship category (contact comfort: *b* = 0.76, 95% CI [0.71, 0.80], *P* < 0.001; contact greeting: OR = 1.84, 95% CI [1.73, 1.95], *P* < 0.001). Models accounting for societal nonindependence also showed positive associations (contact comfort: *b*_Median_ = 0.75, 95% CrI [0.71, 0.80]; contact greeting: OR_Median_ = 1.84, 95% CrI [1.73, 1.95]). Taken together, results indicated that higher interpersonal value consistently related to greater willingness to engage in infection-risky contact across societies, and that this relation remained even within relationship categories.

### Is Infection-Risk Social Contact Lower in Societies with Higher Pathogen Prevalence?

The association between contact comfort and pathogen prevalence was statistically significant (*b* = −0.12, 95% CI [−0.21, −0.03], *P* < 0.01, semipartial *R*^2^ = 0.006), though accounting for societal nonindependence yielded a 95% credible interval that included zero (*b*_Median_ = −0.08, 95% CrI [−0.20, 0.04]). The relation between contact greeting and pathogen prevalence was nonsignificant (OR = 1.18, 95% CI [0.94, 1.49], *P* = 0.168, semipartial *R*^2^ = 0.0005). In sum, results did not support a general association between pathogen prevalence and infection-risky contact.

### Is Infection-Risky Social Contact Related to Cultural Factors?

Relations between contact comfort and both relational mobility (*b* = 0.13, 95% CI [0.06, 0.21], *P* < 0.001, semipartial *R*^2^ = 0.03) and individualism (*b* = 0.10, 95% CI [0.04, 0.16], *P* < 0.001, semipartial *R*^2^ = 0.01) were statistically significant. Models accounting for societal nonindependence showed that the 95% credible interval for individualism included zero (*b*_Median_ = 0.06, 95% CrI [−0.02, 0.14]), whereas that for relational mobility excluded zero (*b*_Median_ = 0.11, 95% CrI [0.01, 0.21]). The relation between tightness–looseness and contact comfort was not statistically significant (*b* = −0.02, 95% CI [−0.08, 0.05], *P* = 0.638, semipartial *R*^2^ = 0.01).

For contact greeting, the relation with relational mobility was statistically significant (OR = 1.43, 95% CI [1.16, 1.77], *P* < 0.001, semipartial *R*^2^ = 0.03), but accounting for societal nonindependence yielded a 95% credible interval that included 1 (OR_Median_ = 1.21, 95% CrI [0.98, 1.48]). Relations between pathogen prevalence and both individualism (OR = 0.92, 95% CI [0.77, 1.11], *P* = 0.39, semipartial *R*^2^ = 0.005) and tightness–looseness (OR = 0.86, 95% CI [0.73, 1.03], *P* = 0.1, semipartial *R*^2^ = 0.005) were nonsignificant. In sum, as with pathogen prevalence, results did not support general associations between infection-risky contact and the cultural factors tested here, with only the relation between contact comfort and relational mobility being robust to controls for societal nonindependence.

### Is the Relation between Interpersonal Value and Infection-Risky Social Contact Moderated by Pathogen Prevalence and Cultural Factors?

**Fig. 3.**
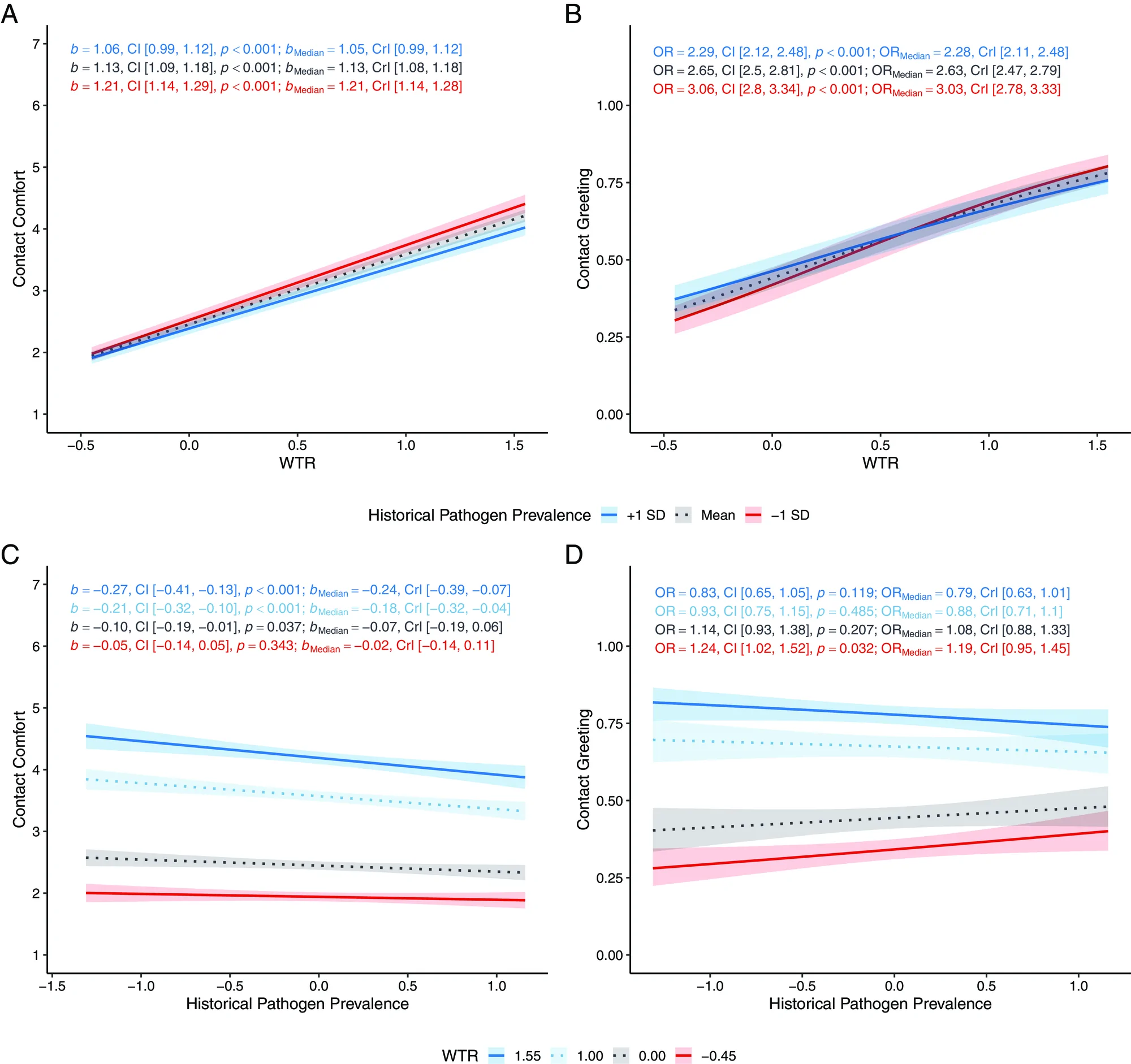
Simple slopes of the interactions between historical pathogen prevalence and WTR. (*A* and *B*) Predicted contact comfort (*A*) and contact greeting (*B*) as a function of WTR at three levels of historical pathogen prevalence (+1 SD, mean, and −1 SD). (*C* and *D*) Predicted contact comfort and contact greeting as a function of historical pathogen prevalence at four levels of WTR (1.55, 1, 0, and −0.45). WTR value at 1.55 indicates the highest value of WTR possible in the study; 1 indicates target welfare valued equally to one’s own; 0 indicates indifference to the target’s welfare; and −0.45 indicates the lowest value of WTR possible in the study. Lines represent frequentist estimates of simple slopes. Higher scores on the y-axis indicate greater comfort with these behaviors and a higher likelihood of physical contact during greetings. Text in the *Upper-Left* reports frequentist estimates of simple slopes, 95% CI (shown as shaded regions), and *P*-values, as well as Bayesian estimates of simple slopes and 95% credible intervals.

The interaction between pathogen prevalence and WTR was statistically significant for both contact comfort (*b* = −0.11, 95% CI [−0.18, −0.04], *P* = 0.002) and contact greeting (OR = 0.82, 95% CI [0.75, 0.89], *P* < 0.001), and these interactions were robust in models accounting for societal nonindependence (contact comfort: *b*_Median_ = −0.11, 95% CrI [−0.18, −0.04]; contact greeting: OR_Median_ = 0.82, 95% CrI [0.75, 0.90]). Contrary to predictions, simple slope analyses indicated that the association between WTR and contact comfort was slightly stronger in societies with lower pathogen prevalence (e.g., *b* = 1.21 at −1 SD) than in those with higher pathogen prevalence (e.g., *b* = 1.06 at +1 SD), as was the relation between WTR and contact greeting (OR = 3.06 versus OR = 2.29; details are shown in Fig. 3 *A* and *B*).

Simple slope analyses examining the relations between pathogen prevalence and contact comfort for high- versus low-value targets were also inconsistent with predictions. As shown in Fig. 3*C*, for relatively high-value targets (e.g., WTR at 1.55 and 1), contact comfort decreased as pathogen prevalence increased (*P*s < 0.001 and 95% credible intervals excluded zero); for low-value targets (e.g., WTR at −0.45 and 0), the relation between contact comfort and pathogen prevalence was not robust (*P*s > 0.05 or 95% credible intervals included zero). Results examining how pathogen prevalence related to contact comfort within relationship categories rather than WTR indicated a similar pattern (*SI Appendix*, section S3.5): the relation was negative within relatively high-value relationships (e.g., friends and acquaintances), but was not robust for low-value relationships (e.g., strangers or enemies). Simple slopes of pathogen prevalence on contact greeting were not robust when accounting for societal nonindependence at any of the tested levels of WTR (Fig. 3*D*) or within any relationship category (*SI Appendix*, section S3.5).

Cultural factors also moderated the association between interpersonal value and infection-risky contact. The interaction between WTR and individualism was statistically significant for both outcomes (contact comfort: *b* = 0.08, 95% CI [0.03, 0.13], *P* = 0.002; contact greeting: OR = 1.17, 95% CI [1.10, 1.24], *P* < 0.001), and remained robust in models accounting for societal nonindependence (contact comfort: *b*_Median_ = 0.08, 95% CrI [0.03, 0.14]; contact greeting: OR_Median_ = 1.16, 95% CrI [1.09, 1.25]). The interaction between WTR and tightness was statistically significant for contact comfort (*b* = −0.07, 95% CI [−0.13, −0.01], *P* = 0.019), but not for contact greeting (OR = 0.94, 95% CI [0.88, 1.00], *P* = 0.069). The former remained robust in models accounting for societal nonindependence (*b*_Median_ = −0.09, 95% CrI [−0.17, −0.01]). The interaction between WTR and relational mobility was statistically significant for both outcomes (contact comfort: *b* = 0.08, 95% CI [0.02, 0.14], *P* = 0.01; contact greeting: OR = 1.09, 95% CI [1.01, 1.18], *P* = 0.023), and both remained robust in models accounting for societal nonindependence (contact comfort: *b*_Median_ = 0.08, 95% CrI [0.01, 0.14]; contact greeting: OR_Median_ = 1.09, 95% CrI [1.01, 1.19]). Simple slope analyses showed that the positive association between WTR and infection-risky contact strengthened as individualism and relational mobility increased but weakened as tightness increased, though these differences in slope magnitude were modest (*SI Appendix*, Fig. S1). Similar to pathogen prevalence, cultural moderations on infection-risky contact were primarily observed among relatively high-value targets, whereas moderation for low-value targets was not robust (*SI Appendix*, Fig. S2). A similar pattern emerged when examining relationship categories instead of WTR, with robust association between cultural factors and infection-risky contact primarily observed for relatively high-value relationships (*SI Appendix*, section S3.6).

## Discussion

Across all cultures and times, humans must balance infection costs against the social benefits of interpersonal contact. Given the ambiguity of infection status in others, a well-designed system for regulating infection risks should rely not only on pathogen cues, but also on other types of social information (4, 5). In all 61 societies sampled, participants were more comfortable engaging in infection-risky contact with—and more likely to touch when greeting—social partners whose welfare they valued more. There was little variability in the magnitude of these relations. Such consistency across wide and diverse societies suggests a broadly shared psychology in which the interpersonal value of a social partner guides motivations to embrace or avoid infection-risky contact.

The cross-societal breadth of the study also afforded a test of parasite stress theory, which suggests that individuals in high pathogen prevalence societies should invest more in avoiding infection risks, especially those posed by targets lower in interpersonal value. Contrary to these predictions, results did not reveal a general relation between pathogen prevalence and infection-risky contact. However, they did indicate that pathogen prevalence is associated with lower contact comfort specifically with high-value targets (i.e., those with high WTR or those considered friends) rather than low-value ones (i.e., those with low WTR or those considered strangers or enemies). Although these results are not straightforwardly consistent with predictions from parasite stress theory, which emphasizes greater contact avoidance with strangers and outgroup members in high pathogen-prevalence societies, they are interpretable under the framework presented here. Given that contact with low-value others offers little social benefit, there is little to gain from contact that could transmit pathogens, even when the pathogen ecology is relatively benign. Because potential benefits are substantial for high-value targets, infection risk is more relevant for decisions to embrace or reject contact. Consequently, people accept that risk more readily where pathogen prevalence is low than where it is high.

Similar patterns emerged for the three cultural factors examined here—relational mobility, individualism-collectivism, and tightness–looseness. As with pathogen prevalence, each related to infection-risky social contact with high-value targets but not low-value ones. Each of these variables reflects a more flexible, less constrained social environment, with consequences for how relationships are formed, maintained, and dissolved (32, 34, 35). Cultural variation of this kind has been proposed to arise partially in response to ecological threats, including those posed by pathogens (28, 36, 37). Consistent with this view, evidence suggests that such cultural factors relate to the acceptance of antipathogen innovations (e.g., mask usage) and to the spread of some diseases (28, 30, 38). The current results suggest that they might also influence how people weigh the costs and benefits of social contact with high-value targets. When relationships are relatively more flexible and easier to dissolve, maintaining valued relationships demands greater investment. Given that infection-risky behaviors (e.g., saliva sharing) can indicate relational closeness (39, 40), tolerating the risks of such acts might be more beneficial in such societies. Similarly, when societies place fewer normative constraints on behaviors, as in relatively loose ones (27), individuals might feel freer to capitalize on the benefits of contact, such as showing the willingness to tolerate infection risks to express closeness to valued targets.

Overall, pathogen prevalence, individualism-collectivism, relational mobility, and tightness–looseness each related to infection-risky social contact for high-value targets but not low-value ones. The similarity in these patterns highlights the challenge of attributing cross-societal relations to any single factor, especially since they are correlated. Instead, the findings imply a common tradeoff: balancing the social benefits of contact with valued others against potential infection costs. And, indeed, despite modest differences in the slope of the relation between interpersonal value and contact variables across society-level variables, that relation was robust and positive in every society assessed, regardless of pathogen prevalence or cultural variables.

Notably, societal variation in contact greeting was larger than societal variation in contact comfort. Unlike the latter, which reflects internal motivations to engage in everyday actions that might incidentally carry pathogen transmission risks, greetings are ritualized actions that communicate information not only to the partner but also to observers. Such actions can signal cooperative intent, relative status, likelihood to aggress, and knowledge of and adherence to social norms, and their meaning can vary across societies (41–44). For example, one study found that bowing in Japan increases the perceived attractiveness of the bower, but does not do so in other East Asian cultures or other cultural contexts (45). The larger variation in greeting behaviors plausibly reflects additional differences in social signaling conventions rather than a greater difference between societies in the trade-off that regulates infection-risky social contact.

To ensure variation in WTR within societies and to prevent cross-societal variation in WTR emerging from cross-societal differences in the types of targets participants reported on, we randomly assigned participants to report on a target belonging to one of four folk relationship categories: their closest friend, an acquaintance, a stranger, or an enemy. While WTR accounted for variance in contact comfort and contact greeting even within relationship categories, relationship category also accounted for variance in both measures independent of WTR (*SI Appendix*, Tables S6 and S10). We offer two explanations for this outcome. First, the brief welfare-tradeoff task used here is an imperfect measure of WTR. Measurement error would attenuate the estimated effect of WTR and, consequently, inflate variance attributed uniquely to relationship category, which is correlated with true WTR. Second, relationship category and WTR each carry information the other does not. Category labels such as “friend” connote relational history and expected benefits beyond the welfare weighting that WTR captures, while WTR reflects willingness to sacrifice financially for reasons that a category label might not, such as formidability (7). These explanations are not mutually exclusive: Some of the unique effects of folk relationship category might result from measurement issues; others might reflect relational information that is distinct from WTR.

Hypotheses were generated from an adaptationist approach to cognitive science, which proposes that humans possess computational processes specialized for navigating recurrent adaptive problems, including those involving pathogen risk and cooperation (4, 5, 46). The present findings are consistent with this view, but they do not speak to the degree of specialization underlying the relation between interpersonal value and infection-risky contact. The same relation could potentially emerge from mechanisms that are not specialized for pathogen avoidance per se, but draw upon culturally transmitted knowledge, such as that grounded in germ theory. This possibility is difficult to exclude given that the sample included only individuals with internet access and 21st-century educational backgrounds. Two lines of evidence nonetheless speak against this possibility: people in the kinds of small-scale societies that humans evolved in appear to compute welfare tradeoffs much as people in societies with widespread internet access do (8), and people without formal education or knowledge of germ theory still possess a psychology of contamination (47, 48). Even so, future work could verify that relations observed in the populations sampled here are also apparent in small-scale societies less exposed to such culturally transmitted knowledge.

In conclusion, the present work demonstrates that the value people place on their social partners has implications beyond cooperation and other prosocial behaviors: It also relates to their willingness to expose themselves to potentially infectious microbes transmitted by another person. The relation between interpersonal value and infection-risky contact appears consistently across societies, though its magnitude shows some variation across social and ecological conditions. These findings reveal a potentially universal aspect of human psychology whose expression bends to local contexts.

## Materials and Methods

This project was approved by the Scientific and Ethical Review Board (VCWE) of the Faculty of Behavioural and Movement Sciences, Vrije Universiteit Amsterdam. Participants completed an online survey on interpersonal value and willingness to engage in infection-risky social contact, and all provided informed consent at the beginning of the survey. After providing demographic information, participants were randomly assigned to imagine one of four types of social targets: their closest friend, an acquaintance, a stranger, or a person they disliked intensely. Further instructions on target assignment are provided in *SI Appendix*. The target was referred to as Person X in subsequent measures.

### Interpersonal Value.

Interpersonal value was assessed using a decision-making task that estimated participants’ WTR toward Person X (8). On each trial, participants chose between a self-benefiting option (the participant received money and the target received nothing) and a target-benefiting option (the target received money and the participant received nothing). Across trials, the amount benefiting the participant varied, while the amount benefiting the target remained fixed. WTR was estimated as the point at which participants switched their preference from the self-benefiting option to the target-benefiting option, reflecting the relative value they placed on the target’s welfare compared to their own. Details of the task procedure and calculation of WTR are reported in *SI Appendix*. Greater WTR reflects placing greater value on others’ welfare relative to one’s own.

### Contact Comfort.

Participants rated their comfort with contact with Person X across five infection-risky behaviors on a 7-point Likert scale (from 1 = very uncomfortable to 7 = very comfortable). These behaviors included, for example, “sitting close enough to Person X that your legs are touching,” “standing close enough to Person X that his/her saliva might splash onto your face,” and “drinking from the same water bottle as Person X.” Evidence supporting the reliability and validity of this measure is provided in *SI Appendix*. Higher scores indicate greater comfort with these behaviors.

### Contact Greeting.

Participants also reported whether they would physically touch Person X during a typical greeting (e.g., handshaking, hugging, or kissing; coded as 0 = No and 1 = Yes). Higher value indicates a higher likelihood of physical contact during greetings.

### Societal-Level Factors.

Societal-level factors—including historical pathogen prevalence of nine diseases (e.g., tuberculosis) (49) and cultural indices of individualism-collectivism (32), tightness–looseness (27, 28), and relational mobility (35)—were obtained from existing studies.

### Data Analysis.

We examined within-society associations between interpersonal value and both contact comfort and contact greeting by estimating Pearson correlations between WTR and both outcomes (reporting two-tailed *P*-values) for each society, and then assessing the heterogeneity of these associations across societies using meta-analyses. We tested the between-society variation in contact comfort and contact greeting using the intraclass correlation coefficients (ICCs) from intercept-only models with participants nested within societies. Then, using linear multilevel models, we analyzed the associations of contact comfort with both interpersonal value and societal-level factors. Contact greeting was analyzed analogously using multilevel logistic regression models. To account for nonindependence among societies, we fit Bayesian multilevel models with structured random effects in which the covariance was determined by a Gaussian process based on geographic locations and/or a linguistic similarity matrix (21). Further details regarding the methods and analyses are reported in *SI Appendix*.

## Supplementary Material

Appendix 01 (PDF)

## Data Availability

Hypotheses, methods, analysis plans, and anonymized self-report questionnaire data have been deposited in Open Science Framework (https://osf.io/326xu) (50). All data, code, and materials are available in the same repository.
