# Supplementary material for: Interpersonal value regulates infection-risky social contact across 61 societies
Source: Proc Natl Acad Sci U S A. 2026 Oct 15;123(42):e2615739123. doi: 10.1073/pnas.2615739123 (PMC13684243; doi:10.1073/pnas.2615739123)
Supplement: Supplementary file 1 [file pnas.2615739123.sapp.pdf]

PNAS

Supporting Information for
Interpersonal value regulates infection-risky social contact across 61 societies.

Hongyu Sun, Giuliana Spadaro, Sofia Pelica, Roza Kamiloğlu, Loes Abrahams, Joshua M. Ackerman, Savannah Adams, Laith Al-Shawaf, Alexandra Alvergne, Benjamin Amponsah, Njål Andersen, Jan Antfolk, Derya Atamtürk Duyar, Songshi Bai, María Teresa Barbato, Sergio Barbosa, Pat Barclay, D. Vaughn Becker, Beatriz B. de Souza, Paulo Sérgio Boggio, Robert Böhm, Watcharaporn Boonyasiriwat, Andrea S. Camperio Ciani, Ali Rıza Can, Trevor I. Case, Kai Qin Chan, Luis Sebastian Contreras-Huerta, Paul Conway, Coralie Chevallier, Éloïse Côté, Sam P. Dale, Florencio Delgado Espinoza, Daniela Dlouhá, Izzet Duyar, Christian T. Elbæk, Qiran Fan, Ana Maria Fernandez, Diogo C. S. Ferreira, Daniel M.T. Fessler, Pablo Ezequiel Flores-Kanter, Natalia Frankowska, Ana Luísa Freitas, Márta Fülöp, Marcello Gallucci, Zachary H. Garfield, Margarida V. Garrido, Ekmel Gecer, Macarena González-Portilla, Dmitry Grigoryev, Minh-Tri Ha, Brahim Hamdaoui, Sahar Nadeem Hamid, Lina S. Hansson, Jan Havlíček, Benjamin E. Hilbig, Ian Hohm, Szilárd Holka, Minsung Hong, Yutaka Horita, Hsin-Ping Hsu, Yoel Inbar, Keiko Ishii, Olivia M. James, Tomasz Jarmakowski, Tingting Ji, William Jiménez-Leal, Shanmukh Kamble, Šárka Kaňková, Sonya Karabeliova, Honnête-Nyandu Kasali, Jinseok P. Kim, Toko Kiyonari, John Manuel R. Kliatchko, Nils Köbis, Ferenc Kocsor, Tobias L. Kordsmeye, Tom R. Kupfer, Lorena Lázaro Cuesta, Christine Xiang Ru Leong, David M. G. Lewis, Debra Lieberman, Hongtao Liu, Hernán López-Morales, Claudio López-Calle, Ingunn Olea Lund, Fiorella Macchiavello, Olga Marchenko, Urszula M. Marcinkowska, Gabriela Mariana Marcu, Norbert Meskó, Suyash Mishra, Kaloyan Mitev, Asako Miura, Fredy S. Monge Rodríguez, Leticia Y. Morello, Bruna E. F. Mota, Damian R. Murray, Felix-kingsley Obialo, Oluyinka Ojedokun, Mats J. Olsson, Gonzalo Palomo-Vélez, Folco Panizza, Fernanda Naomi Pantaleão, Lars Penke, Daniel Penn, Andrea Perinetti, Gerasimos Prodromitis, Ádám Putz, Angelica Quiroga-Garza, Nagireddy Neelakanteswar Reddy, Marc Eric S. Reyes, Felipe Rojas-Thomas, Rachel Rothschild, Kaelyn Sabree, Fatima Zahra Sahli, Cécile Sarabian, André Sarmento, Ya Hui Michelle See, Theresia Julius Shavega, Zubaida Shebani, Agnes Sianipar, Divita Singh, Sajjad Sojoudi, Francisco Leonardo Soler-Anguiano, Anna Soveri, Richard J. Stevenson, Steve Stewart-Williams, Ferran Suay, Eunkoook M. Suh, Jean-Roger Syayipuma Kambere, Ohad Szepsenwol, Carmit T. Tadmor, Tair Tager-Shafrir, Elahe Tavakoli-Berg, Shruti Tewari, Arnaud Tognetti, Antonia Tsitseli, Ignacio Leandro Uriarte, Martijn Van Heel, Florian van Leeuwen, Alfio Ventura, Jin Wang, XT (XiaoTian) Wang, Joseph Watts, Junhui Wu, Su-Ling Yeh, Eric Kenson Yau, Victoria Wai Lan Yeung, Janis H. Zickfeld, Joshua M. Tybur

Joshua M. Tybur
Email: j.m.tybur@vu.nl

This PDF file includes:

- Supporting text
- Figures S1 to S7
- Tables S1 to S47
- SI References

Contents of Supporting Information

S1. Materials and methods 3
S1.1. Participants 3
S1.2. Procedure and measures 3
S1.2.2. Individual-level measures 4
S1.2.3. Society-level measures 6
S2. Analytic strategy 7
S2.1. Reliability, validity, and measurement invariance for contact comfort 7
S2.2. Within-society analyses and meta-analyses of infection-risky social contact 7
S2.3. Between-society analysis of infection-risky social contact and WTR 7
S2.4. Analyzing associations between infection-risky social contact and relationship category 8
S2.5. Control for geographic and cultural nonindependence 8
S2.6. Analyzing the association between infection-risky social contact and contemporary pathogen prevalence 8
S3. Supplementary results 8
S3.1. Reliability, validity, and measurement invariance for contact comfort 8
S3.2. Meta-analysis of within-society associations 8
S3.3. Variation in WTR across societies and relationship categories 9
S3.4. Variation in contact comfort and contact greeting across relationship categories 9
S3.5. Interactions between relationship category and historical pathogen prevalence on infection-risky contact 9
S3.6. Interactions between relationship category and cultural factors on infection-risky contact 10
S3.7. The association between infection-risky contact and WTR within relationship category 11
S3.8. The association between infection-risky contact and contemporary pathogen prevalence 11
Figures 13
Tables 18
SI References 65

S1. Materials and methods

S1.1. Participants

This project was approved by the Scientific and Ethical Review Board (VCWE) of the Faculty of Behavioural and Movement Sciences, Vrije Universiteit Amsterdam (approval VCWE-2023-023). All participants provided informed consent at the beginning of the survey.

Participants were recruited via two routes: (i) a panel research company (Cint) and (ii) university samples (mostly undergraduate students). As of June 27, 2025, a total of 50,594 panel participants and 32,553 university participants accessed the online survey. Of these, 47,564 participants provided complete responses. From this group, we excluded testing data (collaborators' testing responses in the university sample), underage respondents, and inattentive respondents (see below). After these exclusions, 42,756 participants from 61 societies (59% female, M_{age} = 32.92, SD_{age} = 14.62) were included in the analysis. Full demographic information is provided in Table S1.

We first excluded testing data. Then, following our pre-registered criteria, we excluded participants who (i) reported to be younger than 18 years old; (ii) did not pass at least one of two attention check questions (i.e., "To verify that you are a genuine participant, please select 7" and "To verify that you are a genuine participant, please select 10%"); (iii) selected the same response options (i.e., straight-lined pattern) on two instruments with reverse-coded items that only used for attention check and not included in data analysis: six items from the disease avoidance subscale of the Fundamental Social Motives Inventory (1) and sixteen items generated for this study assessing knowledge of germ theory); (iv) retained only the first response and excluded any subsequent responses from participants who submitted more than one response (identified by account ID or student ID); (v) selected "Yes" to the question "Do you want to withdraw your consent and delete your data?".

We pre-registered a plan to exclude participants who provided implausible responses to two open-ended questions regarding height and weight estimates of the imagined target in this study. We departed from this plan because (1) many participants wrote feedback at the end of the survey indicating that height and weight were difficult to estimate, especially for those picturing a stranger, and (2) participants provided data in many different formats that could all be feasible responses for the same height estimate (e.g., 6'1", 185, 1.85, and 73). Hence, we concluded that target height and weight estimates had poor validity for screening purposes.

S1.2. Procedure and measures

The study follows the sequence outlined below. Participants were first asked to imagine a target. Then, with that target in mind, they then: (1) indicated whether a typical greeting with the target would involve physical contact, (2) reported how comfortable they would feel engaging in multiple acts of indirect contact that risk some microbe transmission from the target to themselves, and (3) completed a welfare trade-off task. The details of these procedures are described below.

S1.2.1. Target assignment

Participants were randomly assigned to one of the following targets: (i) their closest friend, (ii) an acquaintance, (iii) a stranger (an unknown person from the same society), or (iv) a person they know personally and dislike intensely (for brevity, referred to as "enemy" below). This approach was used to facilitate variation in target interpersonal value within society and mitigate potential inconsistencies in the nature of targets pictured across societies. We balanced target sex by assigning participants to picture either a female (e.g., "their closest female friend") or a male (e.g., "a male acquaintance") target in each condition. Below are the descriptions provided to participants.

> *Closest friend*: Next, we will ask you to make some financial decisions concerning your closest male (female) friend. Please imagine a male (female) friend who is very close to you but isn't a family member or romantic partner. We will refer to this man

(woman) as Person X. Before making these decisions, we will ask you to answer a few questions about Person X. Please take a moment to think about Person X and your relationship with Person X.

Acquaintance: Next, we will ask you to make some financial decisions concerning a male (female) acquaintance of yours. Please imagine a male (female) acquaintance – someone whose name you know but you do not have a close relationship with. We will refer to this man (woman) as Person X. Before making these decisions, we will ask you to answer a few questions about Person X. Please take a moment to think about Person X and your relationship with Person X.

Stranger: Next, we will ask you to make some financial decisions concerning a man (woman) from [participant's own society, e.g., the US] whom you don't know and probably won't meet in the near future. We will refer to this man (woman) as Person X. Before making these decisions, we will ask you to answer a few questions about Person X. Please take a moment to think about Person X.

Enemy: Next, we will ask you to make some financial decisions concerning a man (woman) that you know personally and dislike intensely. We will refer to this man (woman) as Person X. Before making these decisions, we will ask you to answer a few questions about Person X. Please take a moment to think about Person X and your relationship with Person X.

To encourage participants to think about the target, we asked them the following questions:

- How long have you known Person X?
- About how tall is Person X? (In inches/cm)
- About how much does Person X weigh? (In pounds/kg)
- In the past month, how many days were you in the same room as Person X?
- In the past month, how many days did you have any contact with Person X? (Including in-person talking, phone calls, messages, social media, and other methods)

S1.2.2. Individual-level measures

Contact greeting. Participants reported whether they would typically physically contact the target during a greeting: "People often greet each other. Some greetings involve physical contact. Examples include handshakes, hugs, and kisses. Other greetings do not involve physical contact. Examples include just saying hello, using a hand gesture, or bowing. Imagine that you greeted Person X today. Would you physically touch Person X?". Participants who indicated yes were asked to select which body parts they would touch during the greeting, including back, legs, arms, hands, chest, cheeks, and lips (i.e., "Which of your body parts would touch Person X when you greet him/her?"). Responses were coded as 0 (no) and 1 (yes).

Contact comfort. Participants rated their comfort with five actions involving exposure to others' microbes on a 7-point scale ranging from 1 (very uncomfortable) to 7 (very comfortable):

- Sitting close enough to Person X that your legs are touching.
- Standing close enough to Person X that his/her saliva might splash onto your face.
- Using the same toilet seat that Person X used minutes earlier.
- Putting Person X's slippers on your feet.
- Drinking from the same water bottle as Person X.

The items above were developed based on a pilot study conducted in four countries, including the United States (*N* = 220), Portugal (*N* = 219), China (*N* = 364), and the Netherlands (*N* = 221). These five items were selected from an initial pool of 17 items administered in the pilot based on exploratory factor analysis and measurement invariance analysis. Further details are reported in the *item selection* document on OSF (https://osf.io/326xu/files/cvmnk).

Welfare trade-off ratio (WTR). Participants completed a 15-item welfare trade-off task previously used in research on culturally diverse populations, which provides an estimate of the ratio of how much an individual values another's welfare (e.g., money) relative to their own (2, 3). In this task, participants make a series of 15 hypothetical financial decisions, choosing between an other-benefiting option (i.e., the target gets money) and a self-benefiting option (i.e., the participant gets money). In each trial, the amounts for the other-benefiting option were fixed. The amounts for the self-benefiting option were different, and the sequence of the items was randomized. Details of each option were provided in the *codebook* document on the OSF: https://osf.io/326xu/files/fythp).

WTR was estimated using the following procedure. For participants who switched once from choosing the self-benefiting option to the other-benefiting option, the estimation of WTR followed two steps below: first, calculate the ratio of the amounts of self-benefiting and other-benefiting; and second, WTR was assigned to the point at which participants switched from consistently selecting self-benefiting to other-benefiting. If participants always chose the self-benefiting option, WTR was estimated as -.45; if they always chose the other-benefiting option, WTR was estimated as 1.55. If participants had more than one switch point, WTR was estimated using a consistency maximization method, and see further information for this method in the work by Delton and colleagues (2). In line with previous work, participants who reported more than two switch points in this task were treated as missing data (4).

To make the meaning of the monetary units comparable across societies, we set the amounts in other-benefiting options to the society's median per-day wage, and we calibrated the self-benefiting amounts accordingly. Full amounts for each society are provided in the *codebook* document on the OSF: https://osf.io/326xu/files/fythp). Below are all 15 items used in the U.S. sample as an example. Items are identical across societies, except for the monetary amounts.

> Which of the following would you choose?
> Option 1: You get $334, Person X gets $0.
> Option 2: You get $0, Person X gets $230.
>
> Which of the following would you choose?
> Option 1: You get $288, Person X gets $0.
> Option 2: You get $0, Person X gets $230.
>
> Which of the following would you choose?
> Option 1: You get $242, Person X gets $0.
> Option 2: You get $0, Person X gets $230.
>
> Which of the following would you choose?
> Option 1: You get $218, Person X gets $0.
> Option 2: You get $0, Person X gets $230.
>
> Which of the following would you choose?
> Option 1: You get $196, Person X gets $0.
> Option 2: You get $0, Person X gets $230.
>
> Which of the following would you choose?
> Option 1: You get $172, Person X gets $0.
> Option 2: You get $0, Person X gets $230
>
> Which of the following would you choose?
> Option 1: You get $150, Person X gets $0.
> Option 2: You get $0, Person X gets $230.
>
> Which of the following would you choose?
> Option 1: You get $127, Person X gets $0.

Option 2: You get $0, Person X gets $230.

Which of the following would you choose?
Option 1: You get $104, Person X gets $0.
Option 2: You get $0, Person X gets $230.

Which of the following would you choose?
Option 1: You get $80, Person X gets $0.
Option 2: You get $0, Person X gets $230.

Which of the following would you choose?
Option 1: You get $58, Person X gets $0.
Option 2: You get $0, Person X gets $230.

Which of the following would you choose?
Option 1: You get $34, Person X gets $0.
Option 2: You get $0, Person X gets $230.

Which of the following would you choose?
Option 1: You get $12, Person X gets $0.
Option 2: You get $0, Person X gets $230.

Which of the following would you choose?
Option 1: You get -$34 (you lose $34), Person X gets $0.
Option 2: You get $0, Person X gets $230.

Which of the following would you choose?
Option 1: You get -$80 (you lose $80), Person X gets $0.
Option 2: You get $0, Person X gets $230.

S1.2.3. Society-level measures

Historical pathogen prevalence. Murray and Schaller developed an index of historical pathogen prevalence, which we employed in our analysis (5). This index assesses the presence of nine diseases within 230 geopolitical regions across history based on epidemiological atlases, including leishmaniasis, schistosomes, trypanosomes, leprosy, malaria, typhus, filariae, dengue, and tuberculosis. Positive values indicate a higher-than-average disease prevalence; the greater the value, the higher the prevalence. Scores were available for all 61 societies in our dataset.

Cultural factors. We employed three cultural indices: individualism-collectivism (larger values indicate more individualism and less collectivism) (6), tightness-looseness (greater values indicate more tightness) (7, 8), and relational mobility (larger values indicate greater mobility in relationships) (9). Data for these indices were available for 58, 41, and 29 societies in our dataset, respectively.

Contemporary pathogen prevalence. We used two indices to represent contemporary pathogen prevalence. The first was the index developed by Fincher et al. (2008), which was constructed from contemporary epidemiological data obtained from the Global Infectious Diseases and Epidemiology Online Network (GIDEON). The index covers 22 infectious diseases across seven pathogen categories (i.e., leishmaniasis, trypanosomes, malaria, schistosomes, filariae, spirochetes, and leprosy). Each disease was coded on a three-point prevalence scale, and scores were summed to create a composite index of contemporary pathogen prevalence (referred to as the *contemporary pathogen prevalence index* below). The second index used WHO infectious-disease data, following prior parasite-stress research that has relied on similar indicators to capture contemporary pathogen burden (10). We used age-standardized mortality rates from infectious and parasitic diseases (per 100,000 population) from the WHO Mortality Database. Whereas earlier studies were based on a single year, we averaged the death rate

across all available years to reduce annual fluctuations (referred to as the *average death rate* below). We excluded post-2019 observations to minimize potential bias introduced by the COVID-19 pandemic. Data for these two indices were available for 55 and 45 societies in our dataset, respectively. The bivariate correlations of these two indices with the historical pathogen prevalence in the current sample were 0.68 and 0.44 (both ps < 0.001), respectively.

S2. Analytic strategy

S2.1. Reliability, validity, and measurement invariance for contact comfort

Reliability of contact comfort within each society was assessed using Cronbach's alpha. The convergent validity of the contact comfort measure was assessed using multilevel models that examined its associations with two pathogen-avoidance measures: pathogen disgust sensitivity (11) and the disease-avoidance subscale of the Fundamental Social Motives Inventory (1). Next, measurement invariance across the two data recruitment categories (a panel company and collaborating universities) was assessed by multi-group confirmatory factor analysis of contact comfort using the *lavaan* package in R (12–14).

S2.2. Within-society analyses and meta-analyses of infection-risky social contact

For each society, we first calculated the means (M) and standard deviations (SD) of contact comfort, contact greeting, and WTR. Next, we examined the bivariate correlations between WTR and both contact comfort (Pearson's r) and between WTR and contact greeting (Point-biserial r). To quantify the variation in these correlations across societies, we applied Fisher's z transformation to the correlation coefficients to approximate normality. We then performed a two-level meta-analysis of the society-specific correlations using restricted maximum likelihood (REML) via the R package *metafor* (15). The model estimated the weighted average correlation and the between-society heterogeneity (τ^2). For interpretation, the average correlation and its confidence interval were backtransformed from Fisher's z to Pearson's r.

S2.3. Between-society analysis of infection-risky social contact and WTR

To account for the nested structure of data, we employed multilevel modeling (16) and used the *lme4* (17) and the *lmerTest* (18) packages in R (version 4.5.0). To estimate the intra-class correlation coefficient (ICC), we fitted intercept-only multilevel models (null models) – specifying random intercepts for societies – for contact comfort, contact greeting, and WTR. We also assessed variation in WTR across relationship categories and the variance in WTR explained by relationship category.

For the main analysis for contact comfort, we first analyzed the association between contact comfort and WTR when accounting for relationship category. We controlled for sample (panel vs university), target sex, participant sex, and the interaction between target sex and participant sex. The decision to include a random slope for WTR was based on a comparison of model fit indices between models with and without the random slope, including the likelihood ratio test, the Bayesian information criterion (BIC), and the Akaike information criterion (AIC) (19). Specifically, the random slope was retained when the likelihood-ratio test for the nested models was significant ($p < 0.05$), which tests whether the added variance component differs from zero. AIC and BIC (smaller values preferred) were reported to assess parsimony and predictive adequacy.

Next, we fitted separate models to examine whether each level-2 predictor (i.e., historical pathogen prevalence, individualism–collectivism, tightness, and relational mobility) was associated with infection-risky contact. We accounted for relationship factors (i.e., WTR and relationship category) and controlled for the same variables as described above. The R package *PartR2* was used to estimate semi-partial R^2 (20).

We then tested the moderations of societal-level predictors on the association between infection-risky contact and WTR. We fitted separate models testing the interaction between WTR and each level-2 predictor, including historical pathogen prevalence and three cultural factors (i.e., individualism–collectivism, tightness, and relational mobility). All level-2 predictors were

standardized. We then tested the two-way interactions between each level-2 predictor and WTR. We also tested whether societal-level factors moderate the association between WTR and infection-risky contact within relationship category.

We analyzed contact greeting using the same process with generalized multilevel models. The R packages *interactions* (21) and *emmeans* (22) were used for simple slope analysis and Tukey's post hoc test.

S2.4. Analyzing associations between infection-risky social contact and relationship category

As a complementary analysis of interpersonal value, we examined whether closer relationships were associated with greater comfort with infection-risky contact and a higher likelihood of physical touching during greetings, both within and across societies. For the within-society analysis, we estimated descriptive means of both outcomes for each relationship category. For the across-society analysis, we first regressed both outcomes on dummy-coded relationship category, controlling for the same variables described above (sample source, participant sex, and target sex). We then fitted separate models testing the interaction between relationship category and each societal-level predictor (i.e., historical pathogen prevalence, individualism, tightness, and relational mobility).

S2.5. Control for geographic and cultural nonindependence

To test the robustness of results after controlling for societal non-independence, we fitted Bayesian multilevel models via the *brm* package (23). Following Claessens and colleagues (24), spatial non-independence was controlled by adding a Gaussian process over the latitude and longitude of the geographic center within each society. Cultural non-independence was accounted for by adding a random intercept for society with a covariance matrix based on a linguistic matrix, which was obtained from the work by Claessens and colleagues (24). These methods allow societies to covary according to geographic and/or linguistic similarities. For models examining contact comfort, both geographic and linguistic nonindependence were included. Due to convergence issues, we fitted separate models with geographic proximity or linguistic proximity for models examining contact greeting.

S2.6. Analyzing the association between infection-risky social contact and contemporary pathogen prevalence

To explore the influence of contemporary pathogen prevalence on infection-risky social contact, we also assessed (i) whether greater contemporary pathogen prevalence was associated with lower contact comfort and a lower likelihood of contact during greetings, and (ii) whether it moderated the associations of WTR and relationship category with infection-risky contact. The analyses followed the same analytical steps used for historical pathogen prevalence.

S3. Supplementary results

S3.1. Reliability, validity, and measurement invariance for contact comfort

The results suggested good reliability across all societies (alpha coefficients ranging from 0.81 to 0.90; see Table S2). Greater contact comfort was associated with both lower pathogen disgust sensitivity (b = -0.24, 95% CI [-0.26, -0.21], $p < 0.001$) and disease avoidance (b = -0.19, 95% CI [-0.21, -0.17], $p < 0.001$). These findings were consistent with theoretical predictions regarding pathogen-avoidance constructs, providing evidence for the convergent validity of the contact comfort measure. Contact comfort had acceptable levels of configural invariance (all CFI changes across models smaller than 0.01; Table S3).

S3.2. Meta-analysis of within-society associations

In both outcomes, a majority of the observed variance reflected true effect heterogeneity rather than sampling error (contact comfort: I^2 = 67.19%; contact greeting: I^2 = 61.75%). Full results of correlation coefficients for each society are in Table S4.

S3.3. Variation in WTR across societies and relationship categories

Between-society differences accounted for about 2% of the variation in WTR (ICC = 0.02), suggesting minimal variation in overall WTR (25, 26). As expected, WTR varied across the target's relationship category, $F(3, 35695) = 2093$, $p < 0.001$. WTR was highest for friends (M = 0.55), followed by acquaintances (M = 0.29), strangers (M = 0.18), and enemies (M = -0.05). All pairwise comparisons are statistically significant, all ps < 0.001. Relationship category accounted for approximately 15% of the variance in WTR (semi-partial $R^2 = 0.15$).

S3.4. Variation in contact comfort and contact greeting across relationship categories

Across societies, contact comfort and contact greeting varied across relationship categories, $F(3, 42317) = 3654.16$, $p < 0.001$, and $\chi^2(3) = 3937.47$, $p < 0.001$, respectively. Specifically, individuals reported greater comfort and a higher likelihood of physical contact during greetings with friends, followed by acquaintances, strangers, and enemies (all pairwise comparisons are statistically significant, ps < 0.001; Tables S8 and S12). Such a pattern was consistent within most societies (Fig.S3). Across all relationship categories, the hands are the most common area of contact (Fig.S4). The arms are also common areas of contact, particularly when greeting friends and acquaintances. In contrast, lips and legs are uncommon during greetings across all four relationship categories.

S3.5. Interactions between relationship category and historical pathogen prevalence on infection-risky contact

The interaction between relationship category and historical pathogen prevalence on contact comfort was statistically significant, $F(3, 51) = 3.61$, $p = 0.019$. Specifically, relative to friends, the negative association between historical pathogen prevalence and contact comfort was significantly attenuated for strangers (b = 0.20, 95% CI [0.07, 0.34], p = 0.003; Table S14) and enemies (b = 0.19, 95% CI [0.06, 0.32], p = 0.004), whereas no significant difference was observed between acquaintances (b = 0.10, 95% CI [−0.01, 0.21], p = 0.067) and friends. As displayed in Fig. S5A, simple slopes showed that the decline in contact comfort was strongest for friends. Similarly, contact comfort was greater for friends and acquaintances in societies with lower historical pathogen prevalence, whereas no differences were observed for strangers or enemies across levels of historical pathogen prevalence (Table S15). These findings are consistent with the results from Bayesian models with a control for societal non-independence. The simple slopes of historical pathogen prevalence were credibly negative for friends (b = −0.23, 95% HPD [−0.39, −0.07]) and acquaintances (b = −0.13, 95% HPD [−0.25, −0.001]), whereas the slopes for strangers (b = −0.02, 95% HPD [−0.14, 0.10]) and enemies (b = −0.04, 95% HPD [−0.17, 0.09]) were not credibly different from zero. Results of interaction effects and pairwise comparison of Bayesian models are reported in Tables S16 and S17, respectively.

The interaction between relationship category and historical pathogen prevalence on contact greeting was also statistically significant, $\chi^2(3) = 13.12$, $p = 0.004$. Relative to friends, the association between historical pathogen prevalence and contact greeting was significantly more positive for enemies (OR = 1.28, 95% CI [1.08, 1.50], p = 0.003), whereas neither acquaintances (OR = 1.10, 95% CI [0.99, 1.23], p = 0.086) nor strangers (OR = 1.05, 95% CI [0.90, 1.22], p = 0.545) differed significantly from friends (Table S18). Simple slopes showed that historical pathogen prevalence was positively associated with contact greeting for enemies, but was not significantly associated with greeting for friends, acquaintances, or strangers (Fig. S5B). Pairwise comparisons across levels of historical pathogen prevalence within each relationship category were not statistically significant, all ps > 0.05 (Table S19). In Bayesian models with a control for societal non-independence, the simple slopes were not credibly different from zero for any relationship category, including enemies (b = 0.19, 95% HPD [−0.02, 0.41]), as well as friends (b = −0.06, 95% HPD [−0.29, 0.19]), acquaintances (b = 0.04, 95% HPD [−0.20, 0.27]), and strangers (b = −0.01, 95% HPD [−0.22, 0.21]). Results of interaction effects and pairwise comparison of Bayesian models are reported in Tables S20 and S21, respectively.

Overall, these results indicate that historical pathogen prevalence is associated with infection-risky contact primarily in relatively high-value relationships, whereas in low-value relationships, pathogen prevalence showed unrobust associations.

S3.6. Interactions between relationship category and cultural factors on infection-risky contact

Cultural factors also moderated the association between relationship category and both contact comfort and contact greeting. For contact comfort, the interaction between relationship category and individualism was significant, $F(3, 48) = 6.13$, $p = 0.001$ (see details in Table S28). Specifically, relative to friends, the association was significantly attenuated for acquaintances ($b = -0.10$, 95% CI [−0.17, −0.03], p = 0.005), strangers ($b = -0.19$, 95% CI [−0.28, −0.10], $p < 0.001$), and enemies ($b = -0.17$, 95% CI [−0.25, −0.08], $p < 0.001$). Simple slope analysis showed that contact comfort was positively associated with individualism for friends and acquaintances but not for strangers or enemies (Fig. S6). Similarly, contact comfort was greater in more individualistic societies for friends and acquaintances, whereas no differences emerged for strangers or enemies (Table S29). The interaction between relationship category and tightness was also significant, $F(3, 43) = 6.31$, $p = 0.001$. Contact comfort was less negatively associated with tightness for acquaintances ($b = 0.09$, 95% CI [0.02, 0.16], $p = 0.011$) and enemies ($b = 0.16$, 95% CI [0.07, 0.25], $p < 0.001$), but not for strangers ($b = 0.10$, 95% CI [−0.01, 0.21], $p = 0.085$), relative to friends. However, none of the simple slopes were statistically significant (Fig. S6), and contact comfort did not differ across levels of tightness for any relationship category (Table S29). The interaction between relationship category and relational mobility was also significant, $F(3, 23) = 6.33$, $p = 0.003$. Relative to friends, the association was attenuated for acquaintances ($b = -0.17$, 95% CI [−0.26, −0.07], $p = 0.001$), strangers ($b = -0.25$, 95% CI [−0.38, −0.13], $p < 0.001$), and enemies ($b = -0.21$, 95% CI [−0.31, −0.11], $p < 0.001$). Simple slope analysis suggested that contact comfort was positively associated with relational mobility for friends, acquaintances, and enemies, but not for strangers (Fig. S6). Similarly, contact comfort was greater in societies with higher relational mobility for all relationship categories except strangers (Table S29). Results were largely consistent in Bayesian models with a control for societal non-independence, except that the simple slopes of individualism for acquaintances and of relational mobility for enemies were not credibly different from zero (Fig.S6; Tables S30 and S31).

For contact greeting, the interaction between relationship category and individualism was significant, $\chi^2(3) = 10.87$, $p = 0.012$ (see details in Table S34). Relative to friends, the association was attenuated for enemies (OR = 0.82, 95% CI [0.73, 0.93], $p = 0.001$), but not for acquaintances (OR = 0.93, 95% CI [0.86, 1.00], $p = 0.054$) or strangers (OR = 0.91, 95% CI [0.81, 1.02], $p = 0.094$). None of the simple slopes were statistically significant (Fig. S6), and contact greeting did not differ across levels of individualism for any relationship category (Table S35). The interaction between relationship category and tightness was also significant, $\chi^2(3) = 14.63$, $p = 0.002$ (see details in Table S34). As with individualism, only the association between tightness and contact greeting for enemies differed significantly from that for friends (OR = 1.23, 95% CI [1.09, 1.38], $p = 0.001$), whereas the associations for acquaintances (OR = 1.06, 95% CI [0.97, 1.16], $p = 0.207$) and strangers (OR = 1.07, 95% CI [0.95, 1.21], $p = 0.238$) did not differ significantly from that for friends. Simple slope analysis indicated that contact greeting was negatively associated with tightness for friends and strangers, but not for acquaintances or enemies (Fig. S6). No statistically significant differences emerged in pairwise comparisons across levels of tightness using Tukey adjustment, all ps > 0.05 (Table S35). The interaction between relationship category and relational mobility was likewise statistically significant, $\chi^2(3) = 15$, $p = 0.002$ (see details in Table S34). Relative to friends, the association was attenuated for strangers (OR = 0.82, 95% CI [0.72, 0.93], $p = 0.002$) and enemies (OR = 0.81, 95% CI [0.70, 0.95], $p = 0.010$), but not for acquaintances (OR = 1.02, 95% CI [0.91, 1.14], $p = 0.731$). Contact greeting was positively associated with relational mobility for all four relationship categories (Fig. S6) and was correspondingly more likely in societies with higher relational mobility across all relationship categories (Table S35). Results were largely consistent in Bayesian models with a control for societal non-independence, except that the simple slopes of tightness for friends and strangers, and of relational mobility for strangers and enemies, were no longer credibly different from zero (Fig.S6; Tables S36 and S37).

In sum, cultural factors primarily influenced the willingness to engage in infection-risky contact with relatively close relationship partners. Individualism was associated with greater contact comfort only toward friends, whereas relational mobility was associated with greater contact comfort and a higher likelihood of contact greetings with friends and acquaintances. Tightness did not show reliable associations with either outcome. Similar to historical pathogen prevalence, cultural factors showed little evidence of consistently shaping infection-risky contact with relatively low-value relationships.

S3.7. The association between infection-risky contact and WTR within relationship category

When accounting for relationship category, the interaction between historical pathogen prevalence and WTR was not statistically significant for contact comfort (b = -0.06, 95% CI [-0.11, 0.004], p = 0.07). This interaction was statistically significant for contact greeting (OR = 0.87, 95% CI [0.81, 0.94], p < 0.001), and it remained negative in Bayesian models accounting for societal non-independence (OR_{Median} = 0.87, 95% CrI [0.80, 0.94]). However, Johnson–Neyman analyses of frequentist models indicated that the simple effect of historical pathogen prevalence was statistically significant (p < 0.05) only when WTR is below -0.72 or above 3.15 – values outside the observed range of WTR (minimum: -0.45, maximum: 1.55; Fig.S7A). Consistent with this, the 95% credible intervals for all simple slopes at the tested levels of WTR included 1 in the Bayesian analyses.

The interaction between interpersonal value and individualism was statistically significant for contact greeting (OR = 1.11, 95% CI [1.05, 1.17], p < 0.001) and it remained positive in Bayesian models accounting for societal non-independence (OR_{Median} = 1.11, 95% CrI [1.05, 1.17]), though the simple slopes of individualism were statistically significant only at values of WTR below -1.09 or above 2.46, outside the observed range (Fig.S7B). The 95% credible intervals for all simple slopes in the Bayesian analyses also included 1.

Relational mobility moderated the association between WTR and contact comfort (b = 0.06, 95% CI [0.01, 0.11], p < 0.05), and it remained positive in Bayesian models accounting for societal non-independence (b_{Median} = 0.06, 95% CrI [0.01, 0.11]). Simple slopes of relational mobility were statistically significant across the observed range of WTR in the frequentist analyses. However, in the Bayesian analyses, the 95% credible interval for the simple slope at the lowest observed WTR included zero (Fig. S7C).

The interactions between tightness and WTR on both outcomes (contact comfort: b = -0.04, 95% CI [-0.08, 0.01], p = 0.152; contact greeting: OR = 0.97, 95% CI [0.93, 1.03], p = 0.356) were not statistically significant.

Overall, when accounting for relationship category, relational mobility consistently moderated the association between WTR and contact comfort, indicating that even within the same relationship category, people in societies with higher relational mobility were more comfortable engaging in infection-risky physical contact with individuals they valued more highly. By contrast, the moderating effects of historical pathogen prevalence and individualism reached statistical significance only outside the observed range of WTR.

S3.8. The association between infection-risky contact and contemporary pathogen prevalence

In Bayesian multilevel models with a control for societal non-independence, the associations of the contemporary pathogen prevalence index with contact comfort (b_{Median} = −0.02, 95% CrI [−0.10, 0.05]; Table S42) and contact greeting (OR_{Median} = 1.09, 95% CrI [0.96, 1.28]; Table S45) were not credibly different from zero.

The interaction between the contemporary pathogen prevalence index and WTR was credible for both contact comfort (b_{Median} = −0.07, 95% CrI [−0.12, −0.02]; Table S42) and contact greeting (OR_{Median} = 0.87, 95% CrI [0.82, 0.93]; Table S45). For contact comfort, simple slope analysis indicated that contemporary pathogen prevalence was credibly negatively associated with contact

comfort at higher levels of WTR (WTR at 1.55: b_{Median} = −0.13, 95% HPD [−0.24, −0.02]; WTR at 1: b_{Median} = −0.09, 95% HPD [−0.18, −0.004]), whereas the slopes at lower levels of WTR were not credibly different from zero. For contact greeting, the association was credibly positive at the lowest level of WTR (WTR at −0.45: OR_{Median} = 1.16, 95% HPD [1.05, 1.28]), whereas the slopes at the remaining levels were not credibly different from zero. By contrast, for both outcomes the positive association between WTR and contact was credible at all levels of contemporary pathogen prevalence but declined as prevalence increased (contact comfort: at −1 SD, b_{Median} = 1.20, 95% HPD [1.12, 1.28]; mean, b_{Median} = 1.13, 95% HPD [1.08, 1.19]; +1 SD, b_{Median} = 1.06, 95% HPD [0.99, 1.14]; contact greeting: at −1 SD, OR_{Median} = 3.05, 95% HPD [2.80, 3.38]; mean, OR_{Median} = 2.67, 95% HPD [2.51, 2.85]; +1 SD, OR_{Median} = 2.33, 95% HPD [2.15, 2.54]).

The interaction between the contemporary pathogen prevalence index and relationship category was credible for contact comfort (Table S42): relative to friends, the association was credibly less negative for acquaintances (b_{Median} = 0.09, 95% CrI [0.01, 0.18], pd = 98.5%), strangers (b_{Median} = 0.13, 95% CrI [0.03, 0.24], pd = 99.3%), and enemies (b_{Median} = 0.14, 95% CrI [0.04, 0.23], pd = 99.6%). Simple slope analysis showed that contemporary pathogen prevalence was credibly negatively associated with contact comfort for friends (b_{Median} = −0.13, 95% HPD [−0.24, −0.02]) but not for the other relationship categories, and contact comfort was credibly greater in societies with lower contemporary pathogen prevalence for friends only (Table S44). For contact greeting, relative to friends, the association was credibly more positive for acquaintances (OR_{Median} = 1.13, 95% CrI [1.03, 1.22], pd = 99.6%; Table S45), whereas the differences for strangers and enemies were not credible. None of the simple slopes of the contemporary pathogen prevalence index were credibly different from zero, and no credible differences emerged in the pairwise comparisons for any relationship category (Table S47).

All other estimates for the contemporary pathogen prevalence index were not credibly different from zero (Tables S42–S47). For average death rate, no credible associations with either contact comfort or contact greeting emerged in any model, whether as a main effect, in interaction with WTR, or in interaction with relationship category; none of the simple slopes or pairwise comparisons were credibly different from zero (Tables S43, S44, S46, and S47).

Overall, consistent with the historical measure of pathogen prevalence, these findings suggest that greater contemporary pathogen prevalence might be associated with reduced willingness to engage in infection-risky contact with high-value social partners. Yet these interpretations should be made with caution, as a parallel index of contemporary pathogen threat, average death rate, showed no credible associations with either outcome.

Figures

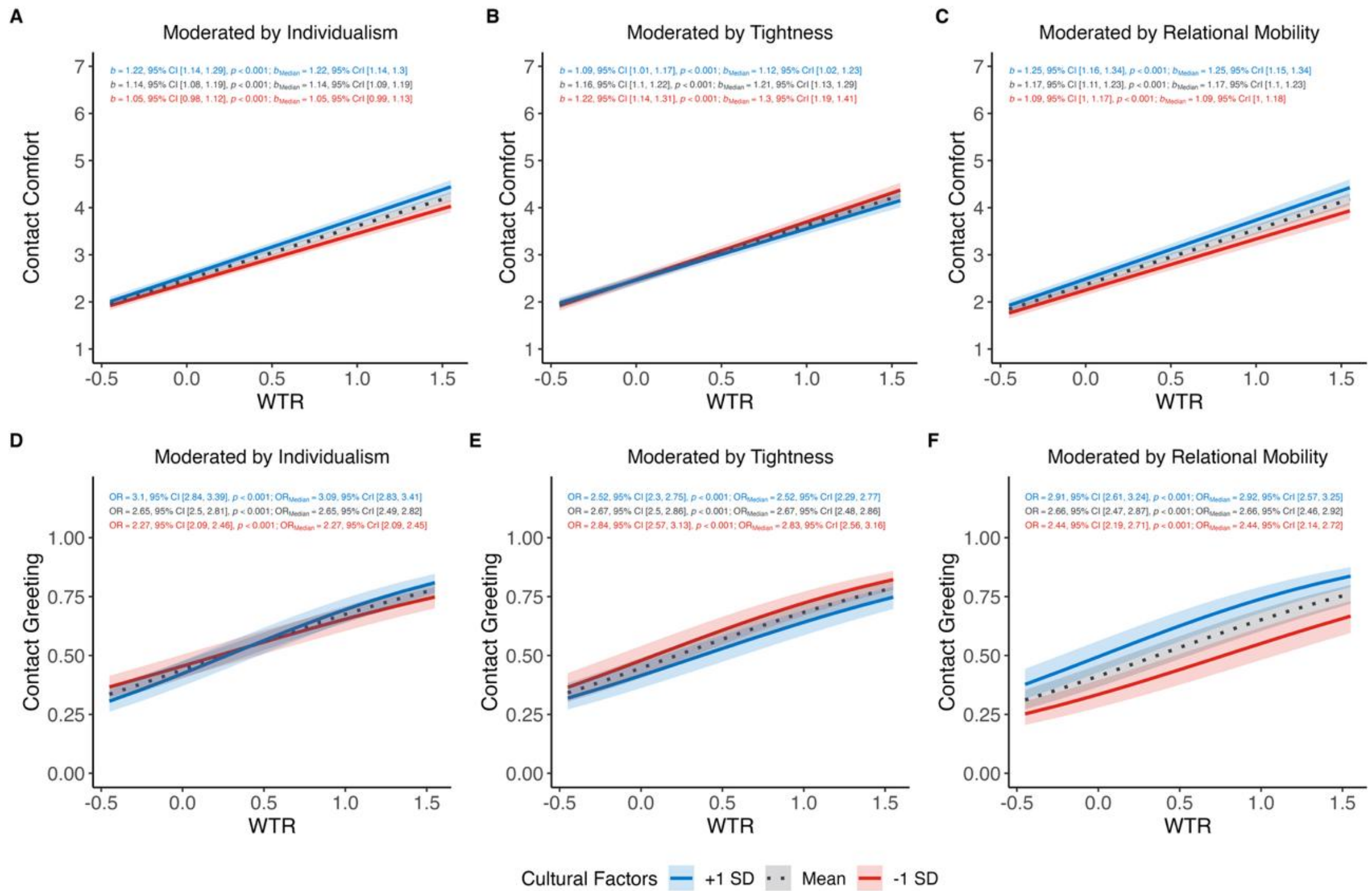

Fig.S1. Simple slopes of WTR on infection-risky contact at three levels of cultural factors (+1 SD, mean, and -1 SD)

(A – C) Predicted contact comfort as a function of WTR at three levels of individualism (A), tightness (B), and relational mobility (C). (D – F) Predicted contact greeting as a function of WTR at three levels of individualism (D), tightness (E), and relational mobility (F). Lines represent frequentist simple slopes. Text in the upper-left reports frequentist estimates of simple slopes, 95% confidence intervals (shown as shaded regions), and *p*-values, as well as Bayesian estimates of simple slopes and 95% credible intervals.

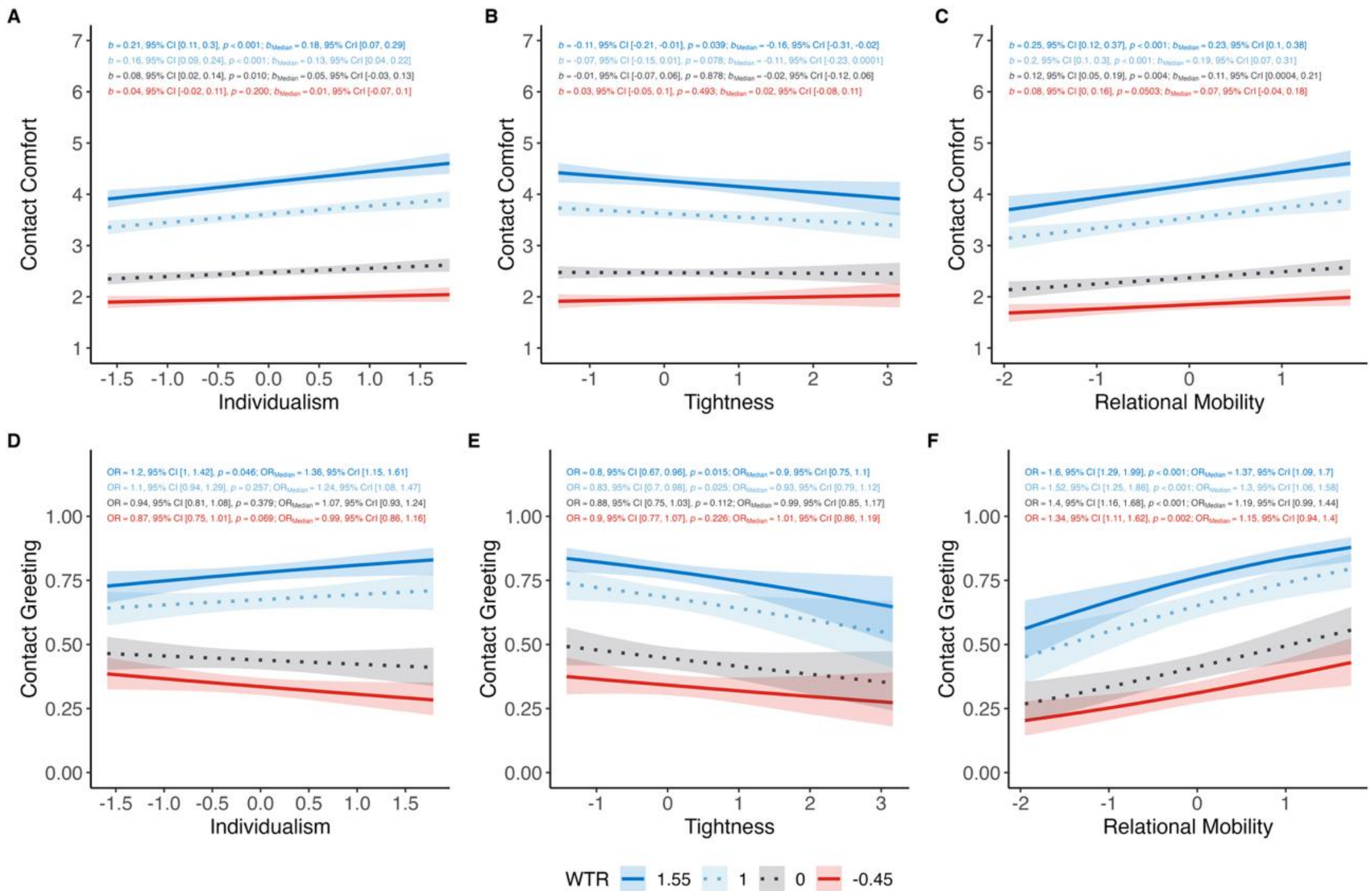

Fig.S2. Simple slopes of cultural factors on infection-risky contact at four levels of WTR (1.55, 1, 0, and −0.45)

(A – C) Predicted contact comfort as a function of individualism (A), tightness (B), and relational mobility at four levels of WTR. (D – F) Predicted contact greeting as a function of individualism (D), tightness (E), and relational mobility (F) at four levels of WTR. Lines represent simple slopes at four levels of WTR: 1.55 indicates the highest value of WTR possible in the study, 1 indicates target welfare valued equally to one's own, 0 indicates indifference to the target's welfare, and -0.45 indicates the lowest value of WTR possible in the study. Lines represent frequentist simple slopes. Text in the upper-left reports frequentist estimates of simple slopes, 95% confidence intervals (shown as shaded regions), and *p*-values, as well as Bayesian estimates of simple slopes and 95% credible intervals.

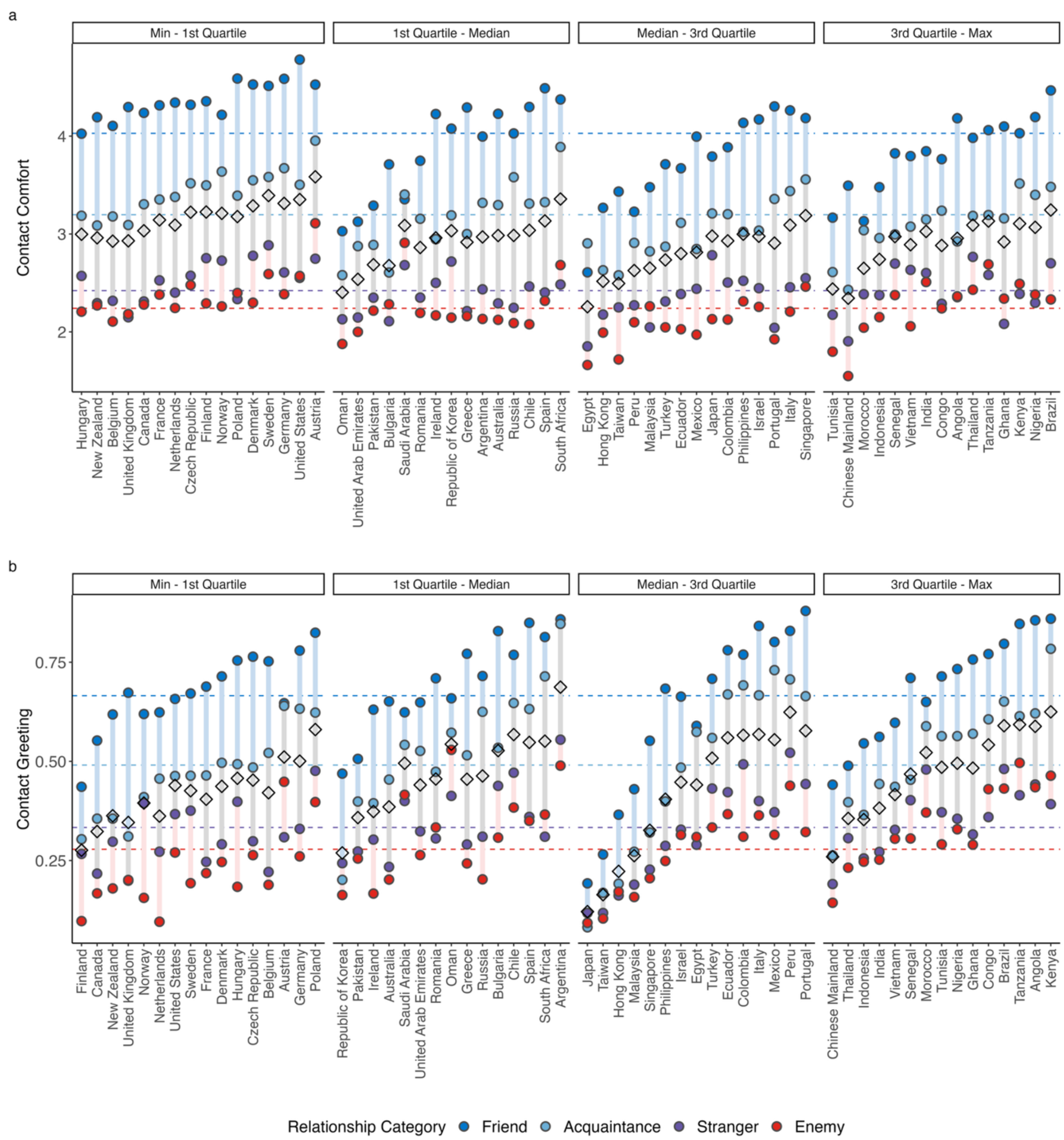

Fig.S3. Mean contact comfort and proportion of contact greeting by society

The panels indicate the ranges of historical pathogen prevalence. Points represent society-level means for each relationship category, diamonds represent means across relationship categories, and horizontal lines indicate means across societies for each relationship category.

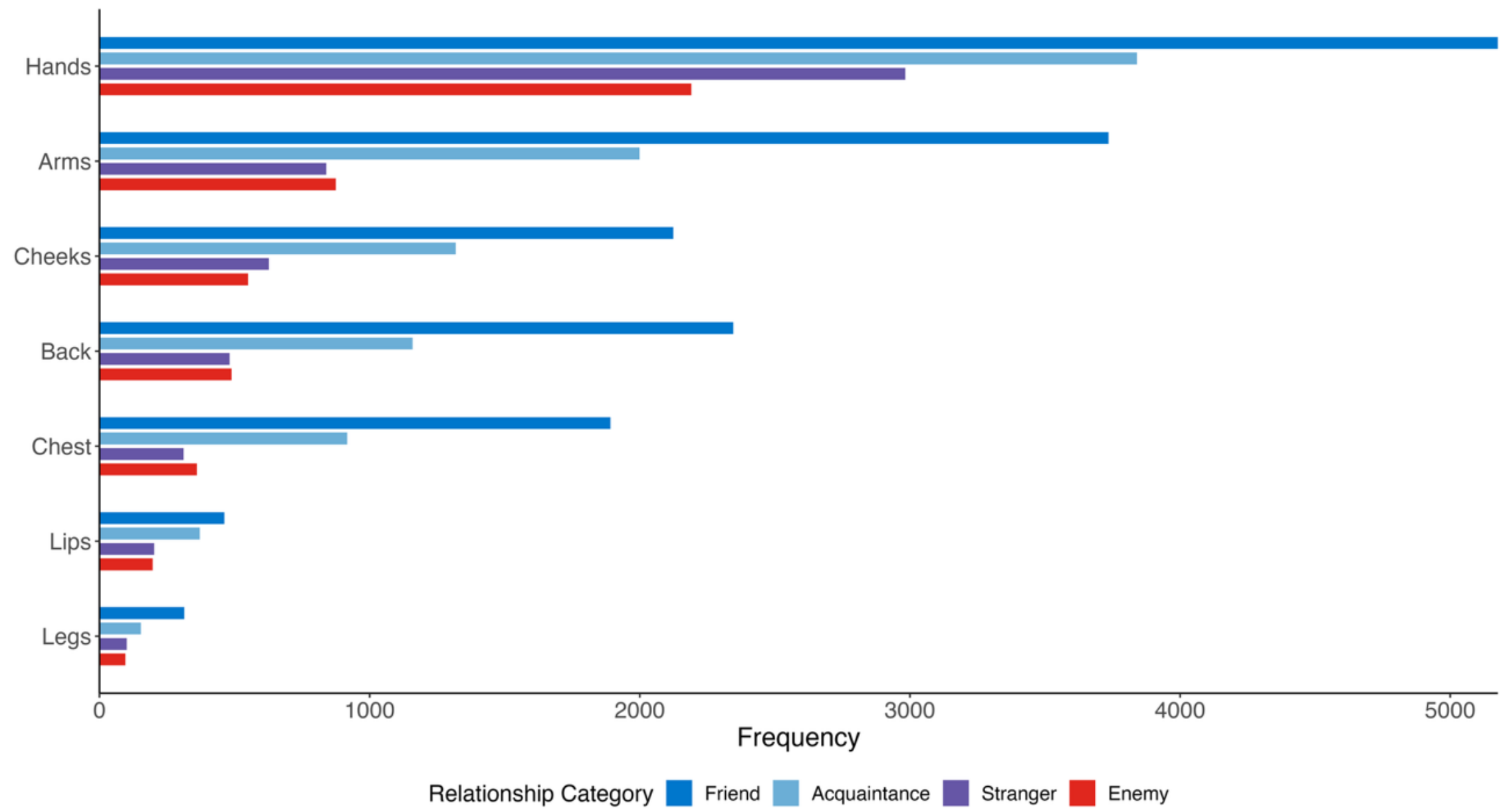

Fig.S4. Contact with different body parts during greetings

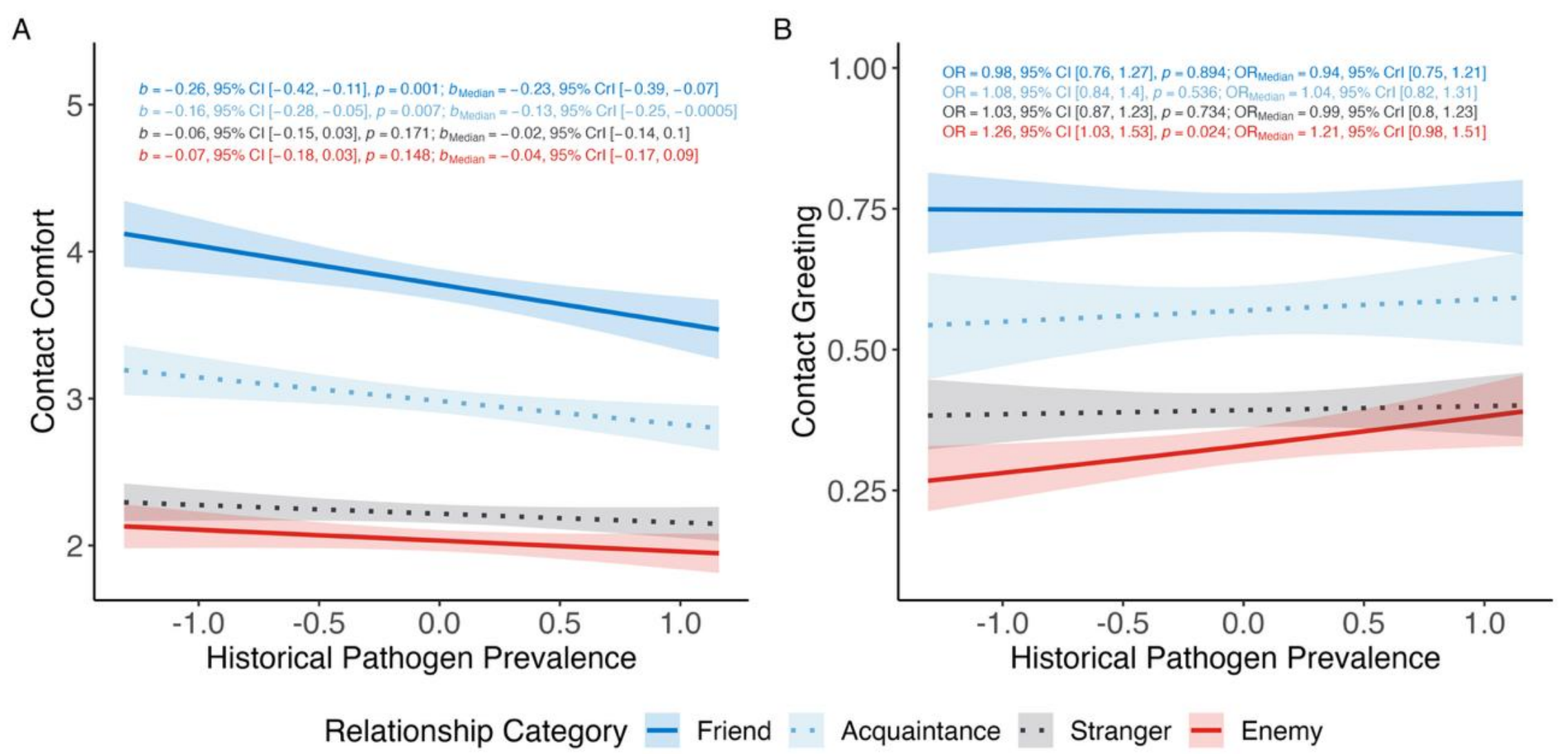

Fig.S5. Simple slopes of historical pathogen prevalence on contact comfort (A) and contact greeting (B) within each relationship category

Lines represent frequentist simple slopes within each relationship category. Text in the upper-left reports frequentist estimates of simple slopes, 95% confidence intervals (shown as shaded regions), and *p*-values, as well as Bayesian estimates of simple slopes and 95% credible intervals.

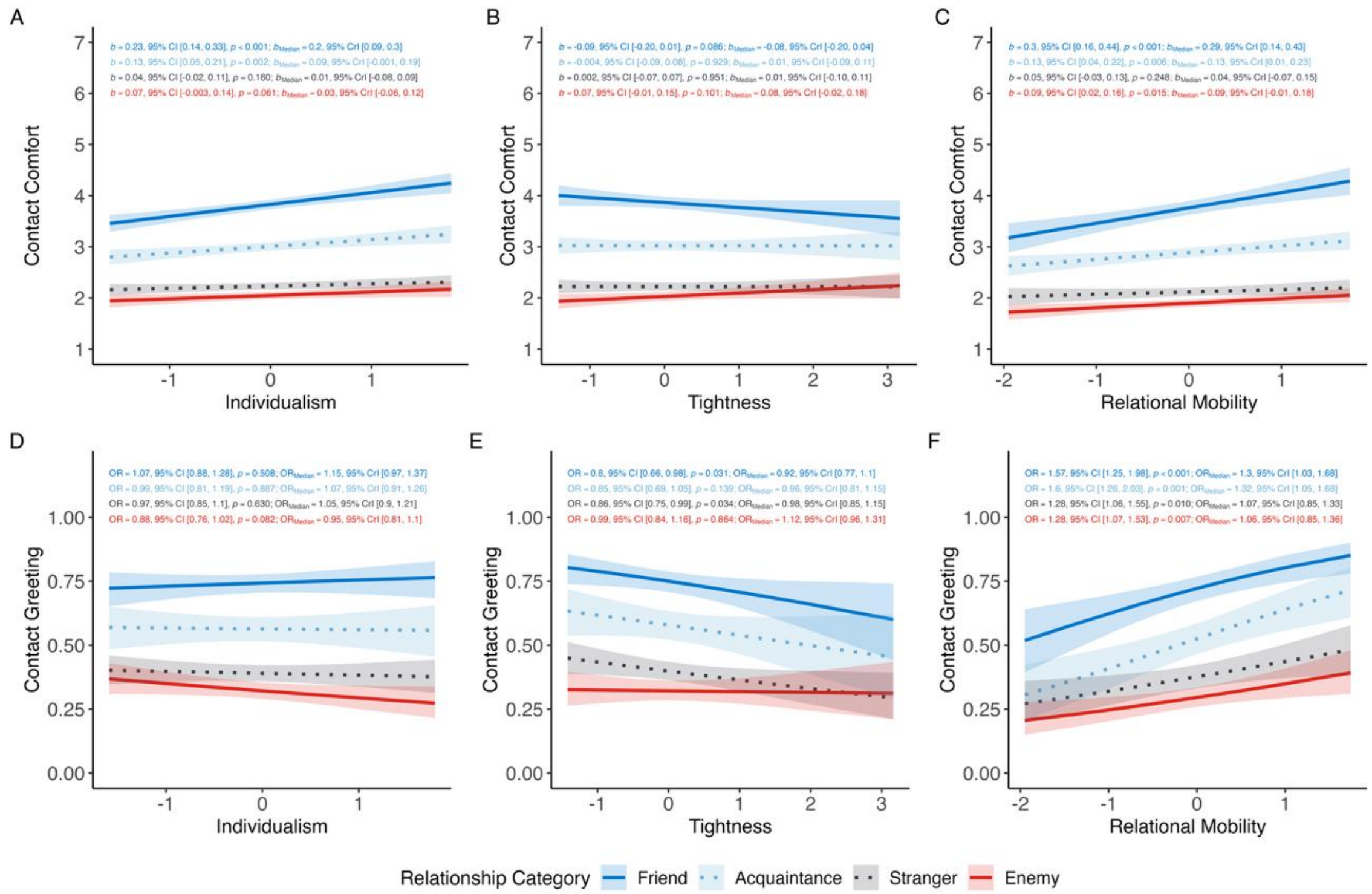

Fig.S6. Simple slopes of cultural factors on infection-risky contact within each relationship category

Lines represent frequentist simple slopes within each relationship category. Text in the upper-left reports frequentist estimates of simple slopes, 95% confidence intervals (shown as shaded regions), and *p*-values, as well as Bayesian estimates of simple slopes and 95% credible intervals.

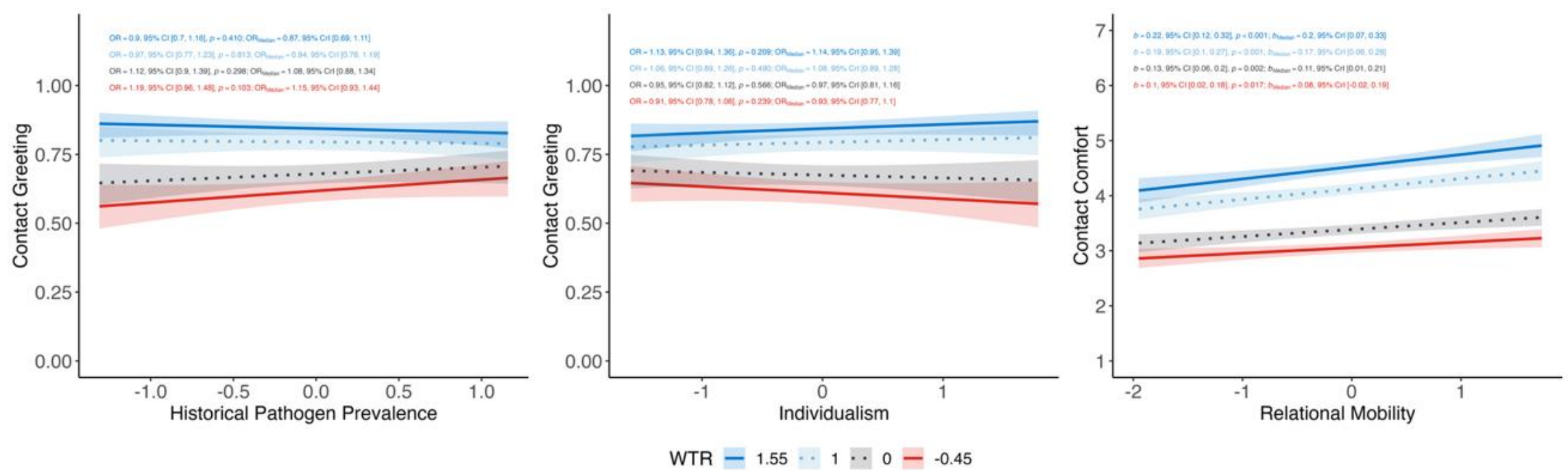

Fig. S7. Simple slopes of societal factors on infection-risky contact at four levels of WTR (1.55, 1, 0, and −0.45) when accounting for relationship category

(A and B) Predicted contact greeting as a function of historical pathogen prevalence (A) and individualism (B) at four levels of WTR. (C) Predicted contact comfort as a function of relational mobility at four levels of WTR. Lines represent frequentist simple slopes at four levels of WTR: 1.55 indicates the highest value of WTR possible in the study, 1 indicates target welfare valued equally to one's own, 0 indicates indifference to the target's welfare, and -0.45 indicates the lowest value of WTR possible in the study. Text in the upper-left reports frequentist estimates of simple slopes, 95% confidence intervals (shown as shaded regions), and *p*-values, as well as Bayesian estimates of simple slopes and 95% credible intervals.

Tables

Table S1. Demographic profile

Society	Panel				University				Total			
	N	Proportion female	M_{age}	SD_{age}	N	Proportion female	M_{age}	SD_{age}	N	Proportion female	M_{age}	SD_{age}
Angola	411	0.50	27.61	7.03	NA	NA	NA	NA	411	0.50	27.61	7.03
Argentina	444	0.51	43.59	11.57	117	0.82	28.35	9.96	561	0.57	40.41	12.84
Australia	400	0.50	55.16	17.19	460	0.85	22.44	6.96	860	0.69	37.66	20.73
Austria	441	0.52	31.79	15.67	144	0.76	21.90	6.62	585	0.58	29.36	14.62
Belgium	397	0.50	52.08	16.46	35	0.80	26.37	7.40	432	0.52	50.00	17.40
Brazil	402	0.50	42.76	13.37	769	0.75	22.20	4.65	1,171	0.67	29.26	13.07
Bulgaria	418	0.51	42.45	12.21	173	0.73	25.70	9.39	591	0.58	37.55	13.76
Canada	409	0.49	57.30	13.40	903	0.76	19.50	1.71	1,312	0.68	31.29	19.10
Chile	424	0.51	36.92	12.35	485	0.72	24.19	7.70	909	0.62	30.13	11.96
Chinese Mainland	401	0.50	34.37	7.19	511	0.71	21.57	2.20	912	0.62	27.20	8.12
Colombia	406	0.50	39.24	12.77	148	0.74	22.18	5.23	554	0.56	34.69	13.56
Congo	296	0.31	28.36	7.93	88	0.26	30.27	11.34	384	0.30	28.80	8.85
Czech Republic	403	0.49	30.97	13.35	213	0.78	23.78	5.50	616	0.59	28.48	11.77
Denmark	401	0.49	49.68	17.18	137	0.75	34.35	11.22	538	0.56	45.78	17.22
Ecuador	408	0.51	31.93	10.22	238	0.77	19.48	4.07	646	0.61	27.34	10.40
Egypt	400	0.50	34.65	11.88	NA	NA	NA	NA	400	0.50	34.65	11.88
Finland	398	0.51	45.07	15.36	210	0.77	27.22	8.57	608	0.60	38.90	15.87
France	402	0.49	50.75	16.02	412	0.77	23.00	8.06	814	0.63	36.71	18.76
Germany	398	0.50	34.25	17.47	376	0.80	31.12	15.37	774	0.65	32.73	16.55
Ghana	453	0.50	29.03	7.24	189	0.71	27.04	7.21	642	0.57	28.44	7.29
Greece	397	0.50	44.30	12.12	258	0.87	26.47	10.82	655	0.64	37.28	14.53
Hong Kong	396	0.49	36.86	11.10	172	0.64	23.12	6.59	568	0.54	32.70	11.79

Society	Panel				University				Total			
	N	Proportion female	M_{age}	SD_{age}	*N*	Proportion female	M_{age}	SD_{age}	*N*	Proportion female	M_{age}	SD_{age}
Hungary	416	0.49	39.04	13.06	1,016	0.76	27.67	10.45	1,432	0.68	30.97	12.39
India	393	0.51	33.61	11.11	542	0.73	21.39	3.94	935	0.64	26.53	9.86
Indonesia	425	0.50	34.16	9.89	316	0.80	26.54	6.80	741	0.63	30.91	9.48
Ireland	412	0.51	42.19	13.80	NA	NA	NA	NA	412	0.51	42.19	13.80
Israel	402	0.50	37.27	13.26	366	0.67	23.34	4.29	768	0.58	30.63	12.21
Italy	400	0.49	44.61	14.66	19	0.63	33.21	7.86	419	0.50	44.09	14.61
Japan	422	0.46	52.03	13.72	822	0.55	20.30	2.49	1,244	0.52	31.07	17.14
Kenya	424	0.50	32.70	9.78	NA	NA	NA	NA	424	0.50	32.70	9.78
Malaysia	402	0.51	35.06	10.88	111	0.79	20.16	1.71	513	0.57	31.84	11.45
Mexico	405	0.51	35.28	12.29	245	0.67	24.18	9.84	650	0.57	31.09	12.63
Morocco	443	0.49	32.23	10.70	143	0.48	36.45	11.47	586	0.48	33.26	11.03
Netherlands	400	0.50	54.16	18.50	243	0.85	20.05	1.94	643	0.63	41.27	22.09
New Zealand	406	0.51	45.60	18.69	220	0.84	20.44	4.58	626	0.62	36.76	19.44
Nigeria	424	0.50	32.71	10.01	187	0.50	23.39	8.18	611	0.50	29.86	10.41
Norway	413	0.49	48.21	17.46	58	0.47	24.16	5.21	471	0.48	45.25	18.25
Oman	401	0.50	31.45	8.52	231	0.63	21.25	3.54	632	0.55	27.72	8.64
Pakistan	421	0.49	32.22	10.17	202	0.85	21.15	3.02	623	0.61	28.63	9.98
Peru	405	0.50	36.14	12.18	235	0.66	26.91	8.88	640	0.56	32.75	11.94
Philippines	419	0.49	33.91	11.33	413	0.67	30.84	12.18	832	0.58	32.39	11.85
Poland	394	0.51	36.24	13.53	1,789	0.80	24.19	7.25	2,183	0.75	26.37	9.88
Portugal	411	0.49	39.65	11.81	168	0.88	22.85	7.91	579	0.60	34.78	13.24
Republic of Korea	409	0.49	44.78	13.45	190	0.66	21.15	2.06	599	0.54	37.29	15.69
Romania	396	0.50	43.45	13.45	260	0.87	23.83	6.82	656	0.65	35.68	14.82

Society	Panel				University				Total			
	N	Proportion female	M_{age}	SD_{age}	*N*	Proportion female	M_{age}	SD_{age}	*N*	Proportion female	M_{age}	SD_{age}
Russia	401	0.50	45.91	10.77	211	0.55	25.77	9.60	612	0.51	38.96	14.12
Saudi Arabia	402	0.49	32.58	8.96	NA	NA	NA	NA	402	0.49	32.58	8.96
Senegal	404	0.50	29.25	8.91	NA	NA	NA	NA	404	0.50	29.25	8.91
Singapore	401	0.49	38.13	12.55	431	0.64	25.17	8.25	832	0.57	31.42	12.37
South Africa	460	0.50	34.90	12.33	NA	NA	NA	NA	460	0.50	34.90	12.33
Spain	415	0.49	42.55	14.37	205	0.65	25.27	13.33	620	0.55	36.84	16.21
Sweden	395	0.50	46.72	16.71	210	0.78	40.89	12.38	605	0.60	44.70	15.58
Taiwan	398	0.50	38.08	11.58	335	0.60	25.39	10.10	733	0.55	32.28	12.62
Tanzania	409	0.51	28.21	5.71	85	0.54	35.39	11.81	494	0.51	29.44	7.62
Thailand	396	0.49	39.83	11.19	139	0.40	25.25	9.72	535	0.47	36.04	12.57
Tunisia	424	0.47	32.72	10.05	NA	NA	NA	NA	424	0.47	32.72	10.05
Turkey	494	0.48	35.70	10.97	590	0.67	30.49	11.46	1,084	0.58	32.86	11.53
United Arab Emirates	399	0.50	36.13	9.45	NA	NA	NA	NA	399	0.50	36.13	9.45
United Kingdom	432	0.50	53.69	15.65	427	0.81	25.89	11.09	859	0.65	39.87	19.43
United States	226	0.40	57.52	16.03	1,258	0.67	20.10	3.67	1,484	0.63	25.80	15.21
Vietnam	406	0.50	33.26	9.39	241	0.72	20.05	1.96	647	0.58	28.34	9.88
Total	24,810	0.49	39.21	14.83	17,946	0.72	24.24	8.67	42,756	0.59	32.93	14.62

Note. NA indicates no data. The "Total" row indicates overall statistics.

Table S2. Reliability (Cronbach's alpha) of contact comfort within each society

Society	Raw	Standardized	Society	Raw	Standardized
Angola	0.84	0.84	Mexico	0.88	0.88
Argentina	0.85	0.85	Morocco	0.86	0.86
Australia	0.88	0.89	Netherlands	0.88	0.89
Austria	0.87	0.87	New Zealand	0.87	0.88
Belgium	0.87	0.87	Nigeria	0.86	0.86
Brazil	0.86	0.86	Norway	0.86	0.86
Bulgaria	0.88	0.88	Oman	0.87	0.87
Canada	0.88	0.88	Pakistan	0.84	0.84
Chile	0.88	0.88	Peru	0.87	0.87
Chinese Mainland	0.90	0.90	Philippines	0.88	0.88
Colombia	0.88	0.88	Poland	0.88	0.88
Congo	0.81	0.81	Portugal	0.90	0.90
Czech Republic	0.88	0.88	Republic of Korea	0.89	0.89
Denmark	0.88	0.88	Romania	0.86	0.85
Ecuador	0.86	0.86	Russia	0.88	0.88
Egypt	0.88	0.88	Saudi Arabia	0.89	0.89
Finland	0.87	0.87	Senegal	0.83	0.83
France	0.86	0.86	Singapore	0.89	0.89
Germany	0.88	0.88	South Africa	0.88	0.88
Ghana	0.87	0.88	Spain	0.86	0.87
Greece	0.89	0.89	Sweden	0.85	0.85
Hong Kong	0.89	0.89	Taiwan	0.88	0.89
Hungary	0.87	0.87	Tanzania	0.84	0.84
India	0.84	0.84	Thailand	0.87	0.87
Indonesia	0.86	0.87	Tunisia	0.88	0.88
Ireland	0.87	0.87	Turkey	0.88	0.88
Israel	0.88	0.88	United Arab Emirates	0.89	0.89
Italy	0.88	0.88	United Kingdom	0.87	0.87
Japan	0.89	0.89	United States	0.87	0.87
Kenya	0.87	0.87	Vietnam	0.87	0.87
Malaysia	0.87	0.87	Total	0.87	0.87

Note. The "Total" row indicates overall reliability across societies.

Table S3. Fit indexes of measurement invariance of contact comfort

Model	χ^2	$\Delta\chi^2$	*df*	Δ*df*	CFI	ΔCFI	RMSEA	ΔRMSEA	SRMR	ΔSRMR
Model CI	3,113.23		10		0.969		0.12		0.026	
Model MI	3,223.26	110.03	14	4	0.968	0.001	0.104	0.016	0.031	0.005

Note. Model CI = Configural invariance model; Model MI = Metric invariance model. χ^2 = Chi-square, *df* = degree of freedom, $\Delta\chi^2$ = Chi-square difference, Δ*df* = change in degree of freedom, CFI = comparative fit index, ΔCFI = CFI change; RMSEA = root mean square error of approximation; ΔRMSEA = RMSEA change; SRMR = Standardized root mean square residuals; ΔSRMR = SRMR change.

Table S4. Society-specific relations between WTR and contact comfort and contact greeting

Society	WTR		Contact comfort		Contact greeting		r_{WTR_CC}	95% CI	Fisher's Z	r_{WTR_PG}	95% CI	Fisher's Z
	M	SD	*M*	SD	*M*	SD						
Angola	0.22	0.65	2.95	1.73	0.59	0.49	0.36^{***}	0.25 - 0.46	0.38	0.28^{***}	0.16 - 0.39	0.29
Argentina	0.20	0.57	2.99	1.59	0.69	0.46	0.37^{***}	0.28 - 0.45	0.39	0.20^{***}	0.11 - 0.29	0.20
Australia	0.27	0.59	3.02	1.63	0.39	0.49	0.42^{***}	0.36 - 0.47	0.45	0.29^{***}	0.22 - 0.35	0.30
Austria	0.39	0.53	3.60	1.68	0.51	0.50	0.44^{***}	0.36 - 0.51	0.47	0.28^{***}	0.20 - 0.36	0.29
Belgium	0.19	0.56	2.93	1.61	0.42	0.49	0.50^{***}	0.42 - 0.57	0.55	0.39^{***}	0.30 - 0.47	0.41
Brazil	0.19	0.51	3.24	1.67	0.59	0.49	0.35^{***}	0.29 - 0.40	0.37	0.20^{***}	0.14 - 0.26	0.20
Bulgaria	0.29	0.62	2.65	1.52	0.52	0.50	0.41^{***}	0.34 - 0.48	0.44	0.33^{***}	0.25 - 0.40	0.34
Canada	0.25	0.52	3.03	1.60	0.32	0.47	0.41^{***}	0.37 - 0.46	0.44	0.24^{***}	0.18 - 0.29	0.25
Chile	0.18	0.57	3.10	1.67	0.58	0.49	0.35^{***}	0.28 - 0.41	0.37	0.20^{***}	0.13 - 0.28	0.20
Chinese Mainland	0.18	0.47	2.37	1.37	0.26	0.44	0.43^{***}	0.38 - 0.49	0.46	0.28^{***}	0.22 - 0.34	0.29
Colombia	0.21	0.62	2.96	1.67	0.57	0.49	0.43^{***}	0.35 - 0.51	0.46	0.21^{***}	0.12 - 0.31	0.21
Congo	0.16	0.57	2.89	1.66	0.54	0.50	0.42^{***}	0.31 - 0.51	0.45	0.25^{***}	0.13 - 0.36	0.26
Czech Republic	0.36	0.54	3.26	1.50	0.46	0.50	0.49^{***}	0.42 - 0.55	0.54	0.35^{***}	0.27 - 0.42	0.37
Denmark	0.25	0.50	3.29	1.62	0.44	0.50	0.40^{***}	0.33 - 0.47	0.42	0.31^{***}	0.22 - 0.38	0.32
Ecuador	0.19	0.56	2.83	1.59	0.57	0.50	0.37^{***}	0.28 - 0.45	0.39	0.16^{***}	0.07 - 0.25	0.16
Egypt	0.29	0.60	2.25	1.57	0.44	0.50	0.46^{***}	0.38 - 0.54	0.50	0.22^{***}	0.12 - 0.32	0.22
Finland	0.24	0.47	3.25	1.45	0.28	0.45	0.37^{***}	0.29 - 0.44	0.39	0.27^{***}	0.19 - 0.34	0.28
France	0.26	0.53	3.11	1.60	0.40	0.49	0.46^{***}	0.40 - 0.51	0.50	0.27^{***}	0.20 - 0.33	0.28
Germany	0.32	0.53	3.32	1.63	0.50	0.50	0.44^{***}	0.38 - 0.50	0.47	0.33^{***}	0.26 - 0.40	0.34
Ghana	0.33	0.62	2.87	1.69	0.47	0.50	0.37^{***}	0.30 - 0.45	0.39	0.25^{***}	0.17 - 0.33	0.26
Greece	0.28	0.51	2.95	1.69	0.46	0.50	0.46^{***}	0.40 - 0.53	0.50	0.27^{***}	0.19 - 0.35	0.28
Hong Kong	0.12	0.48	2.52	1.33	0.22	0.42	0.36^{***}	0.28 - 0.43	0.38	0.21^{***}	0.12 - 0.29	0.21
Hungary	0.32	0.50	3.04	1.50	0.47	0.50	0.40^{***}	0.35 - 0.45	0.42	0.24^{***}	0.19 - 0.30	0.25
India	0.19	0.59	3.00	1.55	0.38	0.48	0.43^{***}	0.37 - 0.49	0.46	0.21^{***}	0.14 - 0.28	0.21
Indonesia	-0.07	0.55	2.77	1.45	0.36	0.48	0.33^{***}	0.26 - 0.40	0.34	0.17^{***}	0.10 - 0.25	0.17
Ireland	0.20	0.51	2.92	1.62	0.37	0.48	0.28^{***}	0.18 - 0.37	0.29	0.14^{**}	0.04 - 0.24	0.14
Israel	0.26	0.53	2.99	1.66	0.45	0.50	0.44^{***}	0.38 - 0.50	0.47	0.23^{***}	0.15 - 0.30	0.23
Italy	0.11	0.50	3.08	1.59	0.57	0.50	0.46^{***}	0.37 - 0.54	0.50	0.29^{***}	0.19 - 0.38	0.30
Japan	0.18	0.55	2.99	1.27	0.12	0.33	0.33^{***}	0.28 - 0.38	0.34	0.14^{***}	0.08 - 0.19	0.14
Kenya	0.25	0.63	3.15	1.72	0.64	0.48	0.33^{***}	0.23 - 0.43	0.34	0.26^{***}	0.15 - 0.36	0.27
Malaysia	0.06	0.56	2.59	1.45	0.25	0.43	0.41^{***}	0.33 - 0.48	0.44	0.17^{***}	0.08 - 0.26	0.17
Mexico	0.16	0.56	2.81	1.65	0.55	0.50	0.35^{***}	0.27 - 0.43	0.37	0.22^{***}	0.13 - 0.31	0.22
Morocco	0.25	0.62	2.63	1.67	0.52	0.50	0.38^{***}	0.31 - 0.46	0.40	0.26^{***}	0.17 - 0.34	0.27
Netherlands	0.28	0.50	3.13	1.58	0.37	0.48	0.47^{***}	0.40 - 0.53	0.51	0.29^{***}	0.22 - 0.36	0.30
New Zealand	0.23	0.58	2.94	1.52	0.36	0.48	0.46^{***}	0.39 - 0.52	0.50	0.36^{***}	0.28 - 0.43	0.38

Society	WTR		Contact comfort		Contact greeting		r_{WTR_CC}	95% CI	Fisher's Z	r_{WTR_PG}	95% CI	Fisher's Z
	M	SD	*M*	SD	*M*	SD						
Nigeria	0.31	0.56	3.09	1.67	0.50	0.50	0.37^{***}	0.28 - 0.44	0.39	0.23^{***}	0.14 - 0.31	0.23
Norway	0.25	0.51	3.20	1.57	0.39	0.49	0.40^{***}	0.32 - 0.48	0.42	0.27^{***}	0.18 - 0.36	0.28
Oman	0.28	0.60	2.44	1.57	0.55	0.50	0.45^{***}	0.38 - 0.52	0.49	0.14^{**}	0.05 - 0.22	0.14
Pakistan	0.30	0.70	2.69	1.51	0.36	0.48	0.32^{***}	0.24 - 0.40	0.33	0.12^{*}	0.03 - 0.21	0.12
Peru	0.13	0.59	2.61	1.49	0.62	0.49	0.38^{***}	0.30 - 0.46	0.40	0.24^{***}	0.15 - 0.32	0.25
Philippines	0.16	0.64	2.98	1.62	0.40	0.49	0.49^{***}	0.43 - 0.54	0.54	0.27^{***}	0.20 - 0.34	0.28
Poland	0.26	0.47	3.18	1.61	0.58	0.49	0.46^{***}	0.43 - 0.50	0.50	0.28^{***}	0.24 - 0.32	0.29
Portugal	0.33	0.58	2.89	1.73	0.57	0.50	0.44^{***}	0.37 - 0.51	0.47	0.31^{***}	0.23 - 0.38	0.32
Republic of Korea	0.11	0.42	3.08	1.50	0.28	0.45	0.46^{***}	0.39 - 0.52	0.50	0.24^{***}	0.16 - 0.31	0.25
Romania	0.27	0.58	2.88	1.58	0.46	0.50	0.37^{***}	0.29 - 0.44	0.39	0.22^{***}	0.14 - 0.30	0.22
Russia	0.14	0.54	2.96	1.60	0.46	0.50	0.44^{***}	0.37 - 0.51	0.47	0.26^{***}	0.18 - 0.34	0.27
Saudi Arabia	0.20	0.59	3.10	2.03	0.50	0.50	0.17^{**}	0.06 - 0.27	0.17	0.20^{***}	0.09 - 0.31	0.20
Senegal	0.22	0.64	2.98	1.68	0.47	0.50	0.35^{***}	0.25 - 0.44	0.37	0.18^{**}	0.07 - 0.29	0.18
Singapore	0.16	0.51	3.16	1.64	0.32	0.47	0.40^{***}	0.34 - 0.46	0.42	0.22^{***}	0.15 - 0.29	0.22
South Africa	0.27	0.59	3.39	1.84	0.56	0.50	0.44^{***}	0.35 - 0.51	0.47	0.36^{***}	0.27 - 0.44	0.38
Spain	0.22	0.51	3.15	1.57	0.55	0.50	0.47^{***}	0.41 - 0.54	0.51	0.26^{***}	0.18 - 0.34	0.27
Sweden	0.26	0.56	3.37	1.52	0.42	0.49	0.42^{***}	0.35 - 0.49	0.45	0.30^{***}	0.22 - 0.37	0.31
Taiwan	0.19	0.54	2.52	1.31	0.17	0.37	0.46^{***}	0.40 - 0.52	0.50	0.12^{**}	0.04 - 0.19	0.12
Tanzania	0.23	0.69	3.15	1.72	0.60	0.49	0.30^{***}	0.20 - 0.39	0.31	0.18^{***}	0.08 - 0.29	0.18
Thailand	0.24	0.63	3.10	1.61	0.36	0.48	0.48^{***}	0.41 - 0.55	0.52	0.22^{***}	0.13 - 0.31	0.22
Tunisia	0.22	0.63	2.40	1.59	0.47	0.50	0.43^{***}	0.34 - 0.51	0.46	0.25^{***}	0.15 - 0.34	0.26
Turkey	0.25	0.62	2.70	1.61	0.50	0.50	0.45^{***}	0.40 - 0.50	0.49	0.24^{***}	0.18 - 0.30	0.25
United Arab Emirates	0.23	0.57	2.56	1.71	0.45	0.50	0.39^{***}	0.29 - 0.48	0.41	0.30^{***}	0.19 - 0.39	0.31
United Kingdom	0.24	0.52	2.90	1.55	0.34	0.47	0.45^{***}	0.39 - 0.50	0.49	0.29^{***}	0.23 - 0.36	0.30
United States	0.28	0.51	3.34	1.60	0.44	0.50	0.38^{***}	0.33 - 0.42	0.40	0.26^{***}	0.21 - 0.31	0.27
Vietnam	0.30	0.59	2.88	1.57	0.41	0.49	0.49^{***}	0.42 - 0.55	0.54	0.29^{***}	0.20 - 0.36	0.30
Total	0.23	0.56	2.97	1.61	0.44	0.50	0.41^{***}	0.40 - 0.41	0.44	0.24^{***}	0.24 - 0.25	0.25

Note. r_{WTR_CC} and r_{WTR_PG} represent the Pearson correlation coefficients between WTR and contact comfort, and WTR and contact greeting, respectively. The "Total" row indicates the statistics calculated from the entire data across all societies. * $p < 0.05$, ** $p < 0.01$, *** $p < 0.001$.

Table S5. Intercept-only models for contact comfort, contact greeting, and WTR

	Contact comfort			Contact greeting			WTR		
**Predictors**	***b***	**95% CI**	***p***	**OR**	**95% CI**	***p***	***b***	**95% CI**	***p***
(Intercept)	2.95	2.88 – 3.02	<0.001	0.80	0.70 – 0.91	<0.001	0.23	0.21 – 0.25	<0.001
**Random Effects**									
σ^2	2.52			3.29			0.30		
τ_{00}	0.07 society			0.26 society			0.01 society		
ICC	0.03			0.07			0.02		
N	61 society			61 society			61 society		
Observations	42,756			42,756			35,750		
Marginal R^2 / Conditional R^2	0.00 / 0.03			0.00 / 0.07			0.00 / 0.02		

Note. ICC = Intraclass Correlation Coefficient, representing the proportion of variance in the outcome attributable to the society level. σ^2 is the within-society (residual) variance, and τ_{00} is the between-society variance. CI = Confidence interval. All models are intercept-only multilevel models with participants nested within societies. Marginal R^2 reflects the variance explained by fixed effects. Conditional R^2 reflects the variance explained by fixed and random effects.

Table S6. Multilevel models of WTR on contact comfort

	Model 1			Model 2			Model 3		
**Predictors**	***b***	**95% CI**	***p***	***b***	**95% CI**	***p***	***b***	**95% CI**	***p***
(Intercept)	3.81	3.73 – 3.88	<0.001	3.40	3.32 – 3.47	<0.001	3.39	3.32 – 3.47	<0.001
Relationship [Acquaintance]	-0.83	-0.87 – -0.79	<0.001	-0.67	-0.71 – -0.63	<0.001	-0.67	-0.71 – -0.63	<0.001
Relationship [Stranger]	-1.61	-1.64 – -1.57	<0.001	-1.38	-1.42 – -1.33	<0.001	-1.37	-1.41 – -1.33	<0.001
Relationship [Enemy]	-1.79	-1.83 – -1.75	<0.001	-1.40	-1.44 – -1.35	<0.001	-1.39	-1.43 – -1.35	<0.001
Target sex [Female]	0.52	0.48 – 0.56	<0.001	0.46	0.41 – 0.50	<0.001	0.46	0.41 – 0.50	<0.001
Participant sex [Female]	-0.21	-0.25 – -0.17	<0.001	-0.17	-0.21 – -0.13	<0.001	-0.17	-0.21 – -0.13	<0.001
Sample [University]	0.30	0.27 – 0.33	<0.001	0.28	0.25 – 0.32	<0.001	0.28	0.25 – 0.32	<0.001
Target sex [Female] × Participant sex [Female]	-0.13	-0.19 – -0.08	<0.001	-0.07	-0.12 – -0.01	0.01	-0.07	-0.13 – -0.01	0.009
WTR				0.75	0.72 – 0.78	<0.001	0.76	0.71 – 0.80	<0.001
**Random Effects**									
σ^2		1.94			1.73			1.72	
τ_{00}		0.07 society			0.06 society			0.06 society	
τ_{11}								0.02 society.WTR	
ρ_{01}								0.12 society	
ICC		0.03			0.03			0.04	
N		61 society			61 society			61 society	
Observations		42,395			35,461			35,461	
Marginal R^2 / Conditional R^2		0.23 / 0.25			0.30 / 0.32			0.30 / 0.32	

Note. τ_{11} indicates the variance of the random slope, and ρ_{01} indicates the correlation between the random intercept and slope.

Table S7. Comparison results of Model 2 and Model 3

	AIC	BIC	Log likelihood	Deviance	χ^2	*df*	*p*
Model 2	120,212	120,305	-60,095	120,190			
Model 3 (vs. Model 2)	120,169	120,279	-60,071	120,143	47.40	2	< 0.001

Table S8. Estimated marginal means of contact comfort by relationship categories and all Tukey-adjusted pairwise comparisons

	Category	*M*	SE	*df*	95% CI	
Estimated marginal means	Friend	4.08	0.04	76	4.00 – 4.15	
	Acquaintance	3.25	0.04	76	3.17 – 3.32	
	Stranger	2.47	0.04	76	2.40 – 2.54	
	Enemy	2.29	0.04	76	2.21 – 2.36	
	**Contrast (A – B)**	**Δ*M***	**SE**	***df***	***t***	***p***
Pairwise contrasts	Friend - Acquaintance	0.83	0.02	42,317	43.40	< 0.001
	Friend - Stranger	1.61	0.02	42,318	84.20	< 0.001
	Friend - Enemy	1.79	0.02	42,317	93.30	< 0.001
	Acquaintance - Stranger	0.78	0.02	42,317	40.50	< 0.001
	Acquaintance - Enemy	0.96	0.02	42,316	49.80	< 0.001
	Stranger - Enemy	0.18	0.02	42,317	9.60	< 0.001

Note. Pairwise comparisons were based on Model 1. *M* = estimated marginal mean; ΔM = mean difference (A − B); *SE* = standard error. Pairwise p-values were Tukey-adjusted; Estimated marginal means and contrasts are averaged over target sex, participant sex, and sample.

Table S9. Bayesian multilevel models of WTR on contact comfort controlling for societal nonindependence

Model	Parameter	b_{Median}	95% CrI	Posterior direction	R-hat	ESS
Model 4	(Intercept)	3.37	3.26 – 3.48	100%	1.000	2,838
	WTR	0.75	0.71 – 0.80	100%	1.000	2,881
	Relationship [Acquaintance]	-0.67	-0.71 – -0.63	100%	1.000	4,969
	Relationship [Stranger]	-1.37	-1.41 – -1.33	100%	0.999	4,564
	Relationship [Enemy]	-1.39	-1.43 – -1.35	100%	1.000	4,560
	Target sex [Female]	0.46	0.42 – 0.50	100%	1.000	4,076
	Participant sex [Female]	-0.17	-0.21 – -0.13	100%	1.001	4,485
	Sample [University]	0.28	0.25 – 0.32	100%	0.999	10,591
	Target sex [Female] × Participant sex [Female]	-0.07	-0.13 – -0.01	99.33%	1.000	3,786

Note. CrI represents credible intervals. Posterior direction represents the probability of direction; R-hat represents the convergence diagnostic. ESS represents the effective sample size. Model 4 controlled for societal nonindependence by adding both a Gaussian process over latitude and longitude [gp(latitude, longitude)] and a society-level random effect whose covariance is structured by linguistic proximity [(1 | gr(society, cov = linguistic proximity))].

Table S10. Multilevel models of WTR on contact greeting

	Model 5			Model 6			Model 7		
Predictors	**OR**	**95% CI**	***p***	**OR**	**95% CI**	***p***	**OR**	**95% CI**	***p***
(Intercept)	2.88	2.47 – 3.36	<0.001	2.13	1.81 – 2.50	<0.001	2.12	1.81 – 2.49	<0.001
Relationship [Acquaintance]	0.45	0.43 – 0.48	<0.001	0.50	0.47 – 0.53	<0.001	0.50	0.47 – 0.53	<0.001
Relationship [Stranger]	0.22	0.21 – 0.24	<0.001	0.26	0.24 – 0.28	<0.001	0.26	0.24 – 0.28	<0.001
Relationship [Enemy]	0.17	0.16 – 0.18	<0.001	0.22	0.21 – 0.24	<0.001	0.22	0.21 – 0.24	<0.001
Target sex [Female]	0.89	0.84 – 0.95	0.002	0.84	0.78 – 0.90	<0.001	0.84	0.78 – 0.90	<0.001
Participant sex [Female]	0.58	0.54 – 0.61	<0.001	0.59	0.55 – 0.63	<0.001	0.58	0.55 – 0.62	<0.001
Sample [University]	0.93	0.89 – 0.98	<0.01	0.92	0.87 – 0.97	0.003	0.92	0.87 – 0.97	0.003
Target sex [Female] × Participant sex [Female]	1.47	1.35 – 1.60	<0.001	1.52	1.38 – 1.67	<0.001	1.52	1.38 – 1.67	<0.001
WTR				1.82	1.74 – 1.91	<0.001	1.84	1.73 – 1.95	<0.001
**Random Effects**									
σ^2	3.29			3.29			3.29		
τ_{00}	0.32 society			0.34 society			0.33 society		
τ_{11}							0.02 society.WTR		
ρ_{01}							0.17 society		
ICC	0.09			0.09			0.09		
N	61 society			61 society			61 society		
Observations	42,381			35,448			35,448		
Marginal R^2 / Conditional R^2	0.13 / 0.21			0.15 / 0.23			0.15 / 0.23		

Table S11. Comparison results of Model 6 and Model 7

	**AIC**	**BIC**	**Log likelihood**	**Deviance**	**χ^2**	***df***	***p***
Model 6	41,606	41,690	-20,793	41,586			
Model 7 (vs. Model 6)	41,598	41,700	-20,787	41,574	11.30	2	< 0.01

Note. AIC and the likelihood-ratio test favored Model 7 (with the random slope for WTR), whereas BIC favored Model 6. Given that BIC's complexity penalty grows with sample size (27) and is therefore conservative at this sample size, we retained the random slope (Model 7).

Table S12. Estimated marginal means of contact greeting by relationship categories and all Tukey-adjusted pairwise comparisons

	Relationship	*M*	SE	95% CI	
Estimated marginal means	Friend	0.79	0.06	0.64 – 0.94	
	Acquaintance	0.00	0.06	-0.15 – 0.15	
	Stranger	-0.72	0.06	-0.86 – -0.57	
	Enemy	-0.99	0.06	-1.15 – -0.85	
	**Contrast (A /B)**	**Δ*M***	**SE**	***z***	***p***
Pairwise contrasts	Friend/Acquaintance	0.79	0.03	26.5	< 0.001
	Friend/Stranger	1.50	0.03	49.3	< 0.001
	Friend/Enemy	1.79	0.03	56.7	< 0.001
	Acquaintance/Stranger	0.72	0.03	24.2	< 0.001
	Acquaintance/Enemy	0.99	0.03	32.8	< 0.001
	Stranger/Enemy	0.28	0.03	9.2	< 0.001

Note. Pairwise comparisons were based on Model 5. Estimates are on a logit scale. *M* = estimated marginal mean; ΔM = mean difference (A − B); *SE* = standard error. Pairwise p-values were Tukey-adjusted; Estimated marginal means and contrasts are averaged over target sex, participant sex, and sample.

Table S13. Bayesian multilevel models of WTR on contact greeting controlling for societal nonindependence

Model	Parameter	LR_{Median}	95% CrI	Posterior direction	R-hat	ESS
Model 8	(Intercept)	0.73	0.53 – 0.94	100%	1.016	342
	Relationship [Acquaintance]	-0.69	-0.76 – -0.63	100%	0.999	3,554
	Relationship [Stranger]	-1.35	-1.42 – -1.28	100%	0.999	3,653
	Relationship [Enemy]	-1.50	-1.57 – -1.43	100%	0.999	3,660
	WTR	0.61	0.55 – 0.67	100%	1.000	3,104
	Target sex [Female]	-0.17	-0.24 – -0.10	100%	0.999	3,044
	Participant sex [Female]	-0.54	-0.61 – -0.47	100%	1.000	3,102
	Sample [University]	-0.08	-0.14 – -0.03	99.90%	1.000	4,621
	Target sex [Female] × Participant sex [Female]	0.42	0.32 – 0.51	100%	0.999	2,618
Model 9	(Intercept)	0.57	0.33 – 0.82	100%	1.008	329
	Relationship [Acquaintance]	-0.69	-0.76 – -0.63	100%	1.000	3,438
	Relationship [Stranger]	-1.35	-1.42 – -1.28	100%	1.000	3,283
	Relationship [Enemy]	-1.50	-1.57 – -1.43	100%	1.000	3,284
	WTR	0.61	0.55 – 0.67	100%	1.000	2,757
	Target sex [Female]	-0.17	-0.24 – -0.10	100%	1.001	2,185
	Participant sex [Female]	-0.54	-0.60 – -0.47	100%	1.001	2,219
	Sample [University]	-0.07	-0.13 – -0.02	99.60%	0.999	4,515
	Target sex [Female] × Participant sex [Female]	0.42	0.32 – 0.51	100%	1.002	1,874

Note. Estimates are on the logit scale. Credible intervals that include zero indicate that the effect is not clearly distinguishable from zero. Model 8 controlled for societal nonindependence through a Gaussian process over latitude and longitude [gp(latitude, longitude)]; Model 9 controlled for societal nonindependence through a society-level random effect whose covariance is structured by linguistic proximity [(1 | gr(society, cov = linguistic proximity))]. These two terms were entered in separate models because a model including both failed to converge.

Table S14. Multilevel models of historical pathogen prevalence and its interaction with WTR or relationship category on contact comfort

Predictors	Model 10			Model 11			Model 12		
	b	95% CI	*p*	*b*	95% CI	*p*	*b*	95% CI	*p*
(Intercept)	3.39	3.32 – 3.46	<0.001	2.45	2.38 – 2.51	<0.001	3.78	3.67 – 3.88	<0.001
Relationship [Acquaintance]	-0.67	-0.71 – -0.63	<0.001				-0.79	-0.87 – -0.72	<0.001
Relationship [Stranger]	-1.37	-1.41 – -1.33	<0.001				-1.56	-1.65 – -1.47	<0.001
Relationship [Enemy]	-1.39	-1.43 – -1.35	<0.001				-1.74	-1.83 – -1.66	<0.001
WTR	0.75	0.71 – 0.80	<0.001	1.12	1.08 – 1.17	<0.001			
Historical pathogen prevalence	-0.12	-0.21 – -0.03	0.007	-0.10	-0.19 – -0.01	0.033	-0.26	-0.41 – -0.11	0.001
Sample [University]	0.28	0.25 – 0.31	<0.001	0.26	0.23 – 0.29	<0.001	0.30	0.27 – 0.33	<0.001
Target sex [Female]	0.46	0.41 – 0.50	<0.001	0.41	0.36 – 0.46	<0.001	0.52	0.48 – 0.56	<0.001
Participant sex [Female]	-0.17	-0.21 – -0.13	<0.001	-0.16	-0.20 – -0.11	<0.001	-0.21	-0.25 – -0.17	<0.001
Target sex [Female] × Participant sex [Female]	-0.07	-0.13 – -0.01	0.014	-0.04	-0.10 – 0.02	0.224	-0.13	-0.18 – -0.08	<0.001
WTR × Historical pathogen prevalence				-0.11	-0.18 – -0.04	0.002			
Relationship [Acquaintance] × Historical pathogen prevalence							0.10	-0.01 – 0.21	0.067
Relationship [Stranger] × Historical pathogen prevalence							0.20	0.07 – 0.34	0.003
Relationship [Enemy] × Historical pathogen prevalence							0.19	0.06 – 0.32	0.004
**Random Effects**									
σ²	1.72			2.01			1.92		
τ_{00}	0.05 society			0.05 society			0.15 society		
τ_{11}	0.02 society.WTR			0.03 society.WTR			0.07 society.Acquaintance<br>0.11 society.Stranger<br>0.10 society.Enemy		
ρ_{01}	0.02 society			-0.01 society			-0.68<br>-0.86<br>-0.78		
ICC	0.03			0.03			0.04		
N	61 society			61 society			61 society		
Observations	35,448			35,448			42,381		
Marginal R^2 / Conditional R^2	0.30 / 0.33			0.19 / 0.22			0.23 / 0.26		

Table S15. Estimated marginal means of contact comfort by levels of historical pathogen prevalence and all Tukey-adjusted pairwise comparisons within relationship category

	Relationship	Levels of historical pathogen prevalence	*M*	*SE*	*df*	95% CI	
Estimated marginal means	Friend	-1 SD (-0.77)	4.25	0.08	58	4.09 – 4.41	
		Mean (-0.07)	4.06	0.05	59	3.96 – 4.17	
		+ 1 SD (0.64)	3.87	0.07	60	3.73 – 4.02	
	Acquaintance	-1 SD (-0.77)	3.38	0.06	57	3.26 – 3.50	
		Mean (-0.07)	3.26	0.04	59	3.18 – 3.34	
		+ 1 SD (0.64)	3.15	0.05	60	3.04 – 3.26	
	Stranger	-1 SD (-0.77)	2.53	0.04	56	2.44 – 2.62	
		Mean (-0.07)	2.49	0.03	59	2.43 – 2.55	
		+ 1 SD (0.64)	2.45	0.04	61	2.37 – 2.53	
	Enemy	-1 SD (-0.77)	2.36	0.05	57	2.25 – 2.46	
		Mean (-0.07)	2.31	0.03	59	2.24 – 2.38	
		+ 1 SD (0.64)	2.25	0.05	60	2.16 – 2.35	
	**Relationship**	**Contrast (A − B)**	**Δ*M***	***SE***	***df***	***t***	***p***
Pairwise contrasts	Friend	-1 SD (-0.77) − Mean (-0.07)	0.19	0.05	59	3.45	< 0.01
		-1 SD (-0.77) − + 1 SD (0.64)	0.37	0.11	59	3.45	< 0.01
		Mean (-0.07) − + 1 SD (0.64)	0.19	0.05	59	3.45	< 0.01
	Acquaintance	-1 SD (-0.77) − Mean (-0.07)	0.11	0.04	58	2.78	< 0.05
		-1 SD (-0.77) − + 1 SD (0.64)	0.23	0.08	58	2.78	< 0.05
		Mean (-0.07) − + 1 SD (0.64)	0.11	0.04	58	2.78	< 0.05
	Stranger	-1 SD (-0.77) − Mean (-0.07)	0.04	0.03	57	1.39	0.355
		-1 SD (-0.77) − + 1 SD (0.64)	0.08	0.06	57	1.39	0.355
		Mean (-0.07) − + 1 SD (0.64)	0.04	0.03	57	1.39	0.355
	Enemy	-1 SD (-0.77) − Mean (-0.07)	0.05	0.04	58	1.47	0.314
		-1 SD (-0.77) − + 1 SD (0.64)	0.11	0.07	58	1.47	0.314
		Mean (-0.07) − + 1 SD (0.64)	0.05	0.04	58	1.47	0.314

Note. Pairwise comparisons were based on Model 12. *M* = estimated marginal mean; Δ*M* = mean difference (A − B); *SE* = standard error. Pairwise p-values were Tukey-adjusted; Estimated marginal means and contrasts are averaged over target sex, participant sex, and sample.

Table S16. Bayesian multilevel models on contact comfort with the interaction between historical pathogen prevalence and WTR or relationship category controlling for societal nonindependence

Model	Parameter	b_{Median}	95% CrI	Posterior direction	R-hat	ESS
Model 13	(Intercept)	2.45	2.34 – 2.56	100%	1.002	1,752
	WTR	1.12	1.07 – 1.17	100%	1.001	1,715
	Historical pathogen prevalence	-0.07	-0.18 – 0.06	85.80%	1.003	1,619
	Target sex [Female]	0.41	0.37 – 0.46	100%	1.002	2,811
	Participant sex [Female]	-0.16	-0.20 – -0.11	100%	1.002	3,050
	Sample [University]	0.26	0.23 – 0.30	100%	1.000	5,412
	WTR × Historical pathogen prevalence	-0.11	-0.18 – -0.04	99.92%	1.001	1,339
	Target sex [Female] × Participant sex [Female]	-0.04	-0.10 – 0.02	88.55%	1.003	2,509
Model 14	(Intercept)	3.78	3.65 – 3.91	100%	1.003	1,301
	Relationship [Acquaintance]	-0.79	-0.87 – -0.71	100%	1.005	917
	Relationship [Stranger]	-1.56	-1.65 – -1.47	100%	1.004	801
	Relationship [Enemy]	-1.74	-1.83 – -1.65	100%	1.003	973
	Historical pathogen prevalence	-0.23	-0.40 – -0.07	99.78%	1.003	1,078
	Target sex [Female]	0.52	0.48 – 0.56	100%	1.000	4,096
	Participant sex [Female]	-0.21	-0.25 – -0.17	100%	1.000	4,156
	Sample [University]	0.30	0.27 – 0.33	100%	0.999	7,601
	Relationship [Acquaintance] × Historical pathogen prevalence	0.11	-0.01 – 0.22	95.62%	1.002	1,039
	Relationship [Stranger] × Historical pathogen prevalence	0.21	0.07 – 0.35	99.85%	1.002	900
	Relationship [Enemy] × Historical pathogen prevalence	0.19	0.06 – 0.32	99.72%	1.001	1,022
	Target sex [Female] × Participant sex [Female]	-0.13	-0.18 – -0.08	100%	1.000	3,669

Note. Models 13 and 14 controlled for societal nonindependence by adding both a Gaussian process over latitude and longitude [gp(latitude, longitude)] and a society-level random effect whose covariance is structured by linguistic proximity [(1 | gr(society, cov = linguistic proximity))].

Table S17. Estimated marginal means of contact comfort by levels of historical pathogen prevalence and all Tukey-adjusted pairwise comparisons within relationship category

	Relationship	Levels of Historical pathogen prevalence	*Mdn*	95% HPD
Estimated marginal means	Friend	−1 SD (−0.77)	4.02	3.64 – 4.40
		Mean (−0.07)	3.86	3.51 – 4.21
		+1 SD (0.64)	3.70	3.34 – 4.05
	Acquaintance	−1 SD (−0.77)	3.15	2.77 – 3.49
		Mean (−0.07)	3.06	2.71 – 3.39
		+1 SD (0.64)	2.97	2.61 – 3.30
	Stranger	−1 SD (−0.77)	2.30	1.93 – 2.65
		Mean (−0.07)	2.29	1.94 – 2.62
		+1 SD (0.64)	2.27	1.93 – 2.62
	Enemy	−1 SD (−0.77)	2.14	1.76 – 2.48
		Mean (−0.07)	2.11	1.75 – 2.44
		+1 SD (0.64)	2.07	1.71 – 2.40
	**Relationship**	**Contrast (A − B)**	***ΔMdn***	**95% HPD**
Pairwise contrasts	Friend	−1 SD − Mean	0.16	0.05 – 0.28
		−1 SD − +1 SD	0.33	0.09 – 0.56
		Mean − +1 SD	0.16	0.05 – 0.28
	Acquaintance	−1 SD − Mean	0.09	0.003 – 0.18
		−1 SD − +1 SD	0.18	0.006 – 0.36
		Mean − +1 SD	0.09	0.003 – 0.18
	Stranger	−1 SD − Mean	0.02	−0.07 – 0.10
		−1 SD − +1 SD	0.03	−0.13 – 0.20
		Mean − +1 SD	0.02	−0.07 – 0.10
	Enemy	−1 SD − Mean	0.03	−0.06 – 0.12
		−1 SD − +1 SD	0.06	−0.13 – 0.24
		Mean − +1 SD	0.03	−0.06 – 0.12

Note. Pairwise comparisons were based on Model 14. Mdn represents the posterior median of the estimated marginal mean; ΔMdn represents the posterior median of the difference (A − B); HPD represents the highest posterior density interval. A contrast is considered credible when its 95% HPD interval excludes zero.

Table S18. Multilevel models of historical pathogen prevalence and its interaction with WTR or relationship category on contact greeting

Predictors	Model 15			Model 16			Model 17		
	OR	95% CI	*p*	OR	95% CI	*p*	OR	95% CI	*p*
(Intercept)	2.12	1.81 – 2.49	<0.001	0.80	0.69 – 0.92	0.002	2.92	2.44 – 3.49	<0.001
Relationship [Acquaintance]	0.50	0.47 – 0.53	<0.001				0.45	0.42 – 0.49	<0.001
Relationship [Stranger]	0.26	0.24 – 0.28	<0.001				0.22	0.20 – 0.25	<0.001
Relationship [Enemy]	0.22	0.21 – 0.24	<0.001				0.17	0.15 – 0.19	<0.001
WTR	1.84	1.73 – 1.95	<0.001	2.60	2.45 – 2.75	<0.001			
Historical pathogen prevalence	1.18	0.93 – 1.49	0.168	1.14	0.93 – 1.38	0.207	0.98	0.76 – 1.27	0.894
Sample [University]	0.92	0.87 – 0.97	0.003	0.90	0.86 – 0.95	<0.001	0.93	0.89 – 0.98	0.005
Target sex [Female]	0.84	0.78 – 0.90	<0.001	0.81	0.76 – 0.87	<0.001	0.89	0.83 – 0.95	0.001
Participant sex [Female]	0.58	0.55 – 0.63	<0.001	0.61	0.58 – 0.66	<0.001	0.57	0.54 – 0.61	<0.001
Target sex [Female] × Participant sex [Female]	1.52	1.38 – 1.67	<0.001	1.52	1.39 – 1.67	<0.001	1.48	1.35 – 1.61	<0.001
WTR × Historical pathogen prevalence				0.82	0.75 – 0.89	<0.001			
Relationship [Acquaintance] × Historical pathogen prevalence							1.10	0.99 – 1.23	0.086
Relationship [Stranger] × Historical pathogen prevalence							1.05	0.90 – 1.22	0.545
Relationship [Enemy] × Historical pathogen prevalence							1.28	1.08 – 1.50	0.003
**Random Effects**									
σ^2	3.29			3.29			3.29		
τ_{00}	0.32 society			0.27 society			0.45 society		
τ_{11}	0.02 society.WTR			0.02 society.WTR			0.04 society.relationshipAcquaintance<br>0.12 society.relationshipStranger<br>0.13 society.relationshipEnemy		
ρ_{01}	0.31 society			0.12 society			-0.11<br>-0.80<br>-0.68		
ICC	0.09			0.08			0.09		
N	61 society			61 society			61 society		
Observations	35,448			35,448			42,381		
Marginal R^2 / Conditional R^2	0.16 / 0.24			0.09 / 0.16			0.13 / 0.21		

Table S19. Estimated marginal means of contact greeting by levels of historical pathogen prevalence and all Tukey-adjusted pairwise comparisons within relationship category

	Relationship	Levels of historical pathogen prevalence	*M*	*SE*	95% CI	
Estimated marginal means	Friend	-1 SD (-0.77)	0.81	0.14	0.55 – 1.08	
		Mean (-0.07)	0.80	0.09	0.63 – 0.98	
		+1 SD (0.64)	0.79	0.12	0.55 – 1.03	
	Acquaintance	-1 SD (-0.77)	-0.06	0.14	-0.32 – 0.21	
		Mean (-0.07)	0.00	0.09	-0.17 – 0.18	
		+1 SD (0.64)	0.06	0.12	-0.18 – 0.30	
	Stranger	-1 SD (-0.77)	-0.73	0.09	-0.91 – -0.55	
		Mean (-0.07)	-0.71	0.06	-0.83 – -0.59	
		+1 SD (0.64)	-0.69	0.08	-0.85 – -0.53	
	Enemy	-1 SD (-0.77)	-1.16	0.10	-1.36 – -0.95	
		Mean (-0.07)	-1.00	0.07	-1.13 – -0.86	
		+1 SD (0.64)	-0.84	0.09	-1.02 – -0.66	
	**Relationship**	**Contrast (A-B)**	**Δ*M***	***SE***	***z***	***p***
Pairwise contrasts	Friend	-1 SD (-0.77) - Mean (-0.07)	0.01	0.09	0.13	0.990
		-1 SD (-0.77) - +1 SD (0.64)	0.03	0.18	0.13	0.990
		Mean (-0.07) - +1 SD (0.64)	0.01	0.09	0.13	0.990
	Acquaintance	-1 SD (-0.77) - Mean (-0.07)	-0.06	0.09	-0.62	0.810
		-1 SD (-0.77) - +1 SD (0.64)	-0.12	0.19	-0.62	0.810
		Mean (-0.07) - +1 SD (0.64)	-0.06	0.09	-0.62	0.810
	Stranger	-1 SD (-0.77) - Mean (-0.07)	-0.02	0.06	-0.34	0.938
		-1 SD (-0.77) - +1 SD (0.64)	-0.04	0.13	-0.34	0.938
		Mean (-0.07) - +1 SD (0.64)	-0.02	0.06	-0.34	0.938
	Enemy	-1 SD (-0.77) - Mean (-0.07)	-0.16	0.07	-2.26	0.061
		-1 SD (-0.77) - +1 SD (0.64)	-0.32	0.14	-2.26	0.061
		Mean (-0.07) - +1 SD (0.64)	-0.16	0.07	-2.26	0.061

Note. Pairwise comparisons were based on Model 17. Estimates are on a logit scale. *M* = estimated marginal mean; Δ*M* = mean difference (A − B); *SE* = standard error. Pairwise p-values were Tukey-adjusted; Estimated marginal means and contrasts are averaged over target sex, participant sex, and sample.

Table S20. Bayesian multilevel models on contact greeting with the interaction between historical pathogen prevalence and WTR or relationship controlling for societal nonindependence

Model	Parameter	LR_{Median}	95% CrI	Posterior direction	R-hat	ESS
Model 18	(Intercept)	-0.25	-0.46 – -0.05	98.83%	1.002	465
	WTR	0.95	0.89 – 1.02	100%	0.999	3,342
	Historical pathogen prevalence	0.09	-0.18 – 0.35	76.60%	1.004	727
	Target sex [Female]	-0.21	-0.28 – -0.14	100%	1.000	3,374
	Participant sex [Female]	-0.49	-0.55 – -0.42	100%	1.000	3,714
	Sample [University]	-0.10	-0.15 – -0.05	99.95%	1.001	6,479
	WTR × Historical pathogen prevalence	-0.20	-0.29 – -0.11	100%	0.999	3,230
	Target sex [Female] × Participant sex [Female]	0.42	0.33 – 0.51	100%	0.999	3,050
Model 19	(Intercept)	0.95	-0.50 – 2.19	93.38%	1.004	1,295
	Relationship [Acquaintance]	-0.78	-0.88 – -0.70	100%	1.006	1,877
	Relationship [Stranger]	-1.51	-1.62 – -1.40	100%	1.004	1,068
	Relationship [Enemy]	-1.78	-1.90 – -1.66	100%	1.001	1,212
	Historical pathogen prevalence	-0.06	-0.31 – 0.20	68.12%	1.002	802
	Target sex [Female]	-0.12	-0.18 – -0.05	100%	1.001	2,719
	Participant sex [Female]	-0.56	-0.62 – -0.49	100%	1.000	2,638
	Sample [University]	-0.07	-0.12 – -0.02	99.62%	0.999	6,738
	Relationship [Acquaintance] × Historical pathogen prevalence	0.09	-0.04 – 0.21	92.62%	1.001	2,023
	Relationship [Stranger] × Historical pathogen prevalence	0.05	-0.13 – 0.21	71.12%	1.003	1,182
	Relationship [Enemy] × Historical pathogen prevalence	0.25	0.07 – 0.42	99.50%	1.003	1,277
	Target sex [Female] × Participant sex [Female]	0.39	0.31 – 0.48	100%	1.001	2,256
Model 20	(Intercept)	-0.40	-0.63 – -0.15	99.83%	1.007	497
	WTR	0.95	0.90 – 1.01	100%	1.003	2,323
	Historical pathogen prevalence	0.08	-0.12 – 0.30	78.95%	1.005	721
	Target sex [Female]	-0.21	-0.28 – -0.14	100%	0.999	3,418
	Participant sex [Female]	-0.49	-0.55 – -0.42	100%	1.000	3,363
	Sample [University]	-0.10	-0.15 – -0.04	99.95%	1.000	6,553
	WTR × Historical pathogen prevalence	-0.20	-0.29 – -0.12	100%	1.000	2,884
	Target sex [Female] × Participant sex [Female]	0.42	0.33 – 0.51	100%	0.999	2,868
Model 21	(Intercept)	0.94	0.68 – 1.19	100%	1.003	706
	Relationship [Acquaintance]	-0.79	-0.87 – -0.70	100%	1.000	1,618
	Relationship [Stranger]	-1.51	-1.62 – -1.40	100%	1.000	1,145
	Relationship [Enemy]	-1.78	-1.90 – -1.66	100%	1.001	1,140
	Historical pathogen prevalence	-0.06	-0.30 – 0.18	68.42%	1.004	954
	Target sex [Female]	-0.12	-0.18 – -0.05	99.98%	1.000	2,971
	Participant sex [Female]	-0.55	-0.62 – -0.49	100%	1.000	3,155
	Sample [University]	-0.07	-0.12 – -0.02	99.60%	1.000	6,267
	Relationship [Acquaintance] × Historical pathogen prevalence	0.09	-0.03 – 0.22	92.88%	1.002	1,669
	Relationship [Stranger] × Historical pathogen prevalence	0.05	-0.12 – 0.21	73.83%	1.004	1,196
	Relationship [Enemy] × Historical pathogen prevalence	0.25	0.08 – 0.43	99.88%	1.002	1,142
	Target sex [Female] × Participant sex [Female]	0.39	0.30 – 0.48	100%	1.000	2,562

Note. Models 18 and 19 controlled for societal nonindependence through a Gaussian process over latitude and longitude [gp(latitude, longitude)]; Models 20 and 21 controlled for societal nonindependence through a society-level random effect whose covariance is structured by linguistic proximity [(1 | gr(society, cov = linguistic proximity))].

Table S21. Estimated marginal means of contact greeting by levels of historical pathogen prevalence and all Tukey-adjusted pairwise comparisons within relationship category

	Relationship	Levels of historical pathogen prevalence	Model 19		Model 21	
			Mdn	95% HPD	*Mdn*	95% HPD
Estimated marginal means	Friend	-1 SD (-0.77)	1.05	0.73 – 1.35	0.71	0.38 – 1.04
		Mean (-0.07)	1.01	0.81 – 1.21	0.67	0.41 – 0.92
		+1 SD (0.64)	0.97	0.73 – 1.20	0.63	0.35 – 0.89
	Acquaintance	-1 SD (-0.77)	0.20	-0.10 – 0.51	-0.16	-0.48 – 0.19
		Mean (-0.07)	0.22	0.03 – 0.43	-0.13	-0.38 – 0.11
		+1 SD (0.64)	0.24	0.02 – 0.46	-0.10	-0.36 – 0.15
	Stranger	-1 SD (-0.77)	-0.48	-0.77 – -0.21	-0.84	-1.14 – -0.51
		Mean (-0.07)	-0.50	-0.67 – -0.30	-0.85	-1.09 – -0.61
		+1 SD (0.64)	-0.51	-0.69 – -0.32	-0.85	-1.09 – -0.59
	Enemy	-1 SD (-0.77)	-0.91	-1.22 – -0.64	-1.27	-1.61 – -0.97
		Mean (-0.07)	-0.79	-0.97 – -0.58	-1.13	-1.40 – -0.90
		+1 SD (0.64)	-0.66	-0.88 – -0.47	-0.99	-1.25 – -0.75
	**Relationship**	**Contrast (A-B)**	**Model 19**		**Model 21**	
			ΔMdn	**95% HPD**	***ΔMdn***	**95% HPD**
Pairwise contrasts	Friend	-1 SD (-0.77) - Mean (-0.07)	0.05	-0.14 – 0.21	0.04	-0.13 – 0.20
		-1 SD (-0.77) - +1 SD (0.64)	0.09	-0.29 – 0.43	0.08	-0.27 – 0.41
		Mean (-0.07) - +1 SD (0.64)	0.05	-0.14 – 0.21	0.04	-0.13 – 0.20
	Acquaintance	-1 SD (-0.77) - Mean (-0.07)	-0.02	-0.20 – 0.14	-0.03	-0.19 – 0.14
		-1 SD (-0.77) - +1 SD (0.64)	-0.04	-0.40 – 0.29	-0.05	-0.38 – 0.28
		Mean (-0.07) - +1 SD (0.64)	-0.02	-0.20 – 0.14	-0.03	-0.19 – 0.14
	Stranger	-1 SD (-0.77) - Mean (-0.07)	0.01	-0.14 – 0.16	0.00	-0.15 – 0.15
		-1 SD (-0.77) - +1 SD (0.64)	0.03	-0.29 – 0.31	0.01	-0.29 – 0.31
		Mean (-0.07) - +1 SD (0.64)	0.01	-0.14 – 0.16	0.00	-0.15 – 0.15
	Enemy	-1 SD (-0.77) - Mean (-0.07)	-0.13	-0.29 – 0.03	-0.14	-0.29 – 0.02
		-1 SD (-0.77) - +1 SD (0.64)	-0.26	-0.57 – 0.06	-0.27	-0.58 – 0.04
		Mean (-0.07) - +1 SD (0.64)	-0.13	-0.29 – 0.03	-0.14	-0.29 – 0.02

Note. Pairwise comparisons were based on Models 19 and 21. Estimates are on the logit scale. Mdn represents the posterior median of the estimated marginal mean; ΔMdn represents the posterior median of the difference (A − B); HPD represents the highest posterior density interval. A contrast is considered credible when its 95% HPD interval excludes zero.

Table S22. Multilevel models of individualism, tightness, and relational mobility on contact comfort

	Model 22			Model 23			Model 24		
Predictors	***b***	**95% CI**	***p***	***b***	**95% CI**	***p***	***b***	**95% CI**	***p***
(Intercept)	3.44	3.37 – 3.50	<0.001	3.47	3.39 – 3.55	<0.001	3.39	3.30 – 3.47	<0.001
Relationship [Acquaintance]	-0.69	-0.73 – -0.65	<0.001	-0.70	-0.75 – -0.66	<0.001	-0.73	-0.79 – -0.68	<0.001
Relationship [Stranger]	-1.39	-1.43 – -1.35	<0.001	-1.44	-1.49 – -1.40	<0.001	-1.47	-1.52 – -1.42	<0.001
Relationship [Enemy]	-1.41	-1.45 – -1.37	<0.001	-1.46	-1.51 – -1.41	<0.001	-1.48	-1.54 – -1.43	<0.001
WTR	0.75	0.71 – 0.80	<0.001	0.75	0.69 – 0.80	<0.001	0.73	0.68 – 0.79	<0.001
Sample [University]	0.29	0.25 – 0.32	<0.001	0.31	0.27 – 0.34	<0.001	0.35	0.31 – 0.38	<0.001
Target sex [Female]	0.46	0.41 – 0.50	<0.001	0.44	0.39 – 0.49	<0.001	0.48	0.43 – 0.54	<0.001
Participant sex [Female]	-0.17	-0.21 – -0.13	<0.001	-0.18	-0.23 – -0.14	<0.001	-0.14	-0.19 – -0.09	<0.001
Target sex [Female] × Participant sex [Female]	-0.07	-0.12 – -0.01	0.023	-0.02	-0.08 – 0.05	0.623	-0.09	-0.16 – -0.02	0.017
Individualism	0.10	0.04 – 0.16	0.001						
Tightness				-0.02	-0.08 – 0.05	0.638			
Relational mobility							0.13	0.06 – 0.21	<0.001
**Random Effects**									
σ^2	1.72			1.70			1.63		
τ_{00}	0.04 $_{society}$			0.04 $_{society}$			0.04 $_{society}$		
τ_{11}	0.02 $_{society.WTR}$			0.02 $_{society.WTR}$			0.01 $_{society.WTR}$		
ρ_{01}	-0.00 $_{society}$			-0.06 $_{society}$			-0.05 $_{society}$		
ICC	0.03			0.03			0.02		
N	57 $_{society}$			41 $_{society}$			29 $_{society}$		
Observations	34,243			26,399			20,985		
Marginal R^2 / Conditional R^2	0.31 / 0.33			0.31 / 0.33			0.33 / 0.35		

Table S23. Bayesian multilevel models of individualism and relational mobility on contact comfort controlling for societal nonindependence

Model	Parameter	b_{Median}	95% CrI	Posterior direction	R-hat	ESS
Model 25 (Individualism)	(Intercept)	3.43	3.33 – 3.54	100%	1.002	1,750
	Relationship [Acquaintance]	-0.69	-0.72 – -0.64	100%	1.000	4,023
	Relationship [Stranger]	-1.39	-1.43 – -1.35	100%	1.000	4,057
	Relationship [Enemy]	-1.41	-1.45 – -1.36	100%	1.000	3,993
	WTR	0.75	0.71 – 0.80	100%	1.001	2,151
	Individualism	0.06	-0.02 – 0.14	92.70%	1.004	1,298
	Target sex [Female]	0.45	0.41 – 0.50	100%	1.000	3,775
	Participant sex [Female]	-0.17	-0.22 – -0.13	100%	1.000	4,288
	Sample [University]	0.29	0.25 – 0.32	100%	1.000	9,039
	Target sex [Female] × Participant sex [Female]	-0.06	-0.12 – -0.01	98.30%	1.000	3,561
Model 26 (Relational mobility)	(Intercept)	3.39	3.21 – 3.55	100%	1.006	536
	Relationship [Acquaintance]	-0.73	-0.78 – -0.68	100%	0.999	3,796
	Relationship [Stranger]	-1.47	-1.52 – -1.42	100%	1.000	3,846
	Relationship [Enemy]	-1.48	-1.54 – -1.43	100%	1.000	3,330
	WTR	0.73	0.68 – 0.79	100%	1.001	1,719
	Relational mobility	0.11	0.01 – 0.21	98.72%	1.005	1,397
	Target sex [Female]	0.49	0.43 – 0.54	100%	1.000	3,447
	Participant sex [Female]	-0.14	-0.19 – -0.09	100%	1.000	3,377
	Sample [University]	0.35	0.31 – 0.38	100%	0.999	7,471
	Target sex [Female] × Participant sex [Female]	-0.09	-0.16 – -0.01	99.28%	1.000	2,965

Note. Models 25 and 26 controlled for societal nonindependence by adding both a Gaussian process over latitude and longitude [gp(latitude, longitude)] and a society-level random effect whose covariance is structured by linguistic proximity [(1 | gr(society, cov = linguistic proximity))].

Table S24. Multilevel models of individualism, tightness, and relational mobility on contact greeting

	Model 27			Model 28			Model 29		
Predictors	**OR**	**95%CI**	***p***	**OR**	**95%CI**	***p***	**OR**	**95%CI**	***p***
(Intercept)	2.07	1.75 – 2.45	<0.001	2.17	1.79 – 2.64	<0.001	1.90	1.53 – 2.37	<0.001
Relationship [Acquaintance]	0.50	0.47 – 0.53	<0.001	0.51	0.47 – 0.55	<0.001	0.47	0.43 – 0.52	<0.001
Relationship [Stranger]	0.26	0.24 – 0.28	<0.001	0.26	0.24 – 0.28	<0.001	0.26	0.24 – 0.28	<0.001
Relationship [Enemy]	0.22	0.21 – 0.24	<0.001	0.22	0.20 – 0.23	<0.001	0.21	0.19 – 0.23	<0.001
WTR	1.84	1.73 – 1.96	<0.001	1.83	1.72 – 1.94	<0.001	1.78	1.66 – 1.91	<0.001
Sample [University]	0.92	0.87 – 0.97	0.002	0.94	0.88 – 1.00	0.051	1.00	0.93 – 1.07	0.900
Target sex [Female]	0.84	0.78 – 0.90	<0.001	0.84	0.77 – 0.92	<0.001	0.85	0.77 – 0.93	0.001
Participant sex [Female]	0.59	0.55 – 0.63	<0.001	0.60	0.56 – 0.65	<0.001	0.58	0.53 – 0.63	<0.001
Target sex [Female] × Participant sex [Female]	1.51	1.37 – 1.67	<0.001	1.43	1.28 – 1.60	<0.001	1.48	1.30 – 1.67	<0.001
Individualism	0.92	0.77 – 1.11	0.390						
Tightness				0.86	0.73 – 1.03	0.100			
Relational mobility							1.43	1.16 – 1.77	0.001
**Random Effects**									
σ^2	3.29			3.29			3.29		
τ_{00}	0.34 society			0.33 society			0.29 society		
τ_{11}	0.02 society.WTR			0.01 society.WTR			0.01 society.WTR		
ρ_{01}	0.26 society			0.13 society			0.24 society		
ICC	0.10			0.09			0.08		
N	57 society			41 society			29 society		
Observations	34,243			26,399			20,985		
Marginal R^2 / Conditional R^2	0.16 / 0.24			0.16 / 0.24			0.18 / 0.25		

Table S25. Bayesian multilevel models of relational mobility on contact greeting controlling for societal nonindependence

Model	Parameter	LR_{Median}	95% CrI	Posterior direction	R-hat	ESS
Model 30	(Intercept)	0.55	0.20 – 0.91	99.78%	1.002	657
	Relationship [Acquaintance]	-0.75	-0.84 – -0.67	100%	1.001	3,368
	Relationship [Stranger]	-1.35	-1.43 – -1.26	100%	1.000	3,990
	Relationship [Enemy]	-1.56	-1.65 – -1.46	100%	1.001	3,815
	WTR	0.58	0.50 – 0.66	100%	1.001	3,241
	Relational mobility	0.19	-0.02 – 0.39	95.80%	1.003	1,047
	Target sex [Female]	-0.17	-0.27 – -0.07	99.95%	1.000	3,168
	Participant sex [Female]	-0.55	-0.64 – -0.46	100%	0.999	3,089
	Sample [University]	0.002	-0.07 – 0.07	52.35%	1.000	5,481
	Target sex [Female] × Participant sex [Female]	0.39	0.27 – 0.52	100%	0.999	2,956
Model 31	(Intercept)	0.64	0.34 – 0.95	100%	1.003	842
	Relationship [Acquaintance]	-0.75	-0.84 – -0.67	100%	1.001	4,690
	Relationship [Stranger]	-1.35	-1.44 – -1.26	100%	1.001	4,486
	Relationship [Enemy]	-1.55	-1.65 – -1.46	100%	1.001	4,607
	WTR	0.58	0.50 – 0.66	100%	1.003	3,343
	Relational mobility	0.26	0.06 – 0.46	99.05%	1.002	933
	Target sex [Female]	-0.17	-0.27 – -0.07	99.95%	1.000	3,690
	Participant sex [Female]	-0.55	-0.64 – -0.45	100%	1.000	3,924
	Sample [University]	-0.002	-0.07 – 0.06	52.68%	1.000	6,253
	Target sex [Female] × Participant sex [Female]	0.39	0.26 – 0.52	100%	0.999	3,352

Note. Models 30 controlled for societal nonindependence through a Gaussian process over latitude and longitude [gp(latitude, longitude)]; Models 31 controlled for societal nonindependence through a society-level random effect whose covariance is structured by linguistic proximity [(1 | gr(society, cov = linguistic proximity))].

Table S26. Multilevel models with moderations of individualism, tightness, and relational mobility on the association between WTR and contact comfort

Predictors	Model 32			Model 33			Model 34		
	b	**95% CI**	***p***	***b***	**95% CI**	***p***	***b***	**95% CI**	***p***
(Intercept)	2.48	2.41 – 2.54	<0.001	2.47	2.39 – 2.54	<0.001	2.37	2.29 – 2.45	<0.001
WTR	1.14	1.08 – 1.19	<0.001	1.16	1.10 – 1.22	<0.001	1.17	1.11 – 1.23	<0.001
Sample [University]	0.26	0.23 – 0.30	<0.001	0.29	0.25 – 0.33	<0.001	0.32	0.28 – 0.36	<0.001
Target sex [Female]	0.41	0.36 – 0.46	<0.001	0.40	0.34 – 0.45	<0.001	0.43	0.37 – 0.49	<0.001
Participant sex [Female]	-0.16	-0.21 – -0.12	<0.001	-0.16	-0.21 – -0.11	<0.001	-0.13	-0.18 – -0.07	<0.001
Target sex [Female] × Participant sex [Female]	-0.03	-0.09 – 0.03	0.305	0.01	-0.06 – 0.08	0.801	-0.06	-0.13 – 0.02	0.149
Individualism	0.08	0.02 – 0.14	0.008						
WTR × Individualism	0.08	0.03 – 0.13	0.002						
Tightness				-0.01	-0.07 – 0.06	0.877			
WTR × Tightness				-0.07	-0.13 – -0.01	0.019			
Relational mobility							0.12	0.05 – 0.19	0.002
WTR × Relational mobility							0.08	0.02 – 0.14	0.010
**Random Effects**									
σ^2	2.00			2.01			1.94		
τ_{00}	0.04 society			0.04 society			0.04 society		
τ_{11}	0.03 society.WTR			0.03 society.WTR			0.02 society.WTR		
ρ_{01}	-0.07 society			-0.11 society			0.08 society		
ICC	0.03			0.02			0.02		
N	57 society			41 society			29 society		
Observations	34,243			26,399			20,985		
Marginal R^2 / Conditional R^2	0.2 / 0.22			0.19 / 0.21			0.21 / 0.23		

Table S27. Bayesian multilevel models on contact comfort with the interactions between WTR and cultural factors controlling for societal nonindependence

Model	Parameter	b_{Median}	95% CrI	Posterior direction	R-hat	ESS
Model 35 (Individualism)	(Intercept)	2.48	2.38 – 2.58	100%	1.001	2,791
	WTR	1.14	1.08 – 1.19	100%	1.002	2,057
	Individualism	0.05	-0.03 – 0.13	89.28%	1.003	2,062
	Target sex [Female]	0.41	0.36 – 0.46	100%	1.000	4,395
	Participant sex [Female]	-0.16	-0.21 – -0.12	100%	0.999	4,409
	Sample [University]	0.26	0.23 – 0.30	100%	1.000	6,614
	WTR × Individualism	0.08	0.03 – 0.14	99.92%	1.001	2,505
	Target sex [Female] × Participant sex [Female]	-0.03	-0.09 – 0.03	85.40%	1.000	3,618
Model 36 (Tightness)	(Intercept)	2.38	2.21 – 2.53	100%	1.001	2,928
	WTR	1.21	1.13 – 1.29	100%	1.001	1,905
	Tightness	-0.02	-0.12 – 0.07	68.47%	1.001	2,070
	Target sex [Female]	0.43	0.37 – 0.49	100%	1.000	3,977
	Participant sex [Female]	-0.16	-0.22 – -0.10	100%	1.000	3,857
	Sample [University]	0.31	0.26 – 0.35	100%	1.000	7,409
	WTR × Tightness	-0.09	-0.17 – -0.01	98.50%	1.003	1,925
	Target sex [Female] × Participant sex [Female]	0.01	-0.07 – 0.08	55.83%	1.000	3,347
Model 37 (Relational mobility)	(Intercept)	2.39	2.22 – 2.58	100%	1.001	1,822
	WTR	1.17	1.10 – 1.23	100%	1.000	3,006
	Relational mobility	0.11	0.004 – 0.21	97.98%	1.001	2,659
	Target sex [Female]	0.43	0.37 – 0.49	100%	1.001	4,439
	Participant sex [Female]	-0.13	-0.18 – -0.07	100%	1.000	4,803
	Sample [University]	0.32	0.28 – 0.36	100%	1.000	8,689
	WTR × Relational mobility	0.08	0.01 – 0.14	98.90%	1.000	2,877
	Target sex [Female] × Participant sex [Female]	-0.06	-0.13 – 0.02	93.03%	1.001	4,260

Note. All models controlled for societal nonindependence by adding both a Gaussian process over latitude and longitude [gp(latitude, longitude)] and a society-level random effect whose covariance is structured by linguistic proximity [(1 | gr(society, cov = linguistic proximity))].

Table S28. Multilevel models with moderations of individualism, tightness, and relational mobility on the association between relationship category and contact comfort

Predictors	Model 38			Model 39			Model 40		
	b	95% CI	*p*	*b*	95% CI	*p*	*b*	95% CI	*p*
(Intercept)	3.83	3.73 – 3.92	<0.001	3.86	3.75 – 3.98	<0.001	3.76	3.63 – 3.90	<0.001
Relationship [Acquaintance]	-0.82	-0.89 – -0.75	<0.001	-0.84	-0.92 – -0.76	<0.001	-0.87	-0.97 – -0.78	<0.001
Relationship [Stranger]	-1.60	-1.68 – -1.51	<0.001	-1.64	-1.76 – -1.53	<0.001	-1.64	-1.77 – -1.52	<0.001
Relationship [Enemy]	-1.78	-1.86 – -1.69	<0.001	-1.84	-1.93 – -1.74	<0.001	-1.86	-1.96 – -1.77	<0.001
Individualism	0.23	0.14 – 0.33	<0.001						
Sample [University]	0.30	0.27 – 0.33	<0.001	0.32	0.28 – 0.35	<0.001	0.36	0.33 – 0.40	<0.001
Target sex [Female]	0.52	0.48 – 0.56	<0.001	0.50	0.45 – 0.55	<0.001	0.55	0.49 – 0.60	<0.001
Participant sex [Female]	-0.21	-0.25 – -0.17	<0.001	-0.21	-0.26 – -0.17	<0.001	-0.18	-0.23 – -0.13	<0.001
Relationship [Acquaintance] × Individualism	-0.10	-0.17 – -0.03	0.005						
Relationship [Stranger] × Individualism	-0.19	-0.28 – -0.10	<0.001						
Relationship [Enemy] × Individualism	-0.17	-0.25 – -0.08	<0.001						
Target sex [Female] × Participant sex [Female]	-0.13	-0.18 – -0.07	<0.001	-0.09	-0.15 – -0.03	0.004	-0.14	-0.21 – -0.07	<0.001
Tightness				-0.10	-0.20 – 0.01	0.076			
Relationship [Acquaintance] × Tightness				0.10	0.02 – 0.17	0.016			
Relationship [Stranger] × Tightness				0.10	-0.01 – 0.21	0.088			
Relationship [Enemy] × Tightness				0.16	0.07 – 0.25	<0.001			
Relational mobility							0.30	0.17 – 0.43	<0.001
Relationship [Acquaintance] × Relational mobility							-0.17	-0.26 – -0.07	0.001
Relationship [Stranger] × Relational mobility							-0.25	-0.38 – -0.13	<0.001
Relationship [Enemy] × Relational mobility							-0.21	-0.31 – -0.11	<0.001
**Random Effects**									
σ^2	1.90			1.88			1.81		
τ_{00}	0.11 society			0.12 society			0.12 society		
τ_{11}	0.05 society.relationshipAcquaintance			0.05 society.relationshipAcquaintance			0.05 society.relationshipAcquaintance		
	0.09 society.relationshipStranger			0.12 society.relationshipStranger			0.10 society.relationshipStranger		
	0.08 society.relationshipEnemy			0.07 society.relationshipEnemy			0.05 society.relationshipEnemy		
ρ_{01}	-0.54			-0.62			-0.79		
	-0.81			-0.82			-0.85		
	-0.71			-0.73			-0.93		
ICC	0.04			0.04			0.03		
N	57 society			41 society			29 society		
Observations	40,769			31,396			24,386		
Marginal R^2 / Conditional R^2	0.23 / 0.26			0.24 / 0.27			0.26 / 0.28		

Table S29. Estimated marginal means of contact comfort by levels of cultural factors and all Tukey-adjusted pairwise comparisons within relationship category

	Relationship	Levels of cultural factors	Model 38 (Individualism)			Model 39 (Tightness)			Model 40 (Relational mobility)		
			***M* (*SE*)**	***df***	**95% CI**	***M* (*SE*)**	***df***	**95% CI**	***M* (*SE*)**	***df***	**95% CI**
Estimated marginal means	Friend	-1 SD (-1)	3.87 (0.06)	55	3.74 – 3.99	4.24 (0.08)	39	4.08 – 4.40	3.79 (0.09)	27	3.60 – 3.98
		Mean (0)	4.10 (0.05)	55	4.01 – 4.19	4.15 (0.06)	39	4.03 – 4.26	4.09 (0.07)	27	3.96 – 4.23
		+1 SD (1)	4.33 (0.07)	54	4.19 – 4.47	4.05 (0.07)	40	3.90 – 4.20	4.39 (0.10)	27	4.20 – 4.59
	Acquaintance	-1 SD (-1)	3.15 (0.05)	56	3.04 – 3.26	3.30 (0.07)	38	3.17 – 3.43	3.08 (0.06)	27	2.96 – 3.21
		Mean (0)	3.28 (0.04)	55	3.20 – 3.36	3.30 (0.05)	39	3.21 – 3.39	3.22 (0.04)	27	3.13 – 3.31
		+1 SD (1)	3.41 (0.06)	54	3.29 – 3.53	3.29 (0.06)	40	3.17 – 3.41	3.35 (0.06)	27	3.22 – 3.48
	Stranger	-1 SD (-1)	2.46 (0.04)	56	2.38 – 2.54	2.50 (0.05)	38	2.40 – 2.61	2.40 (0.05)	27	2.29 – 2.52
		Mean (0)	2.50 (0.03)	55	2.44 – 2.56	2.50 (0.04)	39	2.43 – 2.58	2.45 (0.04)	27	2.37 – 2.53
		+1 SD (1)	2.55 (0.04)	52	2.46 – 2.64	2.51 (0.05)	41	2.41 – 2.60	2.50 (0.06)	26	2.38 – 2.61
	Enemy	-1 SD (-1)	2.25 (0.05)	56	2.16 – 2.35	2.24 (0.06)	38	2.12 – 2.36	2.14 (0.05)	27	2.04 – 2.24
		Mean (0)	2.32 (0.03)	55	2.25 – 2.39	2.31 (0.04)	39	2.22 – 2.39	2.23 (0.03)	27	2.16 – 2.30
		+1 SD (1)	2.39 (0.05)	53	2.28 – 2.49	2.37 (0.05)	40	2.26 – 2.48	2.32 (0.05)	26	2.22 – 2.42
	**Relationship**	**Contrast (A − B)**	**Model 38 (Individualism)**			**Model 39 (Tightness)**			**Model 40 (Relational mobility)**		
			**Δ*M* (*SE*)**	***t***	***p***	**Δ*M* (*SE*)**	***t***	***p***	**Δ*M* (*SE*)**	***t***	***p***
Pairwise contrasts	Friend	-1 SD (-1) − Mean (0)	-0.23 (0.05)	-4.96	< 0.001	0.09 (0.05)	1.76	0.196	-0.30 (0.07)	-4.48	< 0.001
		-1 SD (-1) − +1 SD (1)	-0.47 (0.09)	-4.96	< 0.001	0.19 (0.11)	1.76	0.196	-0.60 (0.13)	-4.48	< 0.001
		Mean (0) − +1 SD (1)	-0.23 (0.05)	-4.96	< 0.001	0.09 (0.05)	1.76	0.196	-0.30 (0.07)	-4.48	< 0.001
	Acquaintance	-1 SD (-1) − Mean (0)	-0.13 (0.04)	-3.24	0.006	0.004 (0.04)	0.09	0.996	-0.13 (0.04)	-3.01	0.015
		-1 SD (-1) − +1 SD (1)	-0.26 (0.08)	-3.24	0.006	0.01 (0.09)	0.09	0.996	-0.27 (0.09)	-3.01	0.015
		Mean (0) − +1 SD (1)	-0.13 (0.04)	-3.24	0.006	0.004 (0.04)	0.09	0.996	-0.13 (0.04)	-3.01	0.015
	Stranger	-1 SD (-1) − Mean (0)	-0.04 (0.03)	-1.43	0.336	-0.002 (0.03)	-0.06	0.998	-0.05 (0.04)	-1.18	0.475
		-1 SD (-1) − +1 SD (1)	-0.09 (0.06)	-1.43	0.336	-0.004 (0.07)	-0.06	0.998	-0.09 (0.08)	-1.18	0.475
		Mean (0) − +1 SD (1)	-0.04 (0.03)	-1.43	0.336	-0.002 (0.03)	-0.06	0.998	-0.05 (0.04)	-1.18	0.475
	Enemy	-1 SD (-1) − Mean (0)	-0.07 (0.04)	-1.91	0.145	-0.07 (0.04)	-1.68	0.225	-0.09 (0.03)	-2.59	0.040
		-1 SD (-1) − +1 SD (1)	-0.14 (0.07)	-1.91	0.145	-0.13 (0.08)	-1.68	0.225	-0.18 (0.07)	-2.59	0.040
		Mean (0) − +1 SD (1)	-0.07 (0.04)	-1.91	0.145	-0.07 (0.04)	-1.68	0.225	-0.09 (0.03)	-2.59	0.040

Note. Pairwise comparisons were based on Models 38, 39, and 40. *M* = estimated marginal mean; Δ*M* = mean difference (A − B); *SE* = standard error. Pairwise p-values were Tukey-adjusted; Estimated marginal means and contrasts are averaged over target sex, participant sex, and sample.

Table S30. Bayesian multilevel models on contact comfort with the interactions between relationship category and cultural factors controlling for societal nonindependence

Model	Parameter	b_{Median}	95% CrI	Posterior direction	R-hat	ESS
Model 41 (Individualism)	(Intercept)	3.82	3.69 – 3.95	100%	1.001	1,398
	Relationship [Acquaintance]	-0.82	-0.89 – -0.74	100%	1.002	1,134
	Relationship [Stranger]	-1.59	-1.68 – -1.50	100%	1.003	968
	Relationship [Enemy]	-1.78	-1.87 – -1.69	100%	1.002	1,091
	Individualism	0.20	0.09 – 0.30	100%	1.001	1,196
	Sample [University]	0.30	0.27 – 0.33	100%	1.000	7,134
	Target sex [Female]	0.52	0.47 – 0.56	100%	1.000	3,533
	Participant sex [Female]	-0.21	-0.25 – -0.17	100%	1.000	3,745
	Relationship [Acquaintance] × Individualism	-0.10	-0.17 – -0.03	99.55%	1.000	1,389
	Relationship [Stranger] × Individualism	-0.19	-0.28 – -0.10	100%	1.001	1,184
	Relationship [Enemy] × Individualism	-0.17	-0.25 – -0.08	100%	1.001	1,337
	Target sex [Female] × Participant sex [Female]	-0.13	-0.18 – -0.07	100%	1.000	3,198
Model 42 (Tightness)	(Intercept)	3.83	3.65 – 3.99	100%	1.005	1,370
	Relationship [Acquaintance]	-0.84	-0.93 – -0.76	100%	1.004	1,200
	Relationship [Stranger]	-1.64	-1.76 – -1.52	100%	1.005	1,070
	Relationship [Enemy]	-1.84	-1.93 – -1.73	100%	1.004	1,109
	Tightness	-0.08	-0.20 – 0.03	91.75%	1.008	1,097
	Sample [University]	0.32	0.28 – 0.35	100%	1.000	6,927
	Target sex [Female]	0.50	0.45 – 0.55	100%	1.001	3,056
	Participant sex [Female]	-0.22	-0.26 – -0.17	100%	1.001	3,097
	Relationship [Acquaintance] × Tightness	0.09	0.01 – 0.18	98.58%	1.002	1,316
	Relationship [Stranger] × Tightness	0.10	-0.02 – 0.21	95.97%	1.002	1,040
	Relationship [Enemy] × Tightness	0.16	0.07 – 0.26	99.95%	1.002	1,140
	Target sex [Female] × Participant sex [Female]	-0.09	-0.15 – -0.03	99.78%	1.000	2,944
Model 43 (Relational mobility)	(Intercept)	3.76	3.57 – 3.97	100%	1.002	738
	Relationship [Acquaintance]	-0.87	-0.98 – -0.76	100%	1.000	1,420
	Relationship [Stranger]	-1.64	-1.78 – -1.51	100%	1.001	1,229
	Relationship [Enemy]	-1.86	-1.97 – -1.75	100%	1.001	1,288
	Relational mobility	0.29	0.15 – 0.44	100%	1.002	1,209
	Sample [University]	0.36	0.33 – 0.40	100%	1.000	3,632
	Target sex [Female]	0.55	0.50 – 0.61	100%	1.001	3,305
	Participant sex [Female]	-0.18	-0.23 – -0.13	100%	1.000	3,720
	Relationship [Acquaintance] × Relational mobility	-0.17	-0.28 – -0.06	99.62%	1.001	1,270
	Relationship [Stranger] × Relational mobility	-0.25	-0.38 – -0.12	99.95%	1.002	1,164
	Relationship [Enemy] × Relational mobility	-0.21	-0.32 – -0.10	100%	1.001	1,212
	Target sex [Female] × Participant sex [Female]	-0.14	-0.21 – -0.07	100%	1.000	3,473

Note. All models controlled for societal nonindependence by adding both a Gaussian process over latitude and longitude [gp(latitude, longitude)] and a society-level random effect whose covariance is structured by linguistic proximity [(1 | gr(society, cov = linguistic proximity))].

Table S31. Estimated marginal means of contact comfort by levels of cultural factors and all Tukey-adjusted pairwise comparisons within relationship category

	Relationship	Levels of cultural factors	Model 41 (Individualism)		Model 42 (Tightness)		Model 43 (Relational mobility)	
			Mdn	95% HPD	*Mdn*	95% HPD	*Mdn*	95% HPD
Estimated marginal means	Friend	-1 SD (-1)	3.89	3.53 – 4.24	4.20	3.81 – 4.55	3.77	3.43 – 4.05
		Mean (0)	4.09	3.74 – 4.42	4.11	3.78 – 4.47	4.06	3.74 – 4.34
		+1 SD (1)	4.28	3.90 – 4.62	4.02	3.68 – 4.39	4.35	3.99 – 4.66
	Acquaintance	-1 SD (-1)	3.17	2.82 – 3.51	3.26	2.87 – 3.59	3.07	2.76 – 3.32
		Mean (0)	3.27	2.91 – 3.57	3.27	2.94 – 3.63	3.20	2.89 – 3.44
		+1 SD (1)	3.36	3.00 – 3.70	3.28	2.94 – 3.64	3.32	2.99 – 3.61
	Stranger	-1 SD (-1)	2.49	2.13 – 2.81	2.46	2.09 – 2.80	2.38	2.05 – 2.61
		Mean (0)	2.49	2.16 – 2.81	2.47	2.16 – 2.84	2.43	2.10 – 2.65
		+1 SD (1)	2.50	2.15 – 2.84	2.48	2.14 – 2.85	2.46	2.13 – 2.75
	Enemy	-1 SD (-1)	2.28	1.92 – 2.61	2.20	1.84 – 2.56	2.12	1.81 – 2.37
		Mean (0)	2.31	1.96 – 2.63	2.28	1.95 – 2.63	2.21	1.89 – 2.44
		+1 SD (1)	2.34	1.99 – 2.68	2.35	2.00 – 2.70	2.29	1.97 – 2.58
	**Relationship**	**Contrast (A − B)**	**Model 41 (Individualism)**		**Model 42 (Tightness)**		**Model 43 (Relational mobility)**	
			**Δ*Mdn***	**95% HPD**	**Δ*Mdn***	**95% HPD**	**Δ*Mdn***	**95% HPD**
Pairwise contrasts	Friend	-1 SD (-1) − Mean (0)	-0.20	-0.30 – -0.09	0.08	-0.04 – 0.20	-0.29	-0.43 – -0.14
		-1 SD (-1) − +1 SD (1)	-0.39	-0.61 – -0.18	0.17	-0.07 – 0.39	-0.59	-0.86 – -0.29
		Mean (0) − +1 SD (1)	-0.20	-0.30 – -0.09	0.08	-0.04 – 0.20	-0.29	-0.43 – -0.14
	Acquaintance	-1 SD (-1) − Mean (0)	-0.09	-0.19 – 0.001	-0.01	-0.11 – 0.09	-0.13	-0.23 – -0.01
		-1 SD (-1) − +1 SD (1)	-0.19	-0.38 – 0.002	-0.02	-0.21 – 0.18	-0.25	-0.46 – -0.02
		Mean (0) − +1 SD (1)	-0.09	-0.19 – 0.001	-0.01	-0.11 – 0.09	-0.13	-0.23 – -0.01
	Stranger	-1 SD (-1) − Mean (0)	-0.01	-0.09 – 0.08	-0.01	-0.11 – 0.10	-0.04	-0.15 – 0.07
		-1 SD (-1) − +1 SD (1)	-0.01	-0.17 – 0.16	-0.03	-0.21 – 0.19	-0.08	-0.30 – 0.15
		Mean (0) − +1 SD (1)	-0.01	-0.09 – 0.08	-0.01	-0.11 – 0.10	-0.04	-0.15 – 0.07
	Enemy	-1 SD (-1) − Mean (0)	-0.03	-0.12 – 0.06	-0.08	-0.18 – 0.02	-0.09	-0.18 – 0.01
		-1 SD (-1) − +1 SD (1)	-0.06	-0.24 – 0.12	-0.16	-0.36 – 0.04	-0.17	-0.36 – 0.03
		Mean (0) − +1 SD (1)	-0.03	-0.12 – 0.06	-0.08	-0.18 – 0.02	-0.09	-0.18 – 0.01

Note. Pairwise comparisons were based on Models 41, 42, and 43. Mdn represents the posterior median of the estimated marginal mean; ΔMdn represents the posterior median of the difference (A − B); HPD represents the highest posterior density interval. A contrast is considered credible when its 95% HPD interval excludes zero.

Table S32. Multilevel models with moderations of individualism, tightness, and relational mobility on the association between WTR and contact greeting

Predictors	Model 44			Model 45			Model 46		
	OR	95% CI	*p*	OR	95% CI	*p*	OR	95% CI	*p*
(Intercept)	0.78	0.67 – 0.91	0.001	0.81	0.68 – 0.96	0.016	0.70	0.58 – 0.85	<0.001
WTR	2.65	2.50 – 2.81	<0.001	2.67	2.50 – 2.86	<0.001	2.66	2.47 – 2.87	<0.001
Sample [University]	0.90	0.85 – 0.95	<0.001	0.92	0.87 – 0.98	0.010	0.97	0.90 – 1.03	0.327
Target sex [Female]	0.81	0.75 – 0.87	<0.001	0.82	0.75 – 0.89	<0.001	0.81	0.74 – 0.89	<0.001
Participant sex [Female]	0.61	0.57 – 0.66	<0.001	0.64	0.59 – 0.69	<0.001	0.61	0.56 – 0.66	<0.001
Target sex [Female] × Participant sex [Female]	1.52	1.38 – 1.67	<0.001	1.43	1.29 – 1.59	<0.001	1.47	1.30 – 1.66	<0.001
Individualism	0.94	0.81 – 1.08	0.379						
WTR × Individualism	1.17	1.10 – 1.24	<0.001						
Tightness				0.88	0.75 – 1.03	0.112			
WTR × Tightness				0.94	0.88 – 1.00	0.069			
Relational mobility							1.40	1.16 – 1.68	<0.001
WTR × Relational mobility							1.09	1.01 – 1.18	0.023
**Random Effects**									
σ^2	3.29			3.29			3.29		
τ_{00}	0.28 society			0.28 society			0.24 society		
τ_{11}	0.02 society.WTR			0.02 society.WTR			0.02 society.WTR		
ρ_{01}	0.14 society			-0.09 society			0.07 society		
ICC	0.08			0.08			0.07		
N	57 society			41 society			29 society		
Observations	34,243			26,399			20,985		
Marginal R^2 / Conditional R^2	0.09 / 0.16			0.09 / 0.16			0.11 / 0.18		

Table S33. Bayesian multilevel models on contact greeting with the interactions between WTR and cultural factors controlling for societal nonindependence

Model	Parameter	LR_{Median}	95% CrI	Posterior direction	R-hat	ESS
Model 47 (Individualism)	(Intercept)	-0.26	-0.47 – -0.07	99.72%	1.012	420
	WTR	0.97	0.91 – 1.04	100%	1.000	2,250
	Individualism	-0.05	-0.22 – 0.12	72.50%	1.012	489
	Target sex [Female]	-0.21	-0.28 – -0.14	100%	1.000	2,923
	Participant sex [Female]	-0.49	-0.55 – -0.42	100%	1.000	3,051
	Sample [University]	-0.10	-0.16 – -0.05	100%	1.000	5,453
	WTR × Individualism	0.16	0.09 – 0.22	100%	1.001	2,613
	Target sex [Female] × Participant sex [Female]	0.42	0.32 – 0.51	100%	1.000	2,636
Model 48 (Individualism)	(Intercept)	-0.34	-0.60 – -0.09	99.50%	1.022	332
	WTR	0.97	0.91 – 1.04	100%	1.000	2,659
	Individualism	0.06	-0.07 – 0.22	81.65%	1.001	818
	Target sex [Female]	-0.21	-0.29 – -0.14	100%	1.000	2,828
	Participant sex [Female]	-0.49	-0.56 – -0.42	100%	1.000	2,968
	Sample [University]	-0.10	-0.15 – -0.05	100%	1.000	5,466
	WTR × Individualism	0.15	0.09 – 0.22	100%	1.000	2,623
	Target sex [Female] × Participant sex [Female]	0.42	0.32 – 0.51	100%	1.001	2,500
Model 49 （Relational mobility）	(Intercept)	-0.36	-0.63 – -0.08	99.25%	1.001	658
	WTR	0.98	0.90 – 1.06	100%	1.001	2,607
	Relational mobility	0.23	0.06 – 0.41	99.48%	1.004	765
	Target sex [Female]	-0.21	-0.30 – -0.11	100%	1.000	3,256
	Participant sex [Female]	-0.50	-0.59 – -0.41	100%	1.000	3,441
	Sample [University]	-0.03	-0.10 – 0.04	82.93%	0.999	6,850
	WTR × Relational mobility	0.09	0.01 – 0.17	98.10%	1.000	2,660
	Target sex [Female] × Participant sex [Female]	0.39	0.26 – 0.50	100%	1.001	2,865
Model 50 （Relational mobility）	(Intercept)	-0.43	-0.76 – -0.10	99.45%	1.001	820
	WTR	0.98	0.90 – 1.07	100%	1.000	2,249
	Relational mobility	0.18	-0.01 – 0.37	96.67%	1.000	1,099
	Target sex [Female]	-0.21	-0.30 – -0.11	100%	1.000	2,890
	Participant sex [Female]	-0.50	-0.58 – -0.41	100%	1.000	3,250
	Sample [University]	-0.03	-0.10 – 0.04	79.50%	1.000	5,729
	WTR × Relational mobility	0.09	0.01 – 0.17	98.15%	1.000	2,405
	Target sex [Female] × Participant sex [Female]	0.39	0.27 – 0.51	100%	1.000	2,365

Note. Models 47 and 48 controlled for societal nonindependence through a Gaussian process over latitude and longitude [gp(latitude, longitude)]; Models 49 and 50 controlled for societal nonindependence through a society-level random effect whose covariance is structured by linguistic proximity [(1 | gr(society, cov = linguistic proximity))].

Table S34. Multilevel models with moderations of individualism, tightness, and relational mobility on the association between relationship category and contact greeting

Predictors	Model 51			Model 52			Model 53		
	**OR**	**95% CI**	***p***	**OR**	**95% CI**	***p***	**OR**	**95% CI**	***p***
(Intercept)	2.89	2.39 – 3.49	<0.001	3.00	2.42 – 3.71	<0.001	2.60	2.05 – 3.30	<0.001
Relationship [Acquaintance]	0.45	0.41 – 0.48	<0.001	0.46	0.42 – 0.50	<0.001	0.43	0.38 – 0.47	<0.001
Relationship [Stranger]	0.22	0.20 – 0.25	<0.001	0.22	0.20 – 0.25	<0.001	0.23	0.20 – 0.26	<0.001
Relationship [Enemy]	0.16	0.15 – 0.18	<0.001	0.16	0.14 – 0.18	<0.001	0.16	0.14 – 0.19	<0.001
Individualism	1.07	0.88 – 1.28	0.508						
Sample [University]	0.93	0.88 – 0.98	0.003	0.95	0.90 – 1.01	0.087	0.98	0.92 – 1.05	0.585
Target sex [Female]	0.89	0.83 – 0.95	<0.001	0.90	0.83 – 0.97	0.005	0.89	0.82 – 0.98	0.013
Participant sex [Female]	0.58	0.54 – 0.62	<0.001	0.60	0.56 – 0.65	<0.001	0.58	0.53 – 0.63	<0.001
Relationship [Acquaintance] × Individualism	0.93	0.86 – 1.00	0.054						
Relationship [Stranger] × Individualism	0.91	0.81 – 1.02	0.094						
Relationship [Enemy] × Individualism	0.82	0.73 – 0.93	0.001						
Target sex [Female] × Participant sex [Female]	1.47	1.35 – 1.61	<0.001	1.39	1.26 – 1.54	<0.001	1.43	1.27 – 1.60	<0.001
Tightness				0.80	0.66 – 0.98	0.031			
Relationship [Acquaintance] × Tightness				1.06	0.97 – 1.16	0.207			
Relationship [Stranger] × Tightness				1.07	0.95 – 1.21	0.238			
Relationship [Enemy] × Tightness				1.23	1.09 – 1.38	0.001			
Relational mobility							1.57	1.25 – 1.98	<0.001
Relationship [Acquaintance] × Relational mobility							1.02	0.91 – 1.14	0.731
Relationship [Stranger] × Relational mobility							0.82	0.72 – 0.93	0.002
Relationship [Enemy] × Relational mobility							0.81	0.70 – 0.95	0.010
**Random Effects**									
σ^2	3.29			3.29			3.29		
τ_{00}	0.46 society			0.42 society			0.37 society		
τ_{11}	0.03 society.relationshipAcquaintance			0.03 society.relationshipAcquaintance			0.04 society.relationshipAcquaintance		
	0.12 society.relationshipStranger			0.10 society.relationshipStranger			0.07 society.relationshipStranger		
	0.14 society.relationshipEnemy			0.10 society.relationshipEnemy			0.12 society.relationshipEnemy		
ρ_{01}	-0.03			0.19			-0.07		
	-0.80			-0.82			-0.64		
	-0.67			-0.60			-0.70		
ICC	0.10			0.09			0.08		
N	57 society			41 society			29 society		
Observations	40,769			31,396			24,386		
Marginal R^2 / Conditional R^2	0.13 / 0.21			0.14 / 0.22			0.15 / 0.22		

Table S35. Estimated marginal means of contact greeting by levels of cultural factors and all Tukey-adjusted pairwise comparisons within relationship category

	Relationship	Levels of cultural factors	Model 51 (Individualism)		Model 52 (Tightness)		Model 53 (Relational mobility)	
			M (*SE*)	95% CI	*M* (*SE*)	95% CI	*M* (*SE*)	95% CI
Estimated marginal means	Friend	-1 SD (-1)	0.72 (0.13)	0.48 – 0.97	1.07 (0.15)	0.77 – 1.37	0.25 (0.16)	-0.07 – 0.57
		Mean (0)	0.79 (0.09)	0.60 – 0.97	0.85 (0.11)	0.64 – 1.05	0.70 (0.12)	0.47 – 0.93
		+1 SD (1)	0.85 (0.14)	0.58 – 1.13	0.63 (0.14)	0.36 – 0.90	1.16 (0.17)	0.83 – 1.49
	Acquaintance	-1 SD (-1)	-0.003 (0.13)	-0.26 – 0.25	0.23 (0.17)	-0.10 – 0.55	-0.62 (0.17)	-0.95 – -0.29
		Mean (0)	-0.02 (0.10)	-0.20 – 0.17	0.07 (0.11)	-0.16 – 0.29	-0.15 (0.12)	-0.38 – 0.09
		+1 SD (1)	-0.03 (0.14)	-0.31 – 0.25	-0.10 (0.15)	-0.39 – 0.20	0.32 (0.17)	-0.02 – 0.66
	Stranger	-1 SD (-1)	-0.69 (0.09)	-0.86 – -0.52	-0.52 (0.10)	-0.72 – -0.31	-1.00 (0.14)	-1.27 – -0.74
		Mean (0)	-0.72 (0.06)	-0.85 – -0.59	-0.66 (0.07)	-0.80 – -0.52	-0.76 (0.10)	-0.94 – -0.57
		+1 SD (1)	-0.75 (0.10)	-0.94 – -0.56	-0.81 (0.10)	-1.00 – -0.62	-0.51 (0.14)	-0.77 – -0.24
	Enemy	-1 SD (-1)	-0.89 (0.10)	-1.08 – -0.70	-0.98 (0.13)	-1.23 – -0.73	-1.36 (0.13)	-1.61 – -1.12
		Mean (0)	-1.02 (0.07)	-1.16 – -0.88	-1.00 (0.09)	-1.17 – -0.83	-1.12 (0.09)	-1.29 – -0.94
		+1 SD (1)	-1.15 (0.11)	-1.36 – -0.93	-1.01 (0.12)	-1.24 – -0.79	-0.87 (0.13)	-1.12 – -0.62

	Relationship	Contrast (A − B)	Model 51 (Individualism)			Model 52 (Tightness)			Model 53 (Relational mobility)		
			Δ*M* (*SE*)	*z*	*p*	Δ*M* (*SE*)	*z*	*p*	Δ*M* (*SE*)	*z*	*p*
Pairwise contrasts	Friend	-1 SD (-1) − Mean (0)	-0.06 (0.10)	-0.66	0.785	0.22 (0.10)	2.16	0.079	-0.45 (0.12)	-3.83	< 0.001
		-1 SD (-1) − +1 SD (1)	-0.13 (0.19)	-0.66	0.785	0.44 (0.20)	2.16	0.079	-0.90 (0.24)	-3.83	< 0.001
		Mean (0) − +1 SD (1)	-0.06 (0.10)	-0.66	0.785	0.22 (0.10)	2.16	0.079	-0.45 (0.12)	-3.83	< 0.001
	Acquaintance	-1 SD (-1) − Mean (0)	0.01 (0.10)	0.14	0.989	0.16 (0.11)	1.48	0.300	-0.47 (0.12)	-3.89	< 0.001
		-1 SD (-1) − +1 SD (1)	0.03 (0.20)	0.14	0.989	0.32 (0.22)	1.48	0.300	-0.94 (0.24)	-3.89	< 0.001
		Mean (0) − +1 SD (1)	0.01 (0.10)	0.14	0.989	0.16 (0.11)	1.48	0.300	-0.47 (0.12)	-3.89	< 0.001
	Stranger	-1 SD (-1) − Mean (0)	0.03 (0.07)	0.48	0.880	0.15 (0.07)	2.13	0.085	-0.25 (0.10)	-2.58	0.027
		-1 SD (-1) − +1 SD (1)	0.06 (0.13)	0.48	0.880	0.29 (0.14)	2.13	0.085	-0.50 (0.19)	-2.58	0.027
		Mean (0) − +1 SD (1)	0.03 (0.07)	0.48	0.880	0.15 (0.07)	2.13	0.085	-0.25 (0.10)	-2.58	0.027
	Enemy	-1 SD (-1) − Mean (0)	0.13 (0.07)	1.74	0.190	0.01 (0.08)	0.17	0.984	-0.25 (0.09)	-2.71	0.018
		-1 SD (-1) − +1 SD (1)	0.26 (0.15)	1.74	0.190	0.03 (0.17)	0.17	0.984	-0.49 (0.18)	-2.71	0.018
		Mean (0) − +1 SD (1)	0.13 (0.07)	1.74	0.190	0.01 (0.08)	0.17	0.984	-0.25 (0.09)	-2.71	0.018

Note. Pairwise comparisons were based on Models 51, 52, and 53. Estimates are on the log odds ratio scale.

Table S36. Bayesian multilevel models on contact greeting with the interactions between relationship category and cultural factors controlling for societal nonindependence

Model	Parameter	LR_{Median}	95% CI	Posterior direction	R-hat	ESS
Model 54 (Individualism)	(Intercept)	0.91	-1.05 – 2.32	87.85%	1.002	1,603
	Relationship [Acquaintance]	-0.80	-0.89 – -0.71	100%	1.000	1,884
	Relationship [Stranger]	-1.51	-1.63 – -1.40	100%	1.001	1,147
	Relationship [Enemy]	-1.81	-1.93 – -1.68	100%	1.003	1,114
	Individualism	0.14	-0.03 – 0.32	95.62%	1.014	707
	Sample [University]	-0.07	-0.12 – -0.02	99.85%	1.000	6,030
	Target sex [Female]	-0.12	-0.19 – -0.05	99.95%	1.000	2,764
	Participant sex [Female]	-0.54	-0.61 – -0.48	100%	1.000	3,003
	Relationship [Acquaintance] × Individualism	-0.08	-0.17 – 0.01	95.62%	1.000	1,703
	Relationship [Stranger] × Individualism	-0.09	-0.21 – 0.02	94.35%	1.006	979
	Relationship [Enemy] × Individualism	-0.19	-0.32 – -0.07	99.88%	1.005	916
	Target sex [Female] × Participant sex [Female]	0.39	0.30 – 0.47	100%	1.000	2,520
Model 55 (Tightness)	(Intercept)	0.87	-1.12 – 2.42	85.80%	1.005	1,449
	Relationship [Acquaintance]	-0.77	-0.88 – -0.66	100%	1.001	2,006
	Relationship [Stranger]	-1.51	-1.65 – -1.39	100%	1.002	1,398
	Relationship [Enemy]	-1.84	-1.98 – -1.71	100%	1.001	1,423
	Tightness–looseness	-0.08	-0.26 – 0.09	82.33%	1.000	1,155
	Sample [University]	-0.05	-0.10 – 0.01	95.28%	1.000	5,382
	Target sex [Female]	-0.11	-0.19 – -0.03	99.80%	1.000	2,623
	Participant sex [Female]	-0.51	-0.58 – -0.43	100%	1.000	2,836
	Relationship [Acquaintance] × Tightness	0.05	-0.05 – 0.15	84.10%	1.001	2,198
	Relationship [Stranger] × Tightness	0.07	-0.06 – 0.19	85.97%	1.000	1,191
	Relationship [Enemy] × Tightness	0.20	0.07 – 0.32	99.90%	1.002	1,082
	Target sex [Female] × Participant sex [Female]	0.33	0.23 – 0.43	100%	1.000	2,341
Model 56 (Relational mobility)	(Intercept)	0.89	0.04 – 1.63	97.90%	1.002	1,462
	Relationship [Acquaintance]	-0.84	-0.97 – -0.71	100%	1.003	2,224
	Relationship [Stranger]	-1.46	-1.60 – -1.32	100%	1.003	1,895
	Relationship [Enemy]	-1.83	-2.00 – -1.67	100%	1.005	1,707
	Relational mobility	0.27	0.03 – 0.51	98.50%	1.001	1,128
	Sample [University]	-0.02	-0.08 – 0.05	70.30%	1.000	4,004
	Target sex [Female]	-0.11	-0.21 – -0.03	99.42%	1.000	2,898
	Participant sex [Female]	-0.55	-0.63 – -0.47	100%	1.003	1,835
	Relationship [Acquaintance] × Relational mobility	0.01	-0.12 – 0.14	58.63%	1.000	2,275
	Relationship [Stranger] × Relational mobility	-0.20	-0.35 – -0.06	99.60%	1.001	2,042
	Relationship [Enemy] × Relational mobility	-0.20	-0.37 – -0.04	99.15%	1.002	1,859
	Target sex [Female] × Participant sex [Female]	0.36	0.25 – 0.47	100%	1.001	2,028

Note. All models controlled for societal nonindependence by adding both a Gaussian process over latitude and longitude [gp(latitude, longitude)] and a society-level random effect whose covariance is structured by linguistic proximity [(1 | gr(society, cov = linguistic proximity))].

Table S37. Estimated marginal means of contact greeting by levels of cultural factors and all Tukey-adjusted pairwise comparisons within relationship category

	Relationship	Levels of cultural factors	Model 54 (Individualism)		Model 55 (Tightness)		Model 56 (Relational mobility)	
			Mdn	95% HPD	*Mdn*	95% HPD	*Mdn*	95% HPD
Estimated marginal means	Friend	-1 SD (-1)	0.79	0.47 – 1.09	1.03	0.60 – 1.47	0.54	0.001 – 1.11
		Mean (0)	0.93	0.63 – 1.21	0.94	0.58 – 1.29	0.81	0.28 – 1.32
		+1 SD (1)	1.08	0.70 – 1.42	0.85	0.49 – 1.20	1.08	0.49 – 1.67
	Acquaintance	-1 SD (-1)	0.07	-0.24 – 0.37	0.20	-0.23 – 0.64	-0.32	-0.84 – 0.23
		Mean (0)	0.13	-0.17 – 0.42	0.16	-0.20 – 0.50	-0.03	-0.54 – 0.48
		+1 SD (1)	0.20	-0.15 – 0.55	0.13	-0.25 – 0.46	0.25	-0.31 – 0.85
	Stranger	-1 SD (-1)	-0.63	-0.91 – -0.33	-0.56	-0.98 – -0.15	-0.72	-1.23 – -0.17
		Mean (0)	-0.58	-0.84 – -0.27	-0.58	-0.94 – -0.24	-0.65	-1.14 – -0.13
		+1 SD (1)	-0.53	-0.86 – -0.18	-0.59	-0.97 – -0.28	-0.59	-1.17 – -0.01
	Enemy	-1 SD (-1)	-0.83	-1.11 – -0.52	-1.01	-1.43 – -0.60	-1.09	-1.62 – -0.54
		Mean (0)	-0.87	-1.17 – -0.59	-0.91	-1.28 – -0.58	-1.02	-1.52 – -0.50
		+1 SD (1)	-0.92	-1.28 – -0.58	-0.79	-1.14 – -0.47	-0.96	-1.56 – -0.40
	**Relationship**	**Contrast (A − B)**	**Model 54 (Individualism)**		**Model 55 (Tightness)**		**Model 56 (Relational mobility)**	
			**Δ*Mdn***	**95% HPD**	**Δ*Mdn***	**95% HPD**	**Δ*Mdn***	**95% HPD**
Pairwise contrasts	Friend	-1 SD (-1) − Mean (0)	-0.14	-0.32 – 0.03	0.08	-0.09 – 0.26	-0.27	-0.52 – -0.03
		-1 SD (-1) − +1 SD (1)	-0.28	-0.63 – 0.06	0.17	-0.18 – 0.52	-0.53	-1.04 – -0.07
		Mean (0) − +1 SD (1)	-0.14	-0.32 – 0.03	0.08	-0.09 – 0.26	-0.27	-0.52 – -0.03
	Acquaintance	-1 SD (-1) − Mean (0)	-0.07	-0.23 – 0.10	0.04	-0.14 – 0.21	-0.28	-0.52 – -0.04
		-1 SD (-1) − +1 SD (1)	-0.13	-0.46 – 0.19	0.07	-0.28 – 0.42	-0.56	-1.04 – -0.09
		Mean (0) − +1 SD (1)	-0.07	-0.23 – 0.10	0.04	-0.14 – 0.21	-0.28	-0.52 – -0.04
	Stranger	-1 SD (-1) − Mean (0)	-0.05	-0.19 – 0.10	0.02	-0.14 – 0.16	-0.07	-0.29 – 0.16
		-1 SD (-1) − +1 SD (1)	-0.10	-0.38 – 0.21	0.04	-0.27 – 0.33	-0.13	-0.57 – 0.32
		Mean (0) − +1 SD (1)	-0.05	-0.19 – 0.10	0.02	-0.14 – 0.16	-0.07	-0.29 – 0.16
	Enemy	-1 SD (-1) − Mean (0)	0.05	-0.10 – 0.20	-0.11	-0.27 – 0.04	-0.06	-0.31 – 0.16
		-1 SD (-1) − +1 SD (1)	0.11	-0.20 – 0.41	-0.22	-0.55 – 0.09	-0.12	-0.62 – 0.32
		Mean (0) − +1 SD (1)	0.05	-0.10 – 0.20	-0.11	-0.27 – 0.04	-0.06	-0.31 – 0.16

Note. Pairwise comparisons were based on Models 54, 55, and 56. Estimates are on the log odds ratio scale. Mdn represents the posterior median of the estimated marginal mean; ΔMdn represents the posterior median of the difference (A − B); HPD represents the highest posterior density interval. A contrast is considered credible when its 95% HPD interval excludes zero.

Table S38. Multilevel models with moderations of societal-level factors on the association between WTR and contact comfort when accounting for relationship category

	Model 57			Model 58			Model 59			Model 60		
Predictors	***b***	**95% CI**	***p***	***b***	**95% CI**	***p***	***b***	**95% CI**	***p***	***b***	**95% CI**	***p***
(Intercept)	3.39	3.32 – 3.46	<0.001	3.43	3.37 – 3.50	<0.001	3.47	3.39 – 3.55	<0.001	3.39	3.30 – 3.47	<0.001
Relationship [Acquaintance]	-0.67	-0.71 – -0.63	<0.001	-0.69	-0.73 – -0.64	<0.001	-0.70	-0.75 – -0.66	<0.001	-0.73	-0.78 – -0.68	<0.001
Relationship [Stranger]	-1.37	-1.41 – -1.33	<0.001	-1.39	-1.43 – -1.35	<0.001	-1.44	-1.49 – -1.40	<0.001	-1.47	-1.52 – -1.42	<0.001
Relationship [Enemy]	-1.39	-1.43 – -1.35	<0.001	-1.41	-1.45 – -1.37	<0.001	-1.46	-1.51 – -1.41	<0.001	-1.48	-1.53 – -1.43	<0.001
WTR	0.76	0.71 – 0.80	<0.001	0.76	0.71 – 0.80	<0.001	0.75	0.70 – 0.80	<0.001	0.74	0.68 – 0.79	<0.001
Sample [University]	0.28	0.25 – 0.31	<0.001	0.29	0.25 – 0.32	<0.001	0.31	0.27 – 0.34	<0.001	0.35	0.31 – 0.38	<0.001
Target sex [Female]	0.46	0.41 – 0.50	<0.001	0.46	0.41 – 0.50	<0.001	0.44	0.39 – 0.49	<0.001	0.48	0.43 – 0.54	<0.001
Participant sex [Female]	-0.17	-0.21 – -0.13	<0.001	-0.17	-0.21 – -0.13	<0.001	-0.18	-0.23 – -0.14	<0.001	-0.14	-0.19 – -0.09	<0.001
Target sex [Female] × Participant sex [Female]	-0.07	-0.12 – -0.01	0.015	-0.07	-0.12 – -0.01	0.024	-0.02	-0.08 – 0.05	0.635	-0.09	-0.16 – -0.01	0.018
Historical pathogen prevalence	-0.12	-0.21 – -0.03	0.010									
WTR × Historical pathogen prevalence	-0.06	-0.11 – 0.00	0.070									
Individualism				0.10	0.04 – 0.16	0.001						
WTR × Individualism				0.03	-0.01 – 0.08	0.134						
Tightness							-0.01	-0.07 – 0.05	0.761			
WTR × Tightness							-0.04	-0.08 – 0.01	0.152			
Relational mobility										0.13	0.06 – 0.20	0.001
WTR × Relational mobility										0.06	0.01 – 0.11	0.016
**Random Effects**												
σ^2	1.72			1.72			1.70			1.63		
τ_{00}	$0.05_{society}$			$0.04_{society}$			$0.04_{society}$			$0.03_{society}$		
τ_{11}	$0.02_{society.WTR}$			$0.02_{society.WTR}$			$0.02_{society.WTR}$			$0.01_{society.WTR}$		
ρ_{01}	$0.02_{society}$			$0.004_{society}$			$-0.06_{society}$			$-0.02_{society}$		
ICC	0.03			0.03			0.03			0.02		
N	$61_{society}$			$57_{society}$			$41_{society}$			$29_{society}$		
Observations	35,448			34,243			26,399			20,985		
Marginal R^2 / Conditional R^2	0.30 / 0.33			0.31 / 0.33			0.31 / 0.33			0.33 / 0.35		

Table S39. Bayesian multilevel models on contact comfort with the interaction between WTR and relational mobility when accounting for relationship category and societal nonindependence

Model	Parameter	b_{Median}	95% CI	Posterior direction	R-hat	ESS
Model 61	(Intercept)	3.39	3.17 – 3.59	100%	1.00	712
	Relationship [Acquaintance]	-0.73	-0.79 – -0.68	100%	1.00	3,525
	Relationship [Stranger]	-1.47	-1.52 – -1.42	100%	1.00	3,573
	Relationship [Enemy]	-1.48	-1.53 – -1.43	100%	1.00	3,000
	WTR	0.73	0.68 – 0.79	100%	1.00	2,210
	Relational mobility	0.11	0.00 – 0.21	97.67%	1.00	1,469
	Target sex [Female]	0.48	0.43 – 0.54	100%	1.00	3,125
	Participant sex [Female]	-0.14	-0.19 – -0.09	100%	1.00	3,491
	Sample [University]	0.35	0.31 – 0.38	100%	1.00	7,524
	WTR × Relational mobility	0.06	0.01 – 0.11	98.62%	1.00	2,518
	Target sex [Female] × Participant sex [Female]	-0.09	-0.16 – -0.02	99.20%	1.00	3,102

Note. Model 61 controlled for societal nonindependence by adding both a Gaussian process over latitude and longitude [gp(latitude, longitude)] and a society-level random effect whose covariance is structured by linguistic proximity [(1 | gr(society, cov = linguistic proximity))].

Table S40. Multilevel models with moderations of societal-level factors on the association between WTR and contact greeting when accounting for relationship category

	Model 62			Model 63			Model 64			Model 65		
Predictors	**OR**	**95% CI**	***p***	**OR**	**95% CI**	***p***	**OR**	**95% CI**	***p***	**OR**	**95% CI**	***p***
(Intercept)	2.12	1.81 – 2.48	<0.001	2.07	1.75 – 2.45	<0.001	2.17	1.79 – 2.64	<0.001	1.91	1.53 – 2.37	<0.001
Relationship [Acquaintance]	0.50	0.47 – 0.53	<0.001	0.50	0.47 – 0.53	<0.001	0.51	0.47 – 0.55	<0.001	0.47	0.43 – 0.52	<0.001
Relationship [Stranger]	0.26	0.24 – 0.28	<0.001	0.26	0.24 – 0.28	<0.001	0.26	0.24 – 0.28	<0.001	0.26	0.24 – 0.28	<0.001
Relationship [Enemy]	0.22	0.21 – 0.24	<0.001	0.22	0.21 – 0.24	<0.001	0.22	0.20 – 0.24	<0.001	0.21	0.19 – 0.23	<0.001
WTR	1.83	1.74 – 1.93	<0.001	1.85	1.75 – 1.96	<0.001	1.83	1.72 – 1.94	<0.001	1.78	1.67 – 1.91	<0.001
Sample [University]	0.92	0.87 – 0.97	0.002	0.92	0.87 – 0.97	0.002	0.94	0.88 – 1.00	0.050	0.99	0.93 – 1.07	0.879
Target sex [Female]	0.84	0.78 – 0.90	<0.001	0.84	0.78 – 0.90	<0.001	0.84	0.77 – 0.92	<0.001	0.85	0.77 – 0.94	0.001
Participant sex [Female]	0.58	0.55 – 0.63	<0.001	0.59	0.55 – 0.63	<0.001	0.60	0.56 – 0.65	<0.001	0.58	0.53 – 0.63	<0.001
Target sex [Female] × Participant sex [Female]	1.52	1.38 – 1.67	<0.001	1.51	1.37 – 1.66	<0.001	1.43	1.28 – 1.60	<0.001	1.48	1.30 – 1.67	<0.001
Historical pathogen prevalence	1.12	0.91 – 1.39	0.298									
WTR × Historical pathogen prevalence	0.87	0.81 – 0.94	<0.001									
Individualism				0.95	0.82 – 1.12	0.566						
WTR × Individualism				1.11	1.05 – 1.17	<0.001						
Tightness							0.87	0.73 – 1.03	0.099			
WTR × Tightness							0.97	0.93 – 1.03	0.356			
Relational mobility										1.44	1.18 – 1.76	<0.001
WTR × Relational mobility										1.07	1.00 – 1.14	0.062
**Random Effects**												
σ^2	3.29			3.29			3.29			3.29		
τ_{00}	$0.32_{society}$			$0.34_{society}$			$0.33_{society}$			$0.29_{society}$		
τ_{11}	$0.01_{society.WTR}$			$0.01_{society.WTR}$			$0.01_{society.WTR}$			$0.01_{society.WTR}$		
ρ_{01}	$0.31_{society}$			$0.31_{society}$			$0.16_{society}$			$0.34_{society}$		
ICC	0.09			0.10			0.09			0.08		
N	$61_{society}$			$57_{society}$			$41_{society}$			$29_{society}$		
Observations	35,448			34,243			26,399			20,985		
Marginal R^2 / Conditional R^2	0.16 / 0.23			0.16 / 0.24			0.16 / 0.24			0.18 / 0.25		

Table S41. Bayesian multilevel models on contact greeting with the interaction between WTR and historical pathogen prevalence or individualism when accounting for relationship category and societal nonindependence

Model	Parameter	LR_{Median}	95% CI	Posterior direction	R-hat	ESS
Model 66	(Intercept)	0.72	0.51 – 0.93	100%	1.00	511
	Relationship [Acquaintance]	-0.69	-0.76, -0.63	100%	1.00	3,987
	Relationship [Stranger]	-1.35	-1.42 – -1.28	100%	1.00	4,331
	Relationship [Enemy]	-1.50	-1.57 – -1.42	100%	1.00	3,928
	WTR	0.60	0.55 – 0.66	100%	1.00	4,169
	Historical pathogen prevalence	0.07	-0.19 – 0.34	70.28%	1.01	683
	Target sex [Female]	-0.17	-0.24 – -0.10	100%	1.00	3,258
	Participant sex [Female]	-0.54	-0.61 – -0.47	100%	1.00	3,222
	Sample [University]	-0.08	-0.14 – -0.03	99.88%	1.00	6,363
	Historical pathogen prevalence × WTR	-0.14	-0.22 – -0.06	99.95%	1.00	3,351
	Target sex [Female] × Participant sex [Female]	0.42	0.32 – 0.51	100%	1.00	3,108
Model 67	(Intercept)	0.56	0.31 – 0.80	100.00%	1.01	485
	Relationship [Acquaintance]	-0.69	-0.76 – -0.63	100.00%	1.00	2,720
	Relationship [Stranger]	-1.35	-1.42 – -1.28	100.00%	1.00	2,955
	Relationship [Enemy]	-1.50	-1.57 – -1.43	100.00%	1.00	2,808
	WTR	0.60	0.55 – 0.66	100.00%	1.00	2,142
	Historical pathogen prevalence	0.08	-0.12 – 0.30	77.75%	1.00	513
	Target sex [Female]	-0.17	-0.25 – -0.10	100.00%	1.00	2,081
	Participant sex [Female]	-0.54	-0.61 – -0.47	100.00%	1.00	2,194
	Sample [University]	-0.08	-0.13 – -0.02	99.65%	1.00	3,769
	Historical pathogen prevalence × WTR	-0.14	-0.22 – -0.07	100.00%	1.00	2,301
	Target sex [Female] × Participant sex [Female]	0.42	0.32 – 0.51	100.00%	1.00	1,798
Model 68	(Intercept)	0.62	0.37 – 0.90	100%	1.00	495
	Relationship [Acquaintance]	-0.69	-0.76 – -0.62	100%	1.00	3,829
	Relationship [Stranger]	-1.34	-1.41 – -1.27	100%	1.00	4,079
	Relationship [Enemy]	-1.50	-1.58 – -1.43	100%	1.00	3,921
	WTR	0.62	0.56 – 0.68	100%	1.00	3,448
	Individualism	0.08	-0.07 – 0.22	85.17%	1.00	720
	Target sex [Female]	-0.18	-0.25 – -0.10	100%	1.00	3,053
	Participant sex [Female]	-0.54	-0.61 – -0.47	100%	1.00	2,893
	Sample [University]	-0.08	-0.14 – -0.03	99.83%	1.00	4,473
	WTR × Individualism	0.10	0.05 – 0.16	99.98%	1.00	2,995
	Target sex [Female] × Participant sex [Female]	0.41	0.32 – 0.51	100%	1.00	2,716
Model 69	(Intercept)	0.70	0.47 – 0.94	100%	1.02	262
	Relationship [Acquaintance]	-0.69	-0.76 – -0.63	100%	1.00	3,929
	Relationship [Stranger]	-1.34	-1.41 – -1.27	100%	1.00	3,746
	Relationship [Enemy]	-1.50	-1.57 – -1.43	100%	1.00	3,539
	WTR	0.62	0.57 – 0.68	100%	1.00	3,517
	Individualism	-0.04	-0.23 – 0.14	66.33%	1.01	493
	Target sex [Female]	-0.18	-0.25 – -0.10	100%	1.00	2,737
	Participant sex [Female]	-0.54	-0.61 – -0.46	100%	1.00	2,994
	Sample [University]	-0.09	-0.14 – -0.03	99.92%	1.00	6,217
	WTR × Individualism	0.11	0.05 – 0.16	100%	1.00	3,800
	Target sex [Female] × Participant sex [Female]	0.41	0.32 – 0.51	100%	1.00	2,401

Note. Models 66 and 68 controlled for societal nonindependence through a Gaussian process over latitude and longitude [gp(latitude, longitude)]; Models 67 and 69 controlled for societal nonindependence through a society-level random effect whose covariance is structured by linguistic proximity [(1 | gr(society, cov = linguistic proximity))].

Table S42. Bayesian Multilevel models of contemporary pathogen prevalence index on contact comfort

Model	Parameter	b_{Median}	95% CrI	Posterior direction	R-hat	ESS
Model 70	(Intercept)	3.39	3.27 – 3.52	100%	1.000	4,206
	Relationship [Acquaintance]	-0.68	-0.72 – -0.64	100%	1.000	7,280
	Relationship [Stranger]	-1.39	-1.43 – -1.34	100%	1.000	6,490
	Relationship [Enemy]	-1.40	-1.45 – -1.36	100%	1.000	6,595
	WTR	0.75	0.70 – 0.80	100%	1.001	4,469
	Contemporary pathogen prevalence index	-0.02	-0.10 – 0.05	74.13%	1.000	3,584
	Sample [University]	0.29	0.26 – 0.33	100%	0.999	12,235
	Target sex [Female]	0.45	0.41 – 0.50	100%	1.000	6,464
	Participant sex [Female]	-0.17	-0.21 – -0.13	100%	1.000	6,865
	Target sex [Female] × Participant sex [Female]	-0.07	-0.13 – -0.01	98.30%	1.000	6,197
Model 71	(Intercept)	2.45	2.33 – 2.56	100%	1.001	5,206
	WTR	1.13	1.08 – 1.19	100%	1.001	3,924
	Contemporary pathogen prevalence index	-0.02	-0.10 – 0.05	71.60%	1.000	4,346
	Sample [University]	0.27	0.24 – 0.31	100%	1.000	14,308
	Target sex [Female]	0.40	0.36 – 0.45	100%	1.000	6,646
	Participant sex [Female]	-0.16	-0.21 – -0.12	100%	1.000	8,043
	WTR × Contemporary pathogen prevalence index	-0.07	-0.12 – -0.02	99.30%	1.001	3,797
	Target sex [Female] × Participant sex [Female]	-0.03	-0.09 – 0.03	83.23%	1.000	5,911
Model 72	(Intercept)	3.79	3.65 – 3.94	100%	1.003	1,737
	Relationship [Acquaintance]	-0.81	-0.89 – -0.72	100%	1.002	1,214
	Relationship [Stranger]	-1.58	-1.69 – -1.48	100%	1.003	968
	Relationship [Enemy]	-1.77	-1.86 – -1.67	100%	1.002	1,071
	Contemporary pathogen prevalence index	-0.13	-0.24 – -0.02	98.65%	1.000	1,448
	Sample [University]	0.31	0.28 – 0.34	100%	0.999	10,673
	Target sex [Female]	0.51	0.47 – 0.55	100%	1.000	4,365
	Participant sex [Female]	-0.21	-0.25 – -0.17	100%	1.000	4,589
	Relationship [Acquaintance] × Contemporary pathogen prevalence index	0.09	0.01 – 0.18	98.45%	1.000	1,336
	Relationship [Stranger] × Contemporary pathogen prevalence index	0.13	0.03 – 0.24	99.27%	1.000	1,183
	Relationship [Enemy] × Contemporary pathogen prevalence index	0.14	0.04 – 0.23	99.57%	1.000	1,322
	Target sex [Female] × Participant sex [Female]	-0.12	-0.18 – -0.07	100%	1.001	3,745

Note. All models controlled for societal nonindependence by adding both a Gaussian process over latitude and longitude [gp(latitude, longitude)] and a society-level random effect whose covariance is structured by linguistic proximity [(1 | gr(society, cov = linguistic proximity))].

Table S43. Bayesian Multilevel models of average death rate on contact comfort

Model	Parameter	b_{Median}	95% CrI	Posterior direction	R-hat	ESS
Model 73	(Intercept)	3.39	3.24 – 3.54	100%	1.000	4,980
	Relationship [Acquaintance]	-0.70	-0.75 – -0.66	100%	1.000	8,625
	Relationship [Stranger]	-1.44	-1.49 – -1.40	100%	1.000	7,825
	Relationship [Enemy]	-1.45	-1.50 – -1.41	100%	1.000	7,630
	WTR	0.77	0.72 – 0.82	100%	1.000	5,261
	Average death rate	0.02	-0.05 – 0.09	74.53%	1.001	4,256
	Sample [University]	0.34	0.31 – 0.38	100%	1.000	14,543
	Target sex [Female]	0.48	0.43 – 0.53	100%	1.000	8,228
	Participant sex [Female]	-0.15	-0.19 – -0.11	100%	1.000	7,568
	Target sex [Female] × Participant sex [Female]	-0.10	-0.16 – -0.03	99.83%	1.000	7,631
Model 74	(Intercept)	2.40	2.25 – 2.55	100%	1.000	3,505
	WTR	1.18	1.12 – 1.23	100%	1.000	3,544
	Average death rate	0.03	-0.04 – 0.09	77.02%	1.001	4,577
	Sample [University]	0.32	0.28 – 0.35	100%	1.000	9,114
	Target sex [Female]	0.43	0.38 – 0.48	100%	1.000	5,399
	Participant sex [Female]	-0.14	-0.19 – -0.09	100%	1.000	5,855
	WTR × Average death rate	-0.01	-0.06 – 0.04	67.80%	1.000	5,174
	Target sex [Female] × Participant sex [Female]	-0.07	-0.13 – 0.00	97.47%	1.000	5,327
Model 75	(Intercept)	3.79	3.62 – 3.97	100%	1.002	1,692
	Relationship [Acquaintance]	-0.84	-0.93 – -0.74	100%	1.003	1,491
	Relationship [Stranger]	-1.64	-1.75 – -1.52	100%	1.003	1,313
	Relationship [Enemy]	-1.83	-1.93 – -1.73	100%	1.004	1,330
	Average death rate	-0.01	-0.12 – 0.09	58.43%	1.001	1,984
	Sample [University]	0.36	0.33 – 0.40	100%	1.000	9,046
	Target sex [Female]	0.55	0.50 – 0.59	100%	1.000	5,513
	Participant sex [Female]	-0.19	-0.23 – -0.14	100%	1.000	5,450
	Relationship [Acquaintance] × Average death rate	0.05	-0.03 – 0.13	88.05%	1.001	2,205
	Relationship [Stranger] × Average death rate	0.04	-0.06 – 0.13	75.75%	1.002	2,086
	Relationship [Enemy] × Average death rate	0.04	-0.05 – 0.13	81.97%	1.002	2,051
	Target sex [Female] × Participant sex [Female]	-0.15	-0.21 – -0.09	100%	1.000	4,822

Note. All models controlled for societal nonindependence by adding both a Gaussian process over latitude and longitude [gp(latitude, longitude)] and a society-level random effect whose covariance is structured by linguistic proximity [(1 | gr(society, cov = linguistic proximity))].

Table S44. Estimated marginal means of contact comfort by levels of contemporary pathogen prevalence index or average death rate and all Tukey-adjusted pairwise comparisons within relationship category

	Relationship	Levels of contemporary pathogen prevalence	Model 72 (contemporary pathogen prevalence index)		Model 75 (average death rate)	
			Mdn	95% HPD	*Mdn*	95% HPD
Estimated marginal means	Friend	-1 SD (-1)	3.99	3.60 – 4.38	4.12	3.79 – 4.41
		Mean (0)	3.86	3.48 – 4.22	4.11	3.81 – 4.37
		+1 SD (1)	3.73	3.34 – 4.09	4.09	3.80 – 4.38
	Acquaintance	-1 SD (-1)	3.09	2.71 – 3.46	3.23	2.91 – 3.50
		Mean (0)	3.05	2.69 – 3.41	3.27	3.00 – 3.55
		+1 SD (1)	3.01	2.66 – 3.38	3.31	3.02 – 3.58
	Stranger	-1 SD (-1)	2.27	1.88 – 2.61	2.45	2.14 – 2.71
		Mean (0)	2.28	1.89 – 2.61	2.47	2.18 – 2.72
		+1 SD (1)	2.28	1.92 – 2.63	2.49	2.21 – 2.76
	Enemy	-1 SD (-1)	2.08	1.69 – 2.44	2.25	1.94 – 2.51
		Mean (0)	2.09	1.72 – 2.43	2.28	1.98 – 2.52
		+1 SD (1)	2.10	1.72 – 2.44	2.30	2.01 – 2.56
	**Relationship**	**Contrast (A − B)**	**Model 72 (contemporary pathogen prevalence index)**		**Model 75 (average death rate)**	
			**Δ*Mdn***	**95% HPD**	**Δ*Mdn***	**95% HPD**
Pairwise contrasts	Friend	-1 SD (-1) − Mean (0)	0.13	0.02 – 0.24	0.01	-0.10 – 0.11
		-1 SD (-1) − +1 SD (1)	0.26	0.04 – 0.49	0.02	-0.19 – 0.23
		Mean (0) − +1 SD (1)	0.13	0.02 – 0.24	0.01	-0.10 – 0.11
	Acquaintance	-1 SD (-1) − Mean (0)	0.04	-0.05 – 0.12	-0.04	-0.12 – 0.05
		-1 SD (-1) − +1 SD (1)	0.07	-0.10 – 0.24	-0.07	-0.24 – 0.10
		Mean (0) − +1 SD (1)	0.04	-0.05 – 0.12	-0.04	-0.12 – 0.05
	Stranger	-1 SD (-1) − Mean (0)	-0.001	-0.08 – 0.07	-0.02	-0.10 – 0.06
		-1 SD (-1) − +1 SD (1)	-0.001	-0.16 – 0.15	-0.05	-0.20 – 0.11
		Mean (0) − +1 SD (1)	-0.001	-0.08 – 0.07	-0.02	-0.10 – 0.06
	Enemy	-1 SD (-1) − Mean (0)	-0.01	-0.10 – 0.07	-0.03	-0.10 – 0.04
		-1 SD (-1) − +1 SD (1)	-0.01	-0.19 – 0.15	-0.06	-0.21 – 0.08
		Mean (0) − +1 SD (1)	-0.01	-0.10 – 0.07	-0.03	-0.10 – 0.04

Note. Pairwise comparisons were based on Models 72 and 75. Mdn represents the posterior median of the estimated marginal mean; ΔMdn represents the posterior median of the difference (A − B); HPD represents the highest posterior density interval. A contrast is considered credible when its 95% HPD interval excludes zero.

Table S45. Bayesian Multilevel models of contemporary pathogen prevalence index on contact greeting

Model	Parameter	LR_{Median}	95% CrI	Posterior direction	R-hat	ESS
Model 76	(Intercept)	0.57	-0.75 – 2.01	90.22%	1.013	1,094
	Relationship [Acquaintance]	-0.69	-0.76 – -0.63	100%	1.001	4,181
	Relationship [Stranger]	-1.35	-1.42 – -1.28	100%	1.000	4,044
	Relationship [Enemy]	-1.51	-1.58 – -1.44	100%	1.000	4,087
	WTR	0.62	0.56 – 0.68	100%	1.000	3,521
	Contemporary pathogen prevalence index	0.09	-0.04 – 0.25	90.05%	1.003	506
	Sample [University]	-0.10	-0.15 – -0.04	99.97%	1.000	4,905
	Target sex [Female]	-0.15	-0.22 – -0.07	99.98%	1.001	3,426
	Participant sex [Female]	-0.51	-0.58 – -0.44	100%	1.001	3,615
	Target sex [Female] × Participant sex [Female]	0.38	0.28 – 0.48	100%	1.001	3,158
Model 77	(Intercept)	-0.44	-0.67 – -0.21	100%	1.004	1,093
	WTR	0.98	0.92 – 1.05	100%	1.000	6,164
	Contemporary pathogen prevalence index	0.09	-0.001 – 0.18	97.30%	1.000	2,303
	Sample [University]	-0.11	-0.17 – -0.06	100%	1.000	11,933
	Target sex [Female]	-0.18	-0.25 – -0.11	100%	1.000	6,558
	Participant sex [Female]	-0.46	-0.53 – -0.39	100%	1.000	6,637
	WTR × Contemporary pathogen prevalence index	-0.14	-0.20 – -0.07	100%	1.002	5,647
	Target sex [Female] × Participant sex [Female]	0.38	0.29 – 0.48	100%	1.000	5,902
Model 78	(Intercept)	0.91	-0.88 – 2.44	91.21%	1.004	1,811
	Relationship [Acquaintance]	-0.80	-0.88 – -0.71	100%	1.000	3,539
	Relationship [Stranger]	-1.51	-1.63 – -1.39	100%	1.001	2,234
	Relationship [Enemy]	-1.80	-1.93 – -1.68	100%	1.001	2,444
	Contemporary pathogen prevalence index	0.01	-0.14 – 0.18	54.36%	1.002	881
	Sample [University]	-0.09	-0.14 – -0.03	99.94%	1.000	9,595
	Target sex [Female]	-0.09	-0.15 – -0.02	99.34%	1.000	4,873
	Participant sex [Female]	-0.52	-0.58 – -0.46	100%	1.000	5,364
	Relationship [Acquaintance] × Contemporary pathogen prevalence index	0.12	0.03 – 0.20	99.60%	1.000	3,340
	Relationship [Stranger] × Contemporary pathogen prevalence index	0.02	-0.10 – 0.14	64.56%	1.001	2,278
	Relationship [Enemy] × Contemporary pathogen prevalence index	0.12	-0.002 – 0.24	97.29%	1.001	2,044
	Target sex [Female] × Participant sex [Female]	0.35	0.26 – 0.43	100%	1.000	4,476

Note. Models 76 and 78 controlled for societal nonindependence by adding both a Gaussian process over latitude and longitude [gp(latitude, longitude)] and a society-level random effect whose covariance is structured by linguistic proximity [(1 | gr(society, cov = linguistic proximity))]. Model 77 only controlled for societal linguistic proximity to ensure convergence.

Table S46. Bayesian Multilevel models of average death rate on contact greeting

Model	Parameter	LR_{Median}	95% CI	Posterior direction	R-hat	ESS
Model 79	(Intercept)	0.53	-0.40 – 1.54	92.95%	1.002	1,323
	Relationship [Acquaintance]	-0.73	-0.80 – -0.65	100%	1.000	4,569
	Relationship [Stranger]	-1.39	-1.46 – -1.31	100%	1.001	4,552
	Relationship [Enemy]	-1.57	-1.65 – -1.49	100%	1.000	4,539
	WTR	0.63	0.57 – 0.70	100%	1.001	3,877
	Average death rate	0.01	-0.13 – 0.18	57.58%	1.006	946
	Sample [University]	-0.03	-0.10 – 0.03	86.90%	1.000	6,641
	Target sex [Female]	-0.15	-0.23 – -0.07	100%	1.000	3,999
	Participant sex [Female]	-0.52	-0.60 – -0.44	100%	1.000	3,788
	Target sex [Female] × Participant sex [Female]	0.34	0.23 – 0.45	100%	1.000	3,501
Model 80	(Intercept)	-0.46	-1.72 – 0.81	88.15%	0.999	2,356
	WTR	1.02	0.94 – 1.10	100%	1.000	4,090
	Average death rate	0.01	-0.12 – 0.16	57.67%	1.006	1,025
	Sample [University]	-0.06	-0.12 – 0.00	98.08%	1.000	8,745
	Target sex [Female]	-0.19	-0.27 – -0.11	100%	1.001	4,151
	Participant sex [Female]	-0.47	-0.54 – -0.39	100%	1.000	4,179
	WTR × Average death rate	-0.03	-0.10 – 0.04	81.07%	1.000	5,167
	Target sex [Female] × Participant sex [Female]	0.34	0.24 – 0.45	100%	1.001	3,735
Model 81	(Intercept)	0.60	-0.74 – 1.84	92.43%	1.003	2,100
	Relationship [Acquaintance]	-0.72	-0.83 – -0.61	100%	1.000	3,514
	Relationship [Stranger]	-1.39	-1.52 – -1.27	100%	1.003	2,237
	Relationship [Enemy]	-1.58	-1.73 – -1.44	100%	1.002	2,446
	Average death rate	0.02	-0.15 – 0.19	56.93%	1.000	1,364
	WTR	0.63	0.57 – 0.68	100%	1.000	9,824
	Sample [University]	-0.03	-0.10 – 0.03	86.18%	1.000	8,879
	Target sex [Female]	-0.16	-0.24 – -0.07	99.98%	1.000	4,608
	Participant sex [Female]	-0.52	-0.59 – -0.44	100%	1.000	4,453
	Relationship [Acquaintance] × Average death rate	0.07	-0.03 – 0.17	91.38%	1.000	3,689
	Relationship [Stranger] × Average death rate	-0.04	-0.16 – 0.07	77.07%	1.000	2,698
	Relationship [Enemy] × Average death rate	0.00	-0.13 – 0.14	52.60%	1.000	2,877
	Target sex [Female] × Participant sex [Female]	0.35	0.24 – 0.45	100%	1.000	4,053

Note. All models controlled for societal nonindependence by adding both a Gaussian process over latitude and longitude [gp(latitude, longitude)] and a society-level random effect whose covariance is structured by linguistic proximity [(1 | gr(society, cov = linguistic proximity))].

Table S47. Estimated marginal means of contact greeting by levels of contemporary pathogen prevalence index or average death rate and all Tukey-adjusted pairwise comparisons within relationship category

	Relationship	Levels of contemporary pathogen prevalence	Model 78 (contemporary pathogen prevalence index)		Model 81 (average death rate)	
			Mdn	**95% HPD**	***Mdn***	**95% HPD**
Estimated marginal means	Friend	-1 SD (-1)	0.88	0.42 – 1.27	0.64	0.16 – 1.10
		Mean (0)	0.89	0.52 – 1.23	0.65	0.23 – 1.06
		+1 SD (1)	0.89	0.52 – 1.24	0.66	0.22 – 1.09
	Acquaintance	-1 SD (-1)	-0.03	-0.50 – 0.35	-0.15	-0.63 – 0.30
		Mean (0)	0.09	-0.31 – 0.41	-0.07	-0.48 – 0.35
		+1 SD (1)	0.21	-0.14 – 0.56	0.01	-0.45 – 0.42
	Stranger	-1 SD (-1)	-0.64	-1.11 – -0.27	-0.71	-1.18 – -0.27
		Mean (0)	-0.62	-0.99 – -0.29	-0.74	-1.15 – -0.33
		+1 SD (1)	-0.59	-0.94 – -0.26	-0.77	-1.19 – -0.35
	Enemy	-1 SD (-1)	-1.04	-1.50 – -0.66	-0.94	-1.41 – -0.48
		Mean (0)	-0.91	-1.29 – -0.59	-0.93	-1.36 – -0.53
		+1 SD (1)	-0.79	-1.14 – -0.45	-0.91	-1.34 – -0.48
	**Relationship**	**Contrast (A − B)**	**Model 78 (contemporary pathogen prevalence index)**		**Model 81 (average death rate)**	
			**Δ*Mdn***	**95% HPD**	**Δ*Mdn***	**95% HPD**
Pairwise contrasts	Friend	-1 SD (-1) − Mean (0)	-0.01	-0.17 – 0.15	-0.02	-0.20 – 0.14
		-1 SD (-1) − +1 SD (1)	-0.02	-0.34 – 0.30	-0.03	-0.41 – 0.28
		Mean (0) − +1 SD (1)	-0.01	-0.17 – 0.15	-0.02	-0.20 – 0.14
	Acquaintance	-1 SD (-1) − Mean (0)	-0.13	-0.29 – 0.02	-0.09	-0.25 – 0.08
		-1 SD (-1) − +1 SD (1)	-0.25	-0.58 – 0.05	-0.17	-0.51 – 0.17
		Mean (0) − +1 SD (1)	-0.13	-0.29 – 0.02	-0.09	-0.25 – 0.08
	Stranger	-1 SD (-1) − Mean (0)	-0.03	-0.18 – 0.11	0.03	-0.13 – 0.18
		-1 SD (-1) − +1 SD (1)	-0.06	-0.36 – 0.21	0.06	-0.26 – 0.35
		Mean (0) − +1 SD (1)	-0.03	-0.18 – 0.11	0.03	-0.13 – 0.18
	Enemy	-1 SD (-1) − Mean (0)	-0.13	-0.28 – 0.02	-0.02	-0.18 – 0.14
		-1 SD (-1) − +1 SD (1)	-0.25	-0.56 – 0.03	-0.04	-0.37 – 0.28
		Mean (0) − +1 SD (1)	-0.13	-0.28 – 0.02	-0.02	-0.18 – 0.14

Note. Pairwise comparisons were based on Models 78 and 81. Estimates are on the log odds ratio scale. Mdn represents the posterior median of the estimated marginal mean; ΔMdn represents the posterior median of the difference (A − B); HPD represents the highest posterior density interval. A contrast is considered credible when its 95% HPD interval excludes zero.

SI References

1. R. Neel, D. T. Kenrick, A. E. White, S. L. Neuberg, Individual differences in fundamental social motives. *J. Pers. Soc. Psychol.* **110**, 887–907 (2016).

2. A. W. Delton, *et al.*, Cognitive foundations for helping and harming others: Making welfare tradeoffs in industrialized and small-scale societies. *Evol. Hum. Behav.* **44**, 485–501 (2023).

3. A. W. Delton, T. E. Robertson, How the mind makes welfare tradeoffs: evolution, computation, and emotion. *Curr. Opin. Psychol.* **7**, 12–16 (2016).

4. J. M. Tybur, D. Lieberman, L. Fan, T. R. Kupfer, R. E. de Vries, Behavioral Immune Trade-Offs: Interpersonal Value Relaxes Social Pathogen Avoidance. *Psychol. Sci.* **31**, 1211–1221 (2020).

5. D. R. Murray, M. Schaller, Historical Prevalence of Infectious Diseases Within 230 Geopolitical Regions: A Tool for Investigating Origins of Culture. *J. Cross-Cult. Psychol.* **41**, 99–108 (2010).

6. G. Hofstede, G. J. Hofstede, M. Minkov, *Cultures and organizations: software of the mind: intercultural cooperation and its importance for survival*, 3rd ed (McGraw-Hill, 2010).

7. M. J. Gelfand, *et al.*, Differences Between Tight and Loose Cultures: A 33-Nation Study. *Science* **332**, 1100–1104 (2011).

8. M. J. Gelfand, *et al.*, The relationship between cultural tightness–looseness and COVID-19 cases and deaths: a global analysis. *Lancet Planet. Health* **5**, e135–e144 (2021).

9. R. Thomson, *et al.*, Relational mobility predicts social behaviors in 39 countries and is tied to historical farming and threat. *Proc. Natl. Acad. Sci.* **115**, 7521–7526 (2018).

10. C. L. Fincher, R. Thornhill, Parasite-stress promotes in-group assortative sociality: The cases of strong family ties and heightened religiosity. *Behav. Brain Sci.* **35**, 61–79 (2012).

11. J. M. Tybur, D. Lieberman, V. Griskevicius, Microbes, mating, and morality: Individual differences in three functional domains of disgust. *J. Pers. Soc. Psychol.* **97**, 103–122 (2009).

12. D. L. Putnick, M. H. Bornstein, Measurement Invariance Conventions and Reporting: The State of the Art and Future Directions for Psychological Research. *Dev. Rev. DR* **41**, 71–90 (2016).

13. Y. Rosseel, lavaan: An R Package for Structural Equation Modeling. *J. Stat. Softw.* **48**, 1–36 (2012).

14. G. Hirschfeld, von B. Ruth, Multiple-Group confirmatory factor analysis in R – A tutorial in measurement invariance with continuous and ordinal indicators. *Pract. Assess. Res. Eval.* **19**, 1–12 (2014).

15. W. Viechtbauer, Conducting Meta-Analyses in *R* with the **metafor** Package. *J. Stat. Softw.* **36** (2010).

16. J. Hox, M. Moerbeek, R. van de Schoot, *Multilevel Analysis: Techniques and Applications*, 3rd Ed. (Routledge, 2017).

17. D. Bates, M. Mächler, B. Bolker, S. Walker, Fitting Linear Mixed-Effects Models using lme4. [Preprint] (2014). Available at: http://arxiv.org/abs/1406.5823 [Accessed 2 August 2023].

18. A. Kuznetsova, P. B. Brockhoff, R. H. B. Christensen, lmerTest Package: Tests in Linear Mixed Effects Models. *J. Stat. Softw.* **82**, 1–26 (2017).

19. J. B. Kadane, N. A. and Lazar, Methods and Criteria for Model Selection. *J. Am. Stat. Assoc.* **99**, 279–290 (2004).

20. M. A. Stoffel, S. Nakagawa, H. Schielzeth, partR2: Partitioning R2 in GLMMs. (2024). Deposited 4 March 2024.

21. J. A. Long, interactions: Comprehensive, User-Friendly Toolkit for Probing Interactions. (2024). Deposited 29 July 2024.

22. R. V. Lenth, *et al.*, emmeans: Estimated Marginal Means, aka Least-Squares Means. (2024). Deposited 20 May 2024.

23. P.-C. Bürkner, **brms** : An *R* Package for Bayesian Multilevel Models Using *Stan*. *J. Stat. Softw.* **80** (2017).

24. S. Claessens, T. Kyritsis, Q. D. Atkinson, Cross-national analyses require additional controls to account for the non-independence of nations. *Nat. Commun.* **14**, 5776 (2023).

25. J. J. Dziak, I. Nahum-Shani, L. M. Collins, Multilevel factorial experiments for developing behavioral interventions: Power, sample size, and resource considerations. *Psychol. Methods* **17**, 153–175 (2012).

26. M. G. Arend, T. Schäfer, Statistical power in two-level models: A tutorial based on Monte Carlo simulation. *Psychol. Methods* **24**, 1–19 (2019).

27. S. I. Vrieze, Model selection and psychological theory: A discussion of the differences between the Akaike information criterion (AIC) and the Bayesian information criterion (BIC). *Psychol. Methods* **17**, 228–243 (2012).
